# The Mechanism of High-Output Cardiac Hypertrophy Arising From Potassium Channel Gain-of-Function in Cantú Syndrome

**DOI:** 10.1093/function/zqaa004

**Published:** 2020-06-18

**Authors:** Conor McClenaghan, Yan Huang, Scot J Matkovich, Attila Kovacs, Carla J Weinheimer, Ron Perez, Thomas J Broekelmann, Theresa M Harter, Jin-Moo Lee, Maria S Remedi, Colin G Nichols

**Affiliations:** 1 Center for the Investigation of Membrane Excitability Diseases, Washington University School of Medicine, St. Louis, MO 63110, USA; 2 Departments of Cell Biology and Physiology, Washington University School of Medicine, St. Louis, MO 63110, USA; 3 Medicine, Washington University School of Medicine, St. Louis, MO 63110, USA; 4 Neurology, Washington University School of Medicine, St. Louis, MO 63110, USA

**Keywords:** KATP, KCNJ8, Kir6.1, ABCC9, SUR2, Cantú, syndrome, renin, angiotensin, smooth muscle, blood pressure, high-output heart failure, channelopathy

## Abstract

Dramatic cardiomegaly arising from gain-of-function (GoF) mutations in the ATP-sensitive potassium (K_ATP_) channels genes, *ABCC9* and *KCNJ8*, is a characteristic feature of Cantú syndrome (CS). How potassium channel over-activity results in cardiac hypertrophy, as well as the long-term consequences of cardiovascular remodeling in CS, is unknown. Using genome-edited mouse models of CS, we therefore sought to dissect the pathophysiological mechanisms linking K_ATP_ channel GoF to cardiac remodeling. We demonstrate that chronic reduction of systemic vascular resistance in CS is accompanied by elevated renin–angiotensin signaling, which drives cardiac enlargement and blood volume expansion. Cardiac enlargement in CS results in elevation of basal cardiac output, which is preserved in aging. However, the cardiac remodeling includes altered gene expression patterns that are associated with pathological hypertrophy and are accompanied by decreased exercise tolerance, suggestive of reduced cardiac reserve. Our results identify a high-output cardiac hypertrophy phenotype in CS which is etiologically and mechanistically distinct from other myocardial hypertrophies, and which exhibits key features of high-output heart failure (HOHF). We propose that CS is a genetically-defined HOHF disorder and that decreased vascular smooth muscle excitability is a novel mechanism for HOHF pathogenesis.

## Introduction

Cantú Syndrome (CS) is a rare congenital disorder associated with a range of cardiovascular (CV) abnormalities, including dilated and tortuous blood vessels, pericardial effusion, and dramatically enlarged hearts, as well as frequent reports of pulmonary hypertension (PH) and exercise intolerance.^1–7^ CS is caused by gain-of-function (GoF) mutations in *ABCC9* or *KCNJ8*, which encode the SUR2 and Kir6.1 subunits of CV ATP-sensitive potassium (K_ATP_) channels, respectively.^1^^,^^7–9^ Kir6.1 and SUR2 are coexpressed in smooth muscle cells, where K_ATP_ channel activation reduces cellular excitability and provokes vasodilation.^10–13^ Thus, vascular smooth muscle cell K_ATP_ channel GoF provides a ready explanation for the chronic vasodilation observed in CS patients. In contrast, cardiac hypertrophy is not trivially explained by any understood mechanism arising from over-activity of a potassium channel. 

As SUR2 is expressed in both the heart and the vasculature, SUR2 mutations are expected to cause GoF of KATP in both locations. Notably, cardiomyocyte K_ATP_ channels are predominantly comprised not of Kir6.1, but the related Kir6.2 subunit, together with the SUR2A splice variant^14–18^ , but essentially the same high-output cardiac enlargement is observed in patients with either Kir6.1 or SUR2 GoF substitutions.^1^^,^^3^^,^^8^^,^^18^ Cardiac hypertrophy is also present in genome-edited “Cantú mice,” in which human disease-associated mutations were introduced into either the endogenous *ABCC9* or *KCNJ8* genes, despite no apparent alteration of K_ATP_ channel properties in the ventricular myocytes of *KCNJ8*-mutant (Kir6.1^wt/VM^) mice.^19^ These results imply that cardiac hypertrophy must arise secondarily to K_ATP_ dysfunction outside of the heart, potentially as a compensation to normalize tissue perfusion in the face of persistent vasodilation and reduced systemic vascular resistance. Consistent with this hypothesis, we recently demonstrated that downregulation of K_ATP_ function in smooth muscle, using transgenic, tissue-specific, dominant-negative Kir6.1 subunits, reverses cardiac hypertrophy in *ABCC9*-mutant (SUR2^wt/AV^) mice.^20^ However, the mechanistic link between K_ATP_ GoF in smooth muscle and cardiac remodeling is not known.

high-output, hypertrophic cardiac remodeling. Given the relatively recent genetic identification of CS, little is known about the long-term potential of CV pathophysiology in CS, and whether this compensatory cardiac remodeling is ultimately pathological is unclear. We show that elevated basal cardiac function is maintained with aging in CS mice but is accompanied by hallmarks of high-output heart failure (HOHF), including upregulation of transcriptional markers of pathological hypertrophy, blood volume expansion, elevated right ventricular systolic pressures (RVSPs), and exercise intolerance.

## Results

### Cardiac Hypertrophy in Cantú Mice

**Figure 1.**
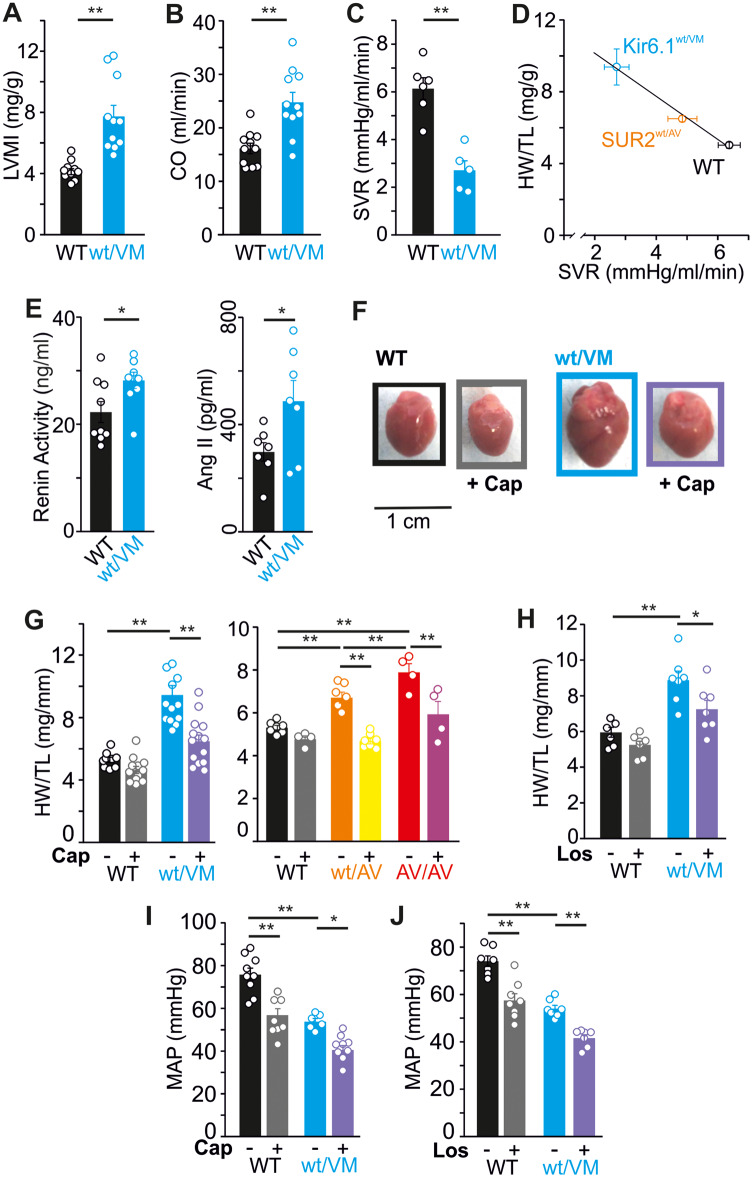
Cardiac Hypertrophy in Cantú Mice Is Driven by RAS. (**A**) Left ventricular mass from echocardiographic analysis and indexed to body weight (LVMI) and (**B**) cardiac output (CO) are significantly elevated, and systemic vascular resistance (SVR) is significantly reduced (**C**) in Kir6.1^wt/VM^ mice. (**D**) Heart size (heart weight to tibia length; HW/TL) and SVR is strongly negatively correlated in WT (*n* = 12), SUR2^wt/AV^ (*n* = 7), and Kir6.1^wt/VM^ (*n* = 5) mice (Pearson correlation coefficient = −1.0). Data from SUR2^wt/AV^ mice originally reported in Figure 2A and C in McClenaghan et al.^20^ (**E**) ELISA shows upregulation of plasma renin activity (left) and plasma Ang II (right) in Kir6.1^wt/VM^ mice. Administration of captopril (Cap) results in significant reduction of heart size in Kir6.1^wt/VM^ (**F, G**), and SUR2^wt/AV^ and SUR2^AV/AV^ mice (G). (**H**) Administration of losartan (Los) results in significant reversal of cardiac hypertrophy in Kir6.1^wt/VM^ mice. Captopril (**I**) and losartan (**J**) both significantly reduce mean arterial pressures (MAP) in both WT and Kir6.1^wt/VM^ mice. For all figures, individual data points are represented as open circles, bars show mean ± SEM. Statistical significance was determined by Student’s *t*-test (A–C, E) or one-way ANOVA and post hoc Tukey’s test for pairwise comparison (G–J). *P < 0.05; **P < 0.01.

Previously, we reported significant vascular abnormalities and cardiac remodeling in heterozygous Kir6.1(V65M) mice (Kir6.1^wt/VM^) as well as in both heterozygous and homozygous SUR2(A478V) mice (SUR2^wt/AV^ and SUR2^AV/AV^, respectively).^19^^,^^20^ CS-associated mutations in SUR2 or Kir6.1 both result in marked cardiac enlargement in mice but, even with the most dramatic enlargement seen in Kir6.1^wt/VM^ Cantú mice, this is not accompanied by decreased ejection fraction; on the contrary, cardiac output is proportionately elevated (Figure 1A and B). Echocardiography of young adult mice reveals marked structural remodeling, with approximately equivalent increases in both left ventricle (LV) chamber size and wall thickness, but only subtle trends toward altered myocardial deformation and some evidence of increased LV filling pressure with no indication of major systolic or diastolic dysfunction at rest (Table 1).

**Table 1.** Summary of echocardiographic measurements from young and old adult WT and Kir6.1^wt/VM^ mice

	Young adult mice (3–5 months old)			Old mice (12 month old)		
	WT (*n* = 11)	wt/VM (*n* = 11)	*P*-value	WT (*n* = 8)	wt/VM (*n* = 8)	*P*-value
HR, bpm	622.8 ± 9.1	622.8 ± 6.9	1.000	637.0 ± 14.5	594.4 ± 12.7	0.048^*^
BW, g	24.1 ± 0.5	26.5 ± 1.1	0.071	32.7 ± 1.5	30.7 ± 0.8	0.261
LVPWd, mm	0.89 ± 0.02	1.18 ± 0.06	<0.001^*^	0.89 ± 0.02	1.19 ± 0.03	<0.001^*^
IVSd, mm	0.95 ± 0.02	1.18 ± 0.06	0.002^*^	0.97 ± 0.02	1.22 ± 0.03	<0.001^*^
LVIDd, mm	3.19 ± 0.07	3.99 ± 0.14	<0.001^*^	3.31 ± 0.13	3.89 ± 0.17	0.016
LVPWs, mm	1.45 ± 0.05	1.89 ± 0.08	<0.001^*^	1.34 ± 0.04	1.82 ± 0.07	<0.001^*^
IVSs, mm	1.49 ± 0.05	1.91 ± 0.09	<0.001^*^	1.36 ± 0.04	1.84 ± 0.08	<0.001^*^
LVIDs, mm	1.54 ± 0.05	1.98 ± 0.08	<0.001^*^	1.83 ± 0.10	1.90 ± 0.06	0.558
LVM, mg	99.7 ± 5.7	205.5 ± 21.2	<0.001^*^	106.8 ± 5.8	200.7 ± 16.0	<0.001^*^
LVMi, mg/g	4.1 ± 0.2	7.7 ± 0.7	<0.001^*^	3.27 ± 0.1	6.61 ± 0.6	<0.001^*^
RWT	0.58 ± 0.02	0.60 ± 0.03	0.581	0.57 ± 0.03	0.63 ± 0.03	0.223
FS (%)	51.6 ± 1.3	50.2 ± 1.1	0.404	45.0 ± 1.1	50.8 ± 1.9	0.019
Doppler						
E, cm/s	740.4 ± 29.1	902.7 ± 38.7	0.003^*^	733.6 ± 27.4	947.9 ± 59.3	0.006
A, cm/s	678.0 ± 25.1	746.6 ± 45.9	0.218	704.6 ± 28.5	835.8 ± 46.3	0.030
E/A	1.10 ± 0.03	1.25 ± 0.08	0.115	1.04 ± 0.02	1.14 ± 0.05	0.071
E′, cm/s	23.5 ± 1.2	23.2 ± 1.4	0.869	24.9 ± 1.5	24.9 ± 2.6	0.997
A′, cm/s	24.7 ± 1.1	27.1 ± 1.9	0.272	26.7 ± 1.9	29.9 ± 1.7	0.224
E/E’	32.0 ± 1.6	39.6 ± 1.8	0.005^*^	30.1 ± 2.0	39.2 ± 2.1	0.009
IVCT, ms	7.3 ± 0.5	6.1 ± 0.3	0.070	6.4 ± 0.3	6.8 ± 0.5	0.504
IVRT, ms	11.3 ± 0.4	10.0 ± 0.4	0.030	10.2 ± 0.3	10.4 ± 0.3	0.652
ET, ms	40.6 ± 1.5	38.9 ± 1.1	0.360	38.9 ± 1.1	39.8 ± 1.3	0.619
Tei Index	0.46 ± 0.01	0.42 ± 0.01	0.025	0.43 ± 0.01	0.43 ± 0.01	0.628
Volumetric analysis						
EDV, µL	32.3 ± 1.7	55.4 ± 5.1	0.001^*^	37.4 ± 2.4	57.6 ± 4.6	0.002^*^
EDVi, µL/g	1.3 ± 0.1	2.1 ± 0.2	0.001^*^	1.1 ± 0.1	1.9 ± 0.2	0.002^*^
ESV, µL	7.4 ± 0.7	14.9 ± 2.0	0.002^*^	10.4 ± 1.3	15.9 ± 1.8	0.026
SV, µL	24.9 ± 1.2	40.5 ± 3.4	<0.001^*^	27.0 ± 1.3	41.7 ± 3.0	<0.001^*^
SVi, µL/g	1.0 ± 0.04	1.5 ± 0.1	0.002^*^	0.8 ± 0.02	1.4 ± 0.13	<0.001^*^
CO, mL/min	15.4 ± 0.8	24.8 ± 1.9	<0.001^*^	16.4 ± 0.8	24.8 ± 1.9	0.001^*^
CI, mL/min/g	0.64 ± 0.03	0.94 ± 0.08	0.002^*^	0.50 ± 0.01	0.82 ± 0.08	0.001^*^
EF (%)	77.0 ± 1.2	73.1 ± 1.6	0.070	72.5 ± 1.8	72.4 ± 1.3	0.957
S dV/dt, mL/s	0.94 ± 0.04	1.44 ± 0.08	<0.001^*^	0.94 ± 0.04	1.42 ± 0.07	<0.001^*^
S dV/dt / EDV, s^−1^	0.03 ± 0.001	0.03 ± 0.001	0.156	0.03 ± 0.001	0.03 ± 0.002	0.997
D dV/dt, mL/s	0.82 ± 0.05	1.41 ± 0.13	0.001^*^	1.01 ± 0.06	1.74 ± 0.15	<0.001^*^
D dV/dt / EDV, s^−1^	0.03 ± 0.001	0.03 ± 0.001	0.745	0.03 ± 0.002	0.03 ± 0.001	0.154
Strain analysis						
Strain (radial), %	40.0 ± 1.3	34.7 ± 1.2	0.008	38.8 ± 0.6	37.0 ± 1.2	0.220
Strain (long), %	20.0 ± 0.7	18.6 ± 0.7	0.182	23.4 ± 1.6	18.4 ± 1.0	0.016
Strain rate (R_sys_), s^−1^	12.5 ± 0.4	10.1 ± 0.5	<0.001^*^	10.4 ± 0.3	9.7 ± 0.4	0.227
Strain rate (R_dia_e), s^−1^	12.3 ± 0.4	12.3 ± 0.5	0.970	13.6 ± 0.8	14.9 ± 0.7	0.230
Strain rate (R_dia_a), s^−1^	5.9 ± 0.5	4.7 ± 1.2	0.388	3.4 ± 0.9	2.3 ± 0.8	0.491
Strain rate (L_sys_), s^−1^	9.9 ± 0.5	9.1 ± 0.9	0.417	9.7 ± 0.6	7.3 ± 0.5	0.009
Strain rate (L_dia_e), s^−1^	10.9 ± 0.4	11.3 ± 0.8	0.614	12.3 ± 0.6	11.6 ± 0.7	0.455
Strain rate (L_dia_a), s^−1^	3.9 ± 0.5	2.6 ± 0.4	0.055	2.4 ± 0.5	2.6 ± 0.04	0.888

* *P*-value < Bonferroni adjusted α (α for structural measurements = 0.05/10; for Doppler analysis = 0.05/10; for volumetric analysis = 0.05/12; for strain analysis = 0.05/8).

LVPWd, LV posterior wall in diastole; IVSd, interventricular septum in diastole; LVIDd, LV internal dimension in diastole; LVPWs, LV posterior wall in systole; IVSs, interventricular septum in systole; LVIDs, LV internal dimension in systole; LVM, LV mass; LVMi, LV mass indexed to body weight; RWT, relative wall thickness (LVPWd + IVSd/LVIDd); FS, fractional shortening; E, early mitral peak inflow velocity; A, late mitral inflow velocity; E/A, early to late mitral inflow ratio; E', early diastolic velocity of mitral annulus; A', late diastolic velocity of mitral annulus; E/E', ratio of E to E’; IVCT, isovolumetric contraction time; IVRT, isovolumetric relaxation time; ET, ejection time; Tei Index, cardiac performance index (IVCT + IVRT/ET); EDV, end-diastolic LV volume (μL) based on long-axis images; EDVi, end-diastolic LV volume indexed to body weight; ESV, end-systolic LV volume; SV, stroke volume; SVi, stroke volume indexed to body weight; CO, cardiac output; CI, cardiac index (CO indexed to body weight); EF, ejection fraction; S dV/dt, LV peak ejection rate during systole; S dV/dt/EDV, LV peak ejection rate during systole indexed to end diastolic volume; D dV/dt, maximum rate of early LV filling; D dV/dt/EDV, maximum rate of early LV filling indexed to end diastolic volume; Strain (radial), peak global radial LV myocardial strain; Strain (long), peak global longitudinal LV myocardial strain; Strain rate (R_sys_), peak systolic global radial LV myocardial strain rate; Strain rate (R_dia_e), peak early diastolic global radial LV myocardial strain rate; Strain rate (R_dia_a), peak late (atrial) diastolic global radial LV myocardial strain rate; Strain rate (L_sys_), peak systolic global longitudinal LV myocardial strain rate; Strain rate (L_dia_e), peak early diastolic global longitudinal LV myocardial strain rate; Strain rate (L_dia_a), peak late (atrial) diastolic global longitudinal LV myocardial strain rate. Data are presented as mean ± SEM.

### RAS Signaling Drives Cardiac Hypertrophy

Kir6.1 and SUR2 are both expressed in vascular smooth muscle, wherein activation of K_ATP_ channels provokes vasodilation, and Cantú mice exhibit significant decreases in systemic vascular resistance (Figure 1C) as well as reduced blood pressures.^19^^,^^20^ Heart size correlates inversely with both systemic vascular resistance (Figure 1D) and the extent of K_ATP_ channel activity in vascular smooth muscle cells across genotypes.^19^

Decreased blood pressure and consequent reduction in renal perfusion is a trigger for activation of the RAS, which leads to fluid retention and increased vascular tone, and thereby serves to maintain normal blood pressures. As angiotensin II (Ang II) is also known to stimulate cardiomyocyte hypertrophy, and increased RAS signaling has previously been implicated in adverse CV remodeling caused by K_ATP_-channel opening (KCO) drugs in rodent models,^21^^,^^22^ we hypothesized that RAS upregulation may contribute to cardiac enlargement in CS. We used ELISA to assess RAS signaling; both plasma renin activity and Ang II levels are significantly elevated (by ∼25% and ∼60%, respectively) in Kir6.1^wt/VM^ mice compared to wild type (WT) (Figure 1E). To assess the consequences of this elevated RAS signaling on cardiac remodeling in CS, we administered either the angiotensin-converting enzyme inhibitor (ACE-I) captopril or the angiotensin receptor blocker (ARB) losartan. While neither captopril nor losartan had significant effects on WT heart size, both caused a similarly dramatic reduction in heart size in Cantú mice (Figure 1F–H), demonstrating that RAS signaling downstream of Ang II production is responsible for cardiac hypertrophy in CS. As expected, both captopril and losartan provoked significant decreases in blood pressure in WT and Kir6.1^wt/VM^ mice (Figure 1I and J). 

### Increased Circulatory Volume and RVSP in Cantú Mice

**Figure 2.**
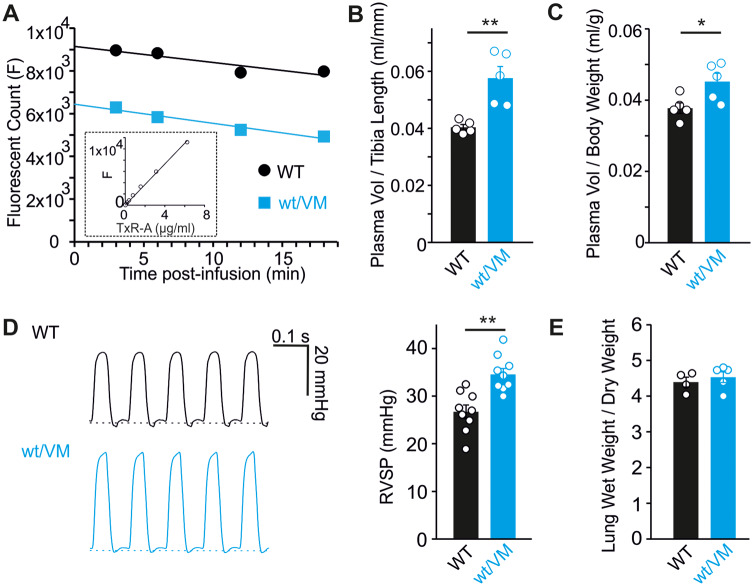
Blood Volume Expansion in Kir6.1^wt/VM^ mice. (**A**) Example data showing TxR-A fluorescence of serial blood samples in WT (black) and Kir6.1^wt/VM^ mice. A linear fit of the data points was extrapolated to time zero to estimate the initial dilution factor by comparison to a TxR-A standard curve (example in inset). Calculated plasma volume normalized to tibia length (**B**) or body weight (**C**) in WT and Kir6.1^wt/VM^ mice. (**D**) Representative right ventricular pressure traces and summary RVSP measurements from WT and Kir6.1^wt/VM^ mice. (**E**) The ratio of wet lung weight to dry lung weight for WT and Kir6.1^wt/VM^ mice. For all figures, individual data points are represented as open circles, bars show mean ± SEM. Statistical significance was determined by Student’s *t*-test; *P < 0.05; **P < 0.01.

Increased RAS signaling, in response to low blood pressure, causes retention of salt and fluid by the kidney, leading to increased blood volume. We developed a method for quantifying mouse plasma volume in which fluorescent Texas Red-conjugated albumin (TxR-A) was injected intravenously followed by serial blood sampling to measure distribution. Following injection, a slow, time-dependent decrease in TxR-A fluorescence was observed, reflecting slow loss of albumin from the vascular space. Linear extrapolation of the measured fluorescence to zero time allows estimation of the initial fluorescence, which was compared to a standard curve of known TxR-A concentrations to calculate the dilution factor and plasma volume (Figure 2A). This analysis demonstrated that plasma volume (normalized to mouse tibia length) was increased by ∼40% in Kir6.1^wt/VM^ mice, compared to WT littermate controls (Figure 2B). Kir6.1^wt/VM^ mice weigh more than controls (potentially a reflection of increased fluid retention) but even when plasma volume is normalized to body weight, a significant ∼20% increase is still apparent in Kir6.1^wt/VM^ (Figure 2C).

In addition to blood volume expansion, RVSPs, reflective of pulmonary artery pressure (PAP), are significantly increased in Kir6.1^wt/VM^ mice (Figure 2D). The presence of elevated PAPs, despite the noted *vasodilatory* effect of K_ATP_ channel activation in pulmonary vascular smooth muscle,^23^ is consistent with volume overload of the pulmonary circulation and/or increased left atrial pressures. Notably, echocardiography reveals that the E/E′ ratio (ratio of early diastolic mitral inflow velocity to early diastolic relaxation velocity at the mitral annulus; a noninvasive measure of LV filling pressure) is increased in Kir6.1^wt/VM^ mice, consistent with an increase in left atrial pressure (Table 1). Previously, we demonstrated that Kir6.1^wt/VM^ mice exhibit both aortic stenosis (AS) and aortic insufficiency (AI),^19^ both of which could contribute to the elevated pulmonary pressures. Notably, however, there is no positive correlation between RVSP and either AS mean gradient (Pearson’s coefficient −0.28, *n* = 5) or AI area (Pearson’s coefficient −0.46, *n* = 5) when measured in the same mice. Despite the increased RVSP, no increase in wet lung weights was observed in mutant mice (Figure 2E), consistent with the mild PH observed at rest under anesthetized conditions.

### Altered Transcriptional Profile in the Heart of Cantú Mice

**Figure 3.**
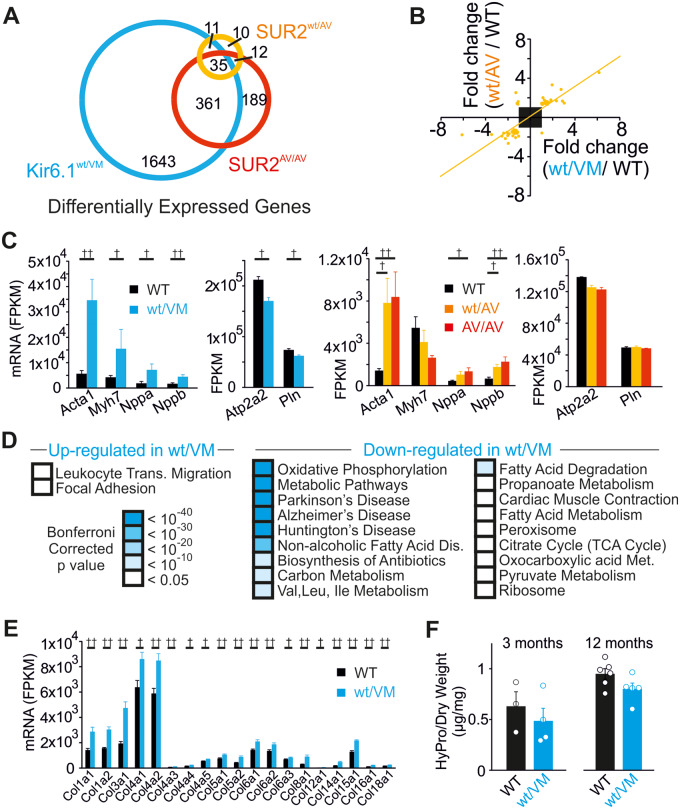
RNASeq Reveals Transcriptional Remodeling in the Cantú Mouse Heart. (**A**) Comparison of differentially expressed genes in Kir6.1^wt/VM^ (blue), SUR2^wt/AV^ (orange), and SUR2^AV/AV^ (red) mice, each compared to WT. Overlapping circles represent genes that were differentially expressed across multiple mutant lines. (**B**) Correlation of up- and downregulated genes (fold change >1.2 or <−1.2; FDR < 0.1) in Kir6.1^wt/VM^ and SUR2^wt/AV^ mice. (**C**) Upregulation (left) and downregulation (right) of characteristic markers of pathological hypertrophy in Kir6.1^wt/VM^, SUR2^wt/AV^, and SUR2^AV/AV^ mice. (**D**) KEGG pathway analysis of DEGs (fold change either <−1.2 or >1.2 for upregulated and downregulated genes; FDR < 0.1) in Kir6.1^wt/VM^ mice. (**E**) Upregulation of collagen genes in Kir6.1^wt/VM^ mice. (**F**) Ventricular hydroxyproline (HyPro) content in WT and Kir6.1^wt/VM^ mice at 3 (left) and 12 (right) months of age. ^†^FDR < 0.05, ^††^FDR < 0.01.

In addition to causing cardiac hypertrophy, chronic RAS upregulation is also associated with marked transcriptional changes and collagen deposition in the heart. We carried out RNASeq to investigate the cardiac transcriptional profile in CS mice; both CS-associated mutations provoke similar transcriptome changes (Figure 3A and B; Supplementary Information). Transcriptional changes that are recognized as markers of pathological hypertrophy were present in Kir6.1^wt/VM^ and SUR2(A478V) hearts, including significant increases in *Acta1*, *Myh7*, *Nppa*, and *Nppb* expression, and decreases in *Atp2a2* and *Pln* (Figure 3B and C).

We focused on the most severely affected Kir6.1^wt/VM^ hearts to perform pathway analysis. This reveals widespread downregulation of genes encoding proteins involved in oxidative phosphorylation, glycolysis, and lipid and amino acid metabolism (Figure 3D;Supplementary Information). Downregulation of these metabolic pathways has previously been demonstrated in various forms of pathological cardiac remodeling.^24^ As also previously reported in pathological hypertrophy, focal adhesion and leukocyte migration pathways are upregulated in Kir6.1^wt/VM^ mice (Figure 3D), in contrast to the reported downregulation of these pathways in exercise-induced physiological cardiac hypertrophy.^24^^,^^25^ No significant changes in the expression of K_ATP_ channel genes (*KCNJ8, KCNJ11, ABCC8, ABCC9*) were observed.

Cardiac fibrosis is also a common outcome of pathological elevations in RAS signaling^26^ and a mild increase in expression of collagen genes was observed in Kir6.1^wt/VM^ mice (Figure 3E). However, hydroxyproline levels (a correlate of collagen protein expression) in the ventricular wall of Kir6.1^wt/VM^ hearts were not elevated at either 3 or 12 months of age. This suggests that significant collagen deposition does not occur in CS-associated cardiac hypertrophy (Figure 3F).

### Long-Term Consequences of High-Output Hypertrophy

**Figure 4.**
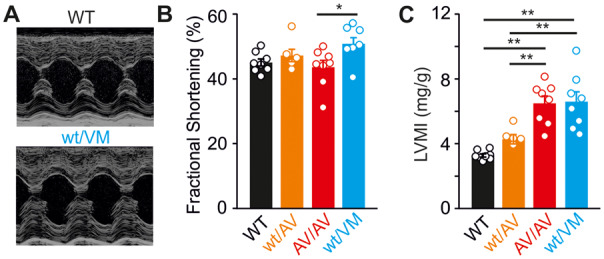
High-Output Hypertrophy Is Maintained with Aging in Cantú Mice. (**A**) Representative M-mode echocardiograms from 12-month-old WT and Kir6.1^wt/VM^ mice. Fractional shortening is maintained in 12-month-old Cantú mice (**B**) despite marked cardiac hypertrophy (**C**). WT mice from Kir6.1^wt/VM^ and SUR2^wt/AV^/SUR2^AV/AV^ lines were essentially identical and were combined for analysis. For all figures, individual data points are represented as open circles, bars show mean ± SEM. Statistical significance was determined by ANOVA and post hoc Tukey’s test. *P < 0.05, **P < 0.01.

**Figure 5.**
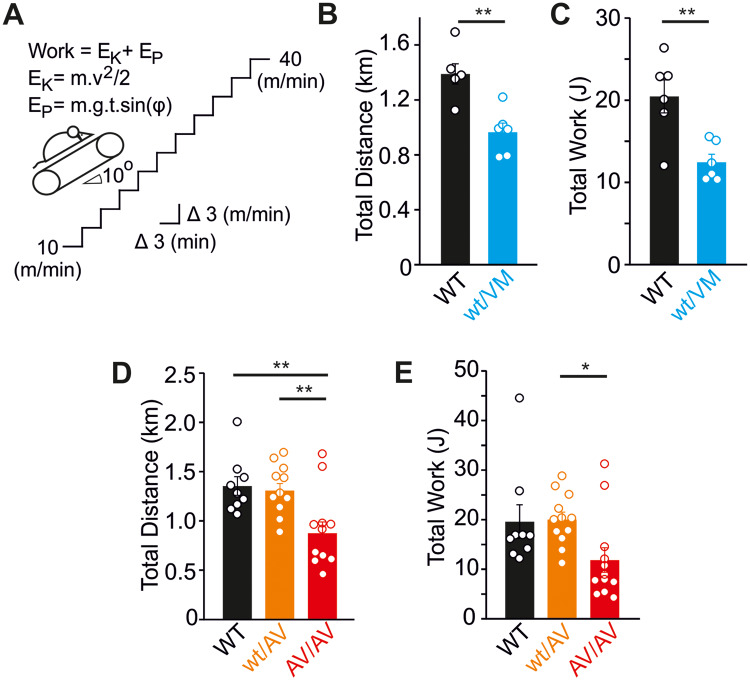
Exercise Tolerance Is Impaired in Cantú Mice. (**A**) Schematic showing the treadmill tolerance test protocol. Total work was calculated according to the equations shown, as described in Methods. Kir6.1^wt/VM^ mice covered significantly less distance (**B**) and tolerated significantly reduced workloads (**C**). (**D**, **E**) Treadmill tolerance test data from WT, SUR2^wt/AV^, and SUR2^AV/AV^ mice. For all figures, individual data points are represented as open circles, bars show mean ± SEM. Statistical significance was determined by Student’s *t*-test (B, C) or ANOVA and post hoc Tukey’s test (D, E). *P < 0.05, **P < 0.01.

The long-term consequences of high-output cardiac hypertrophy remain unclear. In pathological cardiac hypertrophy arising from multiple other etiologies, there is a progressive decline in function as the initially adaptive cardiac remodeling ultimately “decompensates,” but whether there is a similar progression in CS is unknown. Echocardiographic analysis at 12 months of age indicates no significant decline in functional properties in Kir6.1^wt/VM^ mice with aging (Table 1), and Kir6.1^wt/VM^, SUR2^wt/AV^, and SUR2^AV/AV^ hearts maintain normal fractional shortening despite persistent LV hypertrophy (Figure 4A–C). This suggests that basal cardiac pump function is maintained in Cantú mice as they age. However, this compensatory high-output state may be expected to limit cardiac reserve, and hence to limit exercise capacity. To examine this possibility, we subjected Cantú mice (at 5 months of age) to a treadmill exercise stress test. This test consisted of running mice to exhaustion using a progressively increasing workload protocol (Figure 5A). As shown in Figure 5B and C, Kir6.1^wt/VM^ mice exhibited marked exercise intolerance, covering ∼30% shorter distance and achieving ∼40% reduced workload prior to exhaustion. Exercise intolerance was absent in SUR2^wt/AV^ mice, consistent with the milder molecular phenotype of the SUR2(A478V) mutation, but performance was clearly reduced in SUR2^AV/AV^ mice (Figure 5D and E). While systematic norms for the limited pool of available patients may be difficult to achieve, this dramatically reduced reserve in more severely affected Cantú mice warrants studies of exercise tolerance in humans with CS.

## Discussion

### The Mechanism of High-Output Cardiac Hypertrophy Driven by Vascular Hypo-Excitability in CS

The above results demonstrate that chronic vasodilation resulting from K_ATP_ GoF triggers upregulation of RAS, which drives cardiac hypertrophy in CS. Cardiac hypertrophy in CS is clearly distinct from other well-described conditions such as dilated and hypertrophic cardiomyopathies (DCM and HCM) in its structural and functional characteristics as well as its etiological basis. In contrast to models of DCM and HCM, there is no obvious age-dependent decrease in fractional shortening in Cantú mice by 12 months (approximately equivalent to middle age in humans), and cardiac hypertrophy in CS does not include significant fibrosis. Furthermore, unlike heart failure with preserved ejection fraction, which is often associated with cardiac hypertrophy, there is no obvious diastolic dysfunction in Cantú mice. In exhibiting cardiac hypertrophy alongside elevated stroke volume, the Cantú heart is reminiscent of exercise-induced hypertrophy (Athlete’s heart). However, transcriptional analyses reveal strikingly different underlying changes, including upregulation of focal adhesion and leukocyte migration pathway genes in Kir6.1^wt/VM^ mice, which are downregulated in exercise-trained mice,^25^ as well as a moderate upregulation of distinct transcriptional markers of pathological remodeling such as *Acta1*, *Nppa*, and *Nppb* in Cantú mice.^24^^,^^25^^,^^27^^,^^28^ Furthermore, treadmill tests reveal a striking decrease in tolerated workload in Kir6.1^wt/VM^ mice, indicative of maladaptive remodeling and decreased cardiac reserve in CS.

Here we show that, in CS, high-output cardiac hypertrophy arises from decreased VSM excitability and the consequently lowered SVR, which in turn triggers compensatory increase in cardiac mass and output, and blood volume, thereby serving to partially normalize blood pressures and tissue perfusion. Blockade of RAS with the ACE-I captopril or the ARB losartan can reverse this cardiac remodeling, but this represents an effective *decompensation* of the heart, i.e. reversal of a presumably compensatory change. Due to the current lack of awareness of CS, some patients have been misdiagnosed with HCM or DCM and, as a result, have been administered conventional therapies, including ACE-Is/ARBs or other vasodilatory agents such as diuretics, β-blockers, or sildenafil.^3^ Treatment with these agents may then evoke seemingly appropriate changes in understood symptomatic markers of HCM or DCM, for example, reversal of edema, cardiac hypertrophy, or PH, but they will exacerbate the underlying primary decrease in SVR, and reduce tissue perfusion, and thus are likely to be inappropriate treatments for CS patients. Instead, a therapy that addresses the underlying smooth muscle pathology would be appropriate. We recently demonstrated that the K_ATP_ channel inhibitor glibenclamide reverses both primary decreases in SVR and secondary cardiac remodeling in SUR2^wt/AV^ mice,^20^ and thus is a potential approach to target the underlying molecular defect directly. Future studies should compare the effect of conventional heart failure therapies and K_ATP_ channel inhibitors on the HOHF-like phenotype observed in Cantú mice.

### The Mechanistic Link Between Vascular Dilation and Cardiac Hypertrophy in CS

RAS includes both local tissue-specific signaling pathways and the classical systemic pathway.^29^ Increased RAS is normally a driver of elevated blood pressures due to Ang II-mediated vasoconstriction and aldosterone release, with consequent fluid and salt retention. This hypertensive effect exerts increased afterload on the heart, increasing wall stress and resulting in hypertrophic remodeling. Due to the prevailing vasodilatory effect of smooth muscle K_ATP_ channel GoF, blood pressures remain low in Cantú mice, despite elevated RAS, and thus cardiac hypertrophy likely arises from a different mechanism. Despite further lowering blood pressure, both the ACE-I captopril and the ARB losartan caused dramatic reversal of cardiac hypertrophy. Therefore hypertrophy in CS is not a direct response to lowered blood pressure (BP) alone, but occurs due to the effects of compensatory Ang II production and signaling.

Renal Ang II (AT1) receptors are required for Ang II-evoked, hypertension-associated cardiac hypertrophy,^30^ but locally increased RAS signaling within the heart has also been proposed as a driver of ventricular hypertrophy, even in normotensive mice,^31^^,^^32^ though conflicting results have been found in multiple transgenic mouse models.^33–36^*In vitro*, Ang II has been shown to lead to cellular hypertrophy in cultured cardiomyocytes via the paracrine effect of TGFβ1 release from cardiac fibroblasts.^37^^,^^38^ TGFβ1is a key mediator of Ang II-induced hypertrophy *in vivo*^39^; myocardial *Tgfb1* mRNA expression is upregulated by Ang II *in vitro*^26^^,^^40^; and mRNA and protein levels are elevated in patients with idiopathic hypertrophic obstructive cardiomyopathy.^41^^,^^42^ However, there was no significant change in *Tgfb1* expression in Cantú mice (fold change in Kir6.1^wt/VM^ = 1.14, FDR = 0.31), providing further mechanistic distinction between CS and other hypertrophic conditions. The Cantú mice provide a key tool for future studies aimed at dissecting the detailed mechanism of RAS-induced cardiac hypertrophy in the absence of hypertension.

### Long-Term Consequences of High-Output Cardiac Hypertrophy in CS

Elevated RAS causes salt and fluid retention, which might contribute to the edema and PH observed in CS patients. In addition to edema and PH, decreased exercise tolerance, low systemic vascular resistance, and elevated cardiac output are also observed in both CS and HOHF.^43^^,^^44^ HOHF is also associated with reduced SVR, which can result from peripheral vasodilation in response to anemia, sepsis, thyrotoxicosis, thiamine deficiency (Beriberi), or obesity. It can also be a consequence of arteriovenous shunting, either naturally occurring or iatrogenic, as in AV fistulae introduced for renal dialysis.^43–45^ Both peripheral vasodilation and AV shunts are observed in CS,^3^^,^^4^^,^^46^ and thus we suggest that increased vascular K^+^ channel activity may be an unrecognized basis for HOHF. Heart failure with *increased* cardiac output appears oxymoronic and what exactly is “failing” in HOHF remains poorly defined. In the only large-scale analysis of HOHF patients of various etiologies, both high output and mortality were strongly correlated with the severity of decreased SVR.^44^ Whether mortality in HOHF is due to CV or hemodynamic abnormalities, or to the myriad pathologies ascribed as the underlying causes of HOHF in the above study, is not really clear. In the case of CS, the long-term consequences of the high output cardiac state are unknown, and we have not detected any obvious age-dependent decline of cardiac function in Cantú mice. Whether cardiac remodeling in either CS or HOHF ultimately limits subsequent adaptations to demand, or predisposes the heart to worsened maladaptive remodeling in the face of secondary stresses,^43^ also remains unknown. CS patients thus represent a key population for the future study of the long-term consequences of decreased SVR and potentially of HOHF.

In conclusion, we demonstrate that CS-associated K_ATP_ channel GoF provokes low SVR, which triggers elevated RAS and significant structural and transcriptional remodeling in the heart. We propose that the constellation of features observed in CS represents a genetically and mechanistically defined example of high-output cardiac hypertrophy with the potential to develop into HOHF.

## Methods

### Mouse Models of CS and Study Approval

Cantú mice were generated previously using CRISPR/Cas9 genome editing.^19^ The *KCNJ8* (c.193G>A/195A>G) and the *ABCC9* (c.1427C>T) mutations were introduced to mimic the human disease-causing amino acid substitutions, Kir6.1(V65M) and SUR2(A487V), respectively. Heterozygous Kir6.1(V65M) (Kir6.1^wt/VM^), and SUR2(A478V) (SUR2^wt/AV^) mice mimic the autosomal-dominant context observed in patients. SUR2^wt/AV^ mice were also in-crossed to generate viable homozygous mutant mice (SUR2^AV/AV^) that were also included in analysis. Homozygous Kir6.1 mice (Kir6.1^VM/VM^) die shortly after weaning^19^ and thus were not used in this study. Male and female mice were used for all experimental groups. Littermate WT mice were used for all comparisons.

All studies were performed in compliance with the standards for the care and use of animal subjects defined in the NIH Guide for the Care and Use of Laboratory Animals and were reviewed and approved by the Washington University Institutional Animal Care and Use Committee.

### Heart Weight Measurements

Hearts were rapidly dissected from mice following overdose anesthesia with 2.5% avertin (tribromoethanol; Sigma). Hearts were rinsed in 0.9% NaCl, blotted, and weighed. Heart weight was normalized to tibia length (HW/TL; mg/mm). Captopril (400 mg/L; Sigma) and losartan (90 mg/mL; Santa Cruz Biotechnology) were administered in drinking water for 4 weeks (water was changed every 3 days).

### Blood Pressure Measurement

BP was recorded from adult mice using a Millar pressure transducer (model SPR-671) inserted into the aorta via left carotid artery cauterization in anesthetized mice (1.5% isoflurane), as previously described.^19^ Data were acquired using the PowerLab data acquisition system (ADInstruments), and mean arterial pressure was analyzed using LabChart 7 (ADInstruments). For measurement of systemic vascular resistance, MAP was divided by cardiac output (calculated as the product of the stroke volume from echocardiographic measurements and the heart rate measured in BP recordings from the same mouse).

### Assessment of RAS Activity

Sera were collected by exsanguination of anesthetized mice and ELISA used to measure plasma renin activity (Thermo Fisher; EMREN1) and plasma Ang II (LSBio; LS-F523) levels according to the kit manufacturer’s instructions.

### Blood Volume Measurement

Kir6.1^wt/VM^ and WT littermate mice (4–5 months of age) were anesthetized using isoflurane and the femoral artery and jugular vein were cannulated. Initial blood sampling (50 μL) was taken from the arterial cannula prior to injection of a 50 μL volume of 1.2 mg/mL TxR-A (Thermo Fisher; A23017) dissolved in sterile saline (0.9% NaCl) via the jugular cannula. A 50 μL blood sampling (via the femoral artery cannula) was then repeated 3, 6, 12, and 18 min after TxR-A injection, with each sampling event immediately followed by a 50 μL injection of sterile saline via the jugular cannula. After clotting, samples were centrifuged and sera fluorescence was measured (590 nm excitation/615 nm emission) using a Safire^10^ plate reader (Tecan). Fluorescence values at each time point were plotted and a linear fit of the data points was back extrapolated to yield an estimate of the fluorescence at time zero (Figure 3A). TxR-A concentration at time zero (C*_t_*_= 0_) was estimated by comparing the fluorescence value at time zero with a standard curve (TxR-A dissolved in saline), allowing for calculation of the TxR-A dilution factor and plasma volume according to eqn (1):

(1) $$\text{Plasma}\;\text{Volume}\;\left(\text{in}\;\text{mL}\right)=(C_{\text{inj}}\times V_{\text{inj})})/C_{t}{}_{=0}$$

where C_inj_ is the concentration of TxR-A injected (1.2 mg/mL), V_inj_ is the volume of TxR-A solution injected (0.05 mL), and C*_t_*_= 0_ is the estimated concentration of TxR-A at time zero. Plasma volume was normalized to both body weight and tibia length for each mouse.

### RVSP and Lung Weight Measurement

Right ventricular pressure measurements were recorded from adult mice. Briefly, mice were anesthetized with isoflurane (1.5% maintenance) and then intubated and ventilated at 200–400 μL. Following establishment of ventilation, pancuronium (1 mg/kg IP) was given. This anesthesia combination produces a near physiologic heart rate of 500 beats/min, while still allowing for a surgical plane of anesthesia without breathing artifacts. The right external jugular vein was then identified and cannulated with a 1.2 French high fidelity micromanometer pressure catheter (Transonic-Scisense Advantage System, London, ON, Canada). The catheter was advanced into the right atrium through the tricuspid valve into the right ventricle to assess RV pressures. Continuous RV systolic and diastolic pressures and heart rate were recorded and analyzed with commercially available Transonic software using the LabScribe system.

Lungs were dissected from euthanized mice, rinsed in saline, blotted and weighed for wet weight, before being dried in an oven at 60°C for 72 h and weighed again for dry weight.

### RNASeq

Total RNA from dissected cardiac apices was enriched for polyA-tailed mRNA and mRNA fragmentation. Starting tissue samples, therefore, inevitably contain a mix of cell types. First- and second-strand cDNA synthesis and addition of indexed adapters were carried out as previously described.^47^Single-end, 50 nt reads were obtained on an Illumina HiSeq 3000 instrument at Washington University’s Genome Technology Access Center. Mapping of sequencing reads to the mouse transcriptome (Ensembl GRCm38) was performed using TopHat 2.1.1, with Bowtie2 2.3.1 as the alignment engine. TopHat was employed to only consider exon–exon junctions in the supplied gtf and to allow a maximum of one mismatched nucleotide. The mean number of transcriptome-aligned reads per heart was 27 million. Read quantitation on a per gene basis was performed with HTSeq.^48^ Detectable RNAs were assessed as those present at or above 1 read per million in at least three of five hearts from either genotype group, resulting in 13 190 RNAs for downstream analyses. Calculation of fold change and FDR for differentially expressed RNAs used the R/Bioconductor packages limma and voom.^49^ For clarity of presentation and to enable comparison with other studies, individual gene expression levels in column graphs are rendered as Fragments Per Kilobase of exon per Million reads mapped to the transcriptome (FPKM), calculated from TopHat-aligned read counts using Cufflinks 2.1.1 ± SEM.^50^ KEGG pathway analysis was performed using DAVID 6.8 (https://david.ncifcrf.gov/).^51^ Genes exhibiting a fold change either <−1.2 or >1.2 and with an FDR < 0.1 were included for downregulated and upregulated pathways, respectively. RNASeq data were deposited in GEO (GSE110592; https://www.ncbi.nlm.nih.gov/geo/query/acc.cgi?acc=GSE110592).

### Fibrosis

For quantification of myocardial collagen content, the combined hydroxyproline levels of the right and left ventricular free walls were quantified as previously reported.^52^ Hydroxyproline content was determined using a chloramine-T colorimetric assay^53^ imaged using a Synergy H4 Multi-Mode plate reader, where hydroxyproline concentrations were determined by comparison to a standard curve of hydroxyproline (Sigma). Hydroxyproline content was normalized to the dry weight of each sample following hydrolysis and drying in a SpeedVac.

### Echocardiography

Echocardiographic measurements were made using the Vevo 2100 Imaging System (VisualSonics), equipped with a 30-MHz linear-array transducer, as previously described.^19^^,^^54^^,^^55^ Mice were lightly anesthetized using 100 mg/kg i.p. Avertin (tribromoethanol; Sigma).

### Exercise Tolerance Test

Exercise tolerance was determined using an involuntary treadmill running test. Mice (5 months of age) were habituated to the treadmill apparatus (Columbus Instruments Model Exer3/6 Treadmill), running at a constant speed of 10 m/min, at an incline of 10°, for 10 min for 3 consecutive days. On the following day, mice were subjected to an exercise tolerance test in which the belt speed was initially set at 10 m/min and increased by 3 m/min every 3 min (Figure 4D), and mice were run until exhaustion. Exhaustion was defined as a mouse remaining on the shock grid for five continuous seconds. During habituation and testing, the voltage shock was set at 0.5 mA (2 Hz). The total distance (in meter) covered by each mouse was calculated alongside the total tolerated workload (in Joules) performed. Workload was calculated as the sum of the kinetic (E_K_) and potential (E_P_) energy as previously described^56^ and according to eqns (2)–(4):

(2) $$\text{Work}={\;E}_{K}+{\;E}_{P}$$

(3) $$E_{K}=mv^{2}/2$$

(4) EP=m.g.t. sin (φ)

where *m* is the mouse mass (in kilogram), *v* is the belt velocity (in meters per second), *g* is acceleration due to gravity (taken as 9.81 m/s^2^), *t* is duration of exercise (in seconds), and φ is the angle of incline of the treadmill.

### Data Analysis

See RNASeq section for statistical analysis of transcriptional data. All other statistical analyses were performed using Microsoft Excel with the Real Statistics Resource Pack add-in (www.real-statistics.com). For comparisons of multiple experimental groups, one-way ANOVA was used followed by post hoc Tukey’s tests for pairwise comparisons. Student’s *t*-tests were used for comparisons of two groups, with Bonferroni correction used when making multiple comparisons. All values are expressed as mean ± SEM.

## Funding 

This work was supported by National Institutes of Health grant R35 HL140024 (to C.G.N.). C.M. was supported by American Heart Association Postdoctoral Fellowship 19POST34380407. 

## Conflict of interest statement

None declared.

## Authors’ Contributions

C.Mc., Y.H., and C.G.N. designed research studies; C.Mc., Y.H., S.J.M., R.P., T.J.B., and A.K. conducted experiments and acquired and analyzed data; C.Mc. and C.G.N. wrote the manuscript. All authors critically reviewed the manuscript.

## Supplementary Material

zqaa004_Supplementary_Data
