# Supplementary material for: The Mechanism of High-Output Cardiac Hypertrophy Arising From Potassium Channel Gain-of-Function in Cantú Syndrome
Source: Function (Oxf). 2020 Jun 18;1(1):zqaa004. doi: 10.1093/function/zqaa004 (PMC7446247; doi:10.1093/function/zqaa004)
Supplement: zqaa004_Supplementary_Data [file zqaa004_supplementary_data.pdf]

List 1: List of genes associated with specific pathways identified by KEGG Pathway analysis of all genes which were significantly downregulated (<-1.2 fold; FDR < 0.1) in Kir6.1$^{wt/VM}$ hearts, as determined from RNASeq analysis.

Oxidative phosphorylation (KEGG Pathway mmu00190):

ATP5D, ATP5E, UQCRC1, ATP5B, CYC1, COX5A, UQCRFS1, COX5B, UQCRQ, NDUFS7, NDUFS6, UQCR10, NDUFS5, UQCR11, NDUFS8, ATP5L, ATP5O, NDUFS3, NDUFS2, ATP5H, ATP5K, NDUFS1, ATP5J, NDUFB11, NDUFB10, NDUFC2, COX4I1, NDUFA13, NDUFC1, NDUFA10, NDUFA12, COX6C, UQCRH, ATP5C1, NDUFB4, NDUFB6, NDUFB7, NDUFB8, NDUFB9, COX7B, COX7C, ATP5G2, ATP5G3, COX6B1, NDUFA4, NDUFA5, ATP5J2, NDUFA2, NDUFA3, COX7A2, NDUFA8, COX7A1, COX8B, NDUFA9, NDUFA6, COX8A, NDUFA7, ATP5F1, NDUFA1, NDUFV3, SDHA, SDHB, NDUFV1, SDHC, NDUFV2, COX6A2, ATP5A1

Metabolic pathways (mmu01100):

LDHB, ALAD, SGMS1, COX5A, COX5B, CMBL, FDFT1, FAH, GOT2, ACSS1, UQCR10, PGP, IDH3G, GOT1, UQCR11, SPR, CES1D, PDHA1, HADH, GPT2, ALDH6A1, ACAA2, SUCLG1, PIGYL, QDPR, PNPLA2, FBP2, COX6C, PGLS, NME2, NNT, AKR1B10, ATP5C1, PCCB, MDH2, MDH1, XDH, HSD17B10, ME3, ACADSB, COX7B, COX7C, ACAT1, HADHA, HADHB, ISYNA1, MUT, IVD, COX6B1, ALDH4A1, FH1, AMD1, MGAT4B, NADK2, GCDH, ATP5J2, ACY1, COX8B, MAOB, COX8A, MCAT, GALT, EPHX2, IDH3B, ACACB, AK4, IDH3A, NDUFV3, HMGCS2, MLYCD, NDUFV1, MTR, POLD2, NDUFV2, ALDH2, COX6A2, DCXR, DUT, ATP5D, NAMPT, ACOX1, ATP5E, UQCRC1, ATP5B, CYC1, ACOT2, ACOT1, UQCRFS1, UQCRQ, AUH, NDUFS7, MCCC2, NDUFS6, NDUFS5, MCCC1, MCEE, NDUFS8, GSTZ1, ATP5L, ATP5O, SUCLA2, NDUFS3, NDUFS2, ATP5H, AGPAT3, NDUFS1, ATP5K, ATP5J, NDUFB11, ACADM, NDUFB10, PFKL, ACADS, HMBS, NDUFC2, NDUFA13, COX4I1, NDUFC1, ACADL, NDUFA10, COQ7, NDUFA12, NAPRT, ACADVL, COQ2, CKM, DHRS4, DGAT2, PANK1, UQCRH, UROD, PLA2G5, NDUFB4, POLR2F, NDUFB6, BCAT2, NDUFB7, NDUFB8, NDUFB9, ECHS1, ATP5G2, ATP5G3, ACSL1, CKMT2, ENO3, ACAA1A, BCKDHA, NDUFA4, NDUFA5, NDUFA2, NDUFA3, NDUFA8, NDUFA9, NDUFA6, CS, BCKDHB, NDUFA7, AMACR, ATP5F1, NDUFA1, SDHA, ADI1, SDHB, SDHC, GPT, ATP5A1, SMPD2

Parkinson's Disease (mmu05012):

ATP5D, ATP5E, UQCRC1, ATP5B, CYC1, PINK1, COX5A, UQCRFS1, COX5B, UQCRQ, NDUFS7, NDUFS6, UQCR10, NDUFS5, UQCR11, NDUFS8, ATP5O, NDUFS3, NDUFS2, ATP5H, NDUFS1, ATP5J, NDUFB11, NDUFB10, NDUFC2, COX4I1, NDUFA13, NDUFC1, NDUFA10, NDUFA12, COX6C, UQCRH, ATP5C1, NDUFB4, NDUFB6, NDUFB7, NDUFB8, NDUFB9, COX7B, COX7C, ATP5G2, ATP5G3, COX6B1, NDUFA4, NDUFA5, NDUFA2, NDUFA3, COX7A2, NDUFA8, COX7A1, COX8B, NDUFA9, NDUFA6, COX8A, NDUFA7, ATP5F1, NDUFA1, NDUFV3, SDHA, SDHB, NDUFV1, SDHC, NDUFV2, COX6A2, ATP5A1

Alzheimer's disease (mmu05010):
ATP5D, ATP5E, UQCRC1, ATP5B, CYC1, COX5A, UQCRFS1, COX5B, UQCRQ, NDUFS7, NDUFS6, UQCR10, NDUFS5, UQCR11, NDUFS8, ATP5O, NDUFS3, NDUFS2, ATP5H, NDUFS1, ATP5J, NDUFB11, NDUFB10, NDUFC2, COX4I1, NDUFA13, NDUFC1, NDUFA10, NDUFA12, COX6C, UQCRH, ATP5C1, HSD17B10, NDUFB4, NDUFB6, NDUFB7, NDUFB8, NDUFB9, COX7B, COX7C, ATP5G2, ATP5G3, COX6B1, NDUFA4, NDUFA5, NDUFA2, NDUFA3, COX7A2, NDUFA8, COX7A1, COX8B, NDUFA9, NDUFA6, COX8A, NDUFA7, ATP5F1, NDUFA1, NDUFV3, SDHA, SDHB, ATP2A2, NDUFV1, SDHC, NDUFV2, COX6A2, ATP5A1, APBB1

Huntinton's disease (mmu05016):
ATP5D, ATP5E, UQCRC1, ATP5B, CYC1, COX5A, UQCRFS1, COX5B, UQCRQ, NDUFS7, NDUFS6, UQCR10, NDUFS5, UQCR11, NDUFS8, ATP5O, NDUFS3, NDUFS2, ATP5H, NDUFS1, ATP5J, NDUFB11, NDUFB10, NDUFC2, COX4I1, NDUFA13, NDUFC1, NDUFA10, NDUFA12, COX6C, UQCRH, ATP5C1, NDUFB4, POLR2F, NDUFB6, NDUFB7, NDUFB8, NDUFB9, COX7B, COX7C, ATP5G2, ATP5G3, COX6B1, NDUFA4, NDUFA5, NDUFA2, NDUFA3, COX7A2, NDUFA8, COX7A1, COX8B, NDUFA9, NDUFA6, COX8A, NDUFA7, ATP5F1, SOD1, NDUFA1, SOD2, NDUFV3, SDHA, SDHB, NDUFV1, SDHC, NDUFV2, COX6A2, ATP5A1

Non-alcoholic fatty liver disease (NAFLD) (mmu04932):

UQCRC1, CYC1, COX5A, UQCRFS1, COX5B, UQCRQ, NDUFS7, NDUFS6, NDUFS5, UQCR10, UQCR11, NDUFS8, NDUFS3, NDUFS2, NDUFS1, NDUFB11, NDUFB10, PRKAB1, NDUFC2, COX4I1, NDUFA13, NDUFC1, NDUFA10, NDUFA12, COX6C, UQCRH, NDUFB4, NDUFB6, NDUFB7, NDUFB8, NDUFB9, COX7B, COX7C, XBP1, COX6B1, PIK3R1, NDUFA4, SREBF1, NDUFA5, NDUFA2, NDUFA3, COX7A2, NDUFA8, COX7A1, COX8B, NDUFA9, NDUFA6, COX8A, NDUFA7, NDUFA1, NDUFV3, SDHA, SDHB, NDUFV1, SDHC, NDUFV2, COX6A2

Biosynthesis of antibiotics (mmu01130):

HSD17B10, LDHB, BCAT2, ECHS1, ACAT1, HADHA, CMBL, HADHB, FDFT1, GOT2, PGP, ACSS1, ISYNA1, GOT1, IDH3G, ENO3, PDHA1, FH1, SUCLA2, HADH, ACAA1A, BCKDHA, ACAA2, GCDH, ACADM, ACY1, PFKL, SUCLG1, CS, BCKDHB, IDH3B, AK4, FBP2, IDH3A, SDHA, SDHB, NME2, PGLS, HMGCS2, SDHC, ALDH2, PCCB, MDH2, MDH1

Carbon metabolism (mmu01200):

ME3, ECHS1, ACAT1, HADHA, GOT2, PGP, ACSS1, MUT, GOT1, IDH3G, MCEE, ENO3, PDHA1, FH1, SUCLA2, GPT2, ALDH6A1, ACADM, PFKL, ACADS, SUCLG1, CS, IDH3B, FBP2, IDH3A, SDHA, SDHB, PGLS, SDHC, GPT, PCCB, MDH2, MDH1

Valine, leucine and isoleucine degradation (mmu00280):

BCKDHA, ACAA2, ALDH6A1, HSD17B10, ACADSB, ACADM, BCAT2, ACADS, BCKDHB, ECHS1, ACAT1, HADHA, HADHB, AUH, MCCC2, MUT, HMGCS2, IVD, MCCC1, MCEE, ALDH2, ACAA1A, HADH, PCCB

Fatty acid degradation (mmu 00071):

ECI1, ACAA2, CPT1B, ACOX1, GCDH, ECI2, ACADSB, ACADM, CPT2, ACADS, ECHS1, ACADL, ACAT1, HADHA, HADHB, ACADVL, ACSL1, ALDH2, ACAA1A, HADH

Peroxisome (mmu04146):

XDH, ECI2, ACOX1, ECH1, AMACR, EPHX2, PEX6, MPV17, CRAT, SOD1, PHYH, SOD2, PEX7, ACSL1, DHRS4, MLYCD, GSTK1, PXMP2, ABCD3, ACAA1A

Citrate cycle (TCA cycle) (mmu00020):

SUCLG1, CS, IDH3B, IDH3A, SDHA, SDHB, IDH3G, SDHC, FH1, PDHA1, SUCLA2, MDH2, MDH1

2-oxocarboxylic acid metabolism (mmu01210):

GOT2, BCAT2, GOT1, IDH3G, ACY1, CS, IDH3B, GPT, GPT2, IDH3A

Pyruvate metabolism (mmu00620):

LDHB, ME3, ACSS1, LDHD, ALDH2, ACACB, PDHA1, FH1, ACAT1, MDH2, MDH1

Ribosome (mmu03010):

MRPS15, RPL13, RPL35, RPL36, RPLP2, RPL37, MRPS21, RPL22L1, RPS2, RPL29, RPS28, RPS29, RPS16, MRPL14, RPL9, RPL3L, RPS21, UBA52, MRPL34

List 2: List of genes associated with specific pathways identified by KEGG Pathway analysis of all genes which were significantly upregulated (>1.2 fold; FDR < 0.1) in Kir6.1$^{wt/VM}$ hearts, as determined from RNASeq analysis.

Leukocyte transendothelial migration (mmu04670):

ACTN4, PECAM1, CTNND1, MSN, CXCL12, VASP, PTPN11, MYL9

Focal adhesion (mmu04510):

ITGA9, COL4A2, CCND1, COL4A1, ACTN4, COL3A1, FLNC, VASP, FLNA, MYL9

List 3: All genes which exhibit fold change >1.2 or <-1.2 (and FDR < 0.1) in Kir6.1$^{wt/VM}$ hearts, from RNASeq analysis.

**Gene**	**WT Expression (FPKM)**		**V65M Expression (FPKM)**		Fold Change	Adj. P Val (FDR)
	Mean	SEM	Mean	SEM		
0610009O20Rik	51.8	1.77	42.79	1.06	-1.21	0.01357
0610040J01Rik	1.8	0.13	1.14	0.14	-1.78	0.01021
1010001N08Rik	6.5	0.30	5.13	0.25	-1.30	0.04791
1110001J03Rik	105.6	2.56	74.95	4.36	-1.42	0.00118
1110002E22Rik	12.7	1.21	15.73	0.50	1.26	0.08259
1110008F13Rik	11.2	0.38	8.38	0.61	-1.35	0.02252
1110065P20Rik	10.1	0.77	6.70	0.33	-1.51	0.02311
1500009L16Rik	1.6	0.19	3.37	0.35	2.16	0.00397
1700008J07Rik	0.6	0.06	0.38	0.04	-1.61	0.05476
1700037H04Rik	6.5	0.35	8.44	0.54	1.28	0.04199
1700040L02Rik	9.4	0.92	6.94	0.77	-1.38	0.06864
1700084E18Rik	0.9	0.12	0.50	0.04	-1.61	0.09418
1700112E06Rik	1.4	0.19	2.58	0.28	1.72	0.06548
1810011H11Rik	0.4	0.11	1.45	0.15	4.02	0.00142
1810014B01Rik	4.3	0.25	3.24	0.22	-1.33	0.05605

1810026B05Rik	0.3	0.05	0.42	0.02	-1.27	0.04110
2010107E04Rik	608.7	31.96	473.77	21.34	-1.28	0.01604
2010111I01Rik	8.6	0.46	11.31	0.53	1.32	0.01037
2200002D01Rik	8.5	1.22	15.22	2.56	1.74	0.08096
2210408F21Rik	43.1	2.22	33.79	0.95	-1.25	0.01851
2310007L24Rik	3.3	0.24	1.61	0.10	-2.09	0.00097
2310015D24Rik	0.5	0.04	0.80	0.09	1.64	0.04840
2310036O22Rik	32.4	0.98	24.10	1.26	-1.34	0.00465
2310040G24Rik	12.1	0.45	9.12	0.65	-1.35	0.01945
2310061I04Rik	64.1	1.46	52.57	1.67	-1.22	0.00989
2410022M11Rik	0.8	0.07	0.58	0.05	-1.41	0.06307
2610035D17Rik	2.3	0.25	1.50	0.14	-1.59	0.03104
2700078F05Rik	0.3	0.04	0.57	0.07	2.28	0.00869
2700081O15Rik	9.8	0.49	8.02	0.25	-1.23	0.02766
2700094K13Rik	99.6	4.47	76.50	5.93	-1.32	0.03591
2700097O09Rik	4.6	0.13	3.42	0.27	-1.34	0.02407
2810428I15Rik	55.0	1.68	41.67	2.07	-1.32	0.00442
2900026A02Rik	4.9	0.39	6.08	0.31	1.25	0.08390
3110057O12Rik	13.2	0.65	9.86	1.03	-1.36	0.03727
3425401B19Rik	26.7	1.43	38.56	2.47	1.44	0.00279
3632451O06Rik	2.1	0.18	1.41	0.20	-1.49	0.07028
4430402I18Rik	11.6	0.97	7.92	0.61	-1.51	0.01318
4631405J19Rik	3.9	0.39	2.67	0.19	-1.39	0.05217
4930481A15Rik	10.8	1.49	6.53	0.37	-1.63	0.00854
4930513N10Rik	0.6	0.04	0.40	0.03	-1.47	0.06468
4930516B21Rik	1.1	0.08	0.65	0.04	-1.65	0.02219
4932441J04Rik	0.8	0.06	0.62	0.04	-1.34	0.08345
4933431E20Rik	2.6	0.17	3.40	0.19	1.31	0.07028
5031414D18Rik	0.3	0.04	0.65	0.05	1.90	0.00649
5430416O09Rik	1.4	0.05	1.13	0.07	-1.26	0.05732
5730409E04Rik	4.1	0.25	5.48	0.30	1.34	0.01865
5830418P13Rik	0.5	0.17	2.44	0.60	5.54	0.02114
6030408B16Rik	1.7	0.21	2.52	0.19	1.52	0.04290
6330403A02Rik	0.8	0.06	0.54	0.04	-1.51	0.01255
6430548M08Ri	4.3	0.12	6.26	0.51	1.45	0.00487
6430562O15Rik	1.9	0.24	1.22	0.09	-1.50	0.07221
9030407P20Rik	0.3	0.04	0.50	0.03	1.56	0.05092
9030617O03Rik	19.3	0.62	12.51	0.88	-1.56	0.00097
9330158H04Rik	31.7	1.10	23.14	2.22	-1.38	0.02761
9430062P05Rik	1.4	0.18	0.96	0.06	-1.49	0.05452
9530026P05Rik	2.6	0.26	1.61	0.13	-1.61	0.02279
A230004M16Rik	4.2	0.38	3.13	0.31	-1.33	0.07604
A330023F24Rik	2.3	0.16	1.69	0.12	-1.36	0.03442
A530016L24Rik	12.9	0.93	7.84	0.65	-1.68	0.00183
A630001G21Rik	1.2	0.19	1.82	0.12	1.53	0.08657
A930018M24Rik	3.8	0.39	2.44	0.22	-1.56	0.02176

Aacs	2.6	0.29	3.62	0.27	1.37	0.09964
Aaed1	15.3	0.80	12.39	0.25	-1.24	0.02601
Aasdh	8.9	0.47	7.25	0.41	-1.24	0.05893
AB124611	0.5	0.08	0.82	0.07	1.70	0.06039
Abat	3.6	0.15	5.33	0.76	1.40	0.06430
Abca12	0.6	0.06	0.38	0.02	-1.72	0.00265
Abca4	1.1	0.05	0.43	0.07	-2.73	0.00107
Abca8a	39.3	2.56	56.83	2.60	1.45	0.00266
Abca8b	1.7	0.09	3.05	0.21	1.82	0.00033
Abca9	1.7	0.06	2.60	0.17	1.55	0.00178
Abcb4	6.1	0.14	7.66	0.31	1.26	0.00726
Abcb8	27.4	0.75	22.74	0.83	-1.21	0.01755
Abcd3	18.4	0.42	15.20	0.67	-1.21	0.02065
Abcg1	1.1	0.05	2.10	0.12	1.82	0.00032
Abcg2	13.8	0.92	18.79	1.07	1.38	0.01146
Abhd17a	19.0	0.68	15.17	1.09	-1.27	0.03748
Abhd2	4.2	0.31	5.23	0.24	1.26	0.05583
Abhd8	7.8	0.70	6.01	0.37	-1.31	0.05334
Abi3bp	1.2	0.12	1.69	0.11	1.29	0.09888
Abl2	2.3	0.12	2.83	0.12	1.21	0.05097
Ablim2	14.8	0.66	11.85	0.75	-1.25	0.03717
Ablim3	12.6	0.61	7.86	0.52	-1.62	0.00039
Abra	16.1	1.55	23.99	3.36	1.47	0.06830
Acaa1a	27.0	0.94	19.75	0.56	-1.38	0.00084
Acaa2	358.4	18.71	217.45	8.77	-1.65	0.00014
Acacb	50.0	2.76	41.13	2.29	-1.22	0.07613
Acad10	6.0	0.30	4.55	0.25	-1.34	0.02937
Acad12	18.8	0.68	15.20	0.62	-1.24	0.01390
Acadl	352.1	19.82	261.77	3.50	-1.34	0.00209
Acadm	706.3	33.52	497.20	23.05	-1.42	0.00118
Acads	97.5	3.50	75.73	1.42	-1.29	0.00206
Acadsb	58.3	1.32	47.45	1.29	-1.23	0.00582
Acadvl	297.4	15.99	200.40	4.49	-1.48	0.00048
Acat1	180.2	8.68	148.89	4.75	-1.21	0.02928
Acbd6	27.0	0.21	20.25	1.00	-1.33	0.00269
Ace	12.5	0.77	24.93	1.40	1.98	0.00005
Ackr4	3.0	0.29	5.57	0.60	1.79	0.00701
Acot1	13.4	2.14	3.77	0.21	-3.45	0.00017
Acot2	20.7	1.74	13.88	0.68	-1.46	0.00556
Acot7	37.3	1.49	28.39	0.72	-1.31	0.00252
Acox1	67.2	4.37	55.69	1.93	-1.20	0.07340
Acp6	13.4	0.84	11.02	0.51	-1.23	0.05784
Acsl1	268.6	11.77	220.86	10.19	-1.22	0.03322
Acss1	135.4	6.08	107.11	5.72	-1.27	0.01956
Acta1	5619.9	1250.29	35854.81	8218.05	6.17	0.00022
Actn1	6.6	0.33	7.99	0.21	1.21	0.03085

Actn4	42.3	1.07	64.19	1.92	1.51	0.00008
Actr3b	18.3	0.64	13.80	0.67	-1.33	0.00402
Acvr2b	0.8	0.10	0.52	0.05	-1.54	0.08388
Acvrl1	20.8	0.59	27.53	0.91	1.32	0.00188
Acy1	14.0	0.88	10.36	0.17	-1.36	0.00764
Adam15	44.9	2.25	55.78	3.11	1.24	0.03687
Adam23	0.4	0.03	0.71	0.07	1.81	0.00761
Adam8	1.3	0.18	2.14	0.09	1.66	0.03249
Adamts1	8.7	0.72	10.89	0.44	1.26	0.05285
Adamts10	9.6	0.76	13.16	0.51	1.37	0.00954
Adamts2	5.9	0.18	10.76	0.61	1.81	0.00004
Adamts20	0.0	0.00	0.11	0.02	3.99	0.00169
Adamts5	2.9	0.27	4.03	0.37	1.40	0.05810
Adamts7	9.7	0.78	6.15	0.47	-1.59	0.00393
Adamtsl2	4.3	0.29	17.79	1.89	4.09	0.00001
Adap1	0.6	0.06	1.36	0.17	2.10	0.00701
Adck4	3.2	0.13	4.15	0.23	1.28	0.04900
Adcy6	31.0	2.56	24.49	1.42	-1.26	0.08644
Adcy7	1.8	0.12	3.24	0.21	1.74	0.00069
Adgra2	2.8	0.08	4.30	0.30	1.53	0.00107
Adgre1	1.9	0.18	3.50	0.27	1.74	0.00190
Adh1	8.9	0.79	1.39	0.18	-7.00	0.00002
Adhfe1	43.3	2.40	30.42	2.53	-1.44	0.00758
Adi1	14.6	0.19	11.70	0.24	-1.25	0.00337
Adm	4.4	0.39	7.40	0.45	1.67	0.00222
Adora1	3.1	0.18	2.17	0.17	-1.42	0.01227
Adora2a	3.3	0.30	1.27	0.12	-2.59	0.00028
Adprhl1	267.1	16.86	321.26	13.64	1.21	0.07392
Adra1a	3.5	0.10	2.09	0.39	-1.83	0.01821
Adra1b	12.0	0.44	7.04	0.65	-1.72	0.00057
Adra1d	1.4	0.06	1.11	0.04	-1.29	0.08949
Adrb1	6.0	0.52	4.32	0.32	-1.39	0.02735
Aebp1	5.5	0.42	9.15	0.41	1.65	0.00072
AF251705	0.6	0.08	1.28	0.08	2.08	0.00484
Afap1	4.0	0.34	5.12	0.13	1.32	0.03264
Afap1l2	1.4	0.12	2.21	0.18	1.54	0.01802
Aff3	1.1	0.09	2.28	0.18	2.10	0.00091
Ago3	2.5	0.16	2.99	0.09	1.21	0.05206
Agpat3	76.6	2.91	58.12	2.37	-1.32	0.00347
Agpat4	1.4	0.07	1.81	0.05	1.28	0.05784
Agtpbp1	77.6	2.73	58.85	2.28	-1.32	0.00285
Ahdc1	23.3	0.73	19.24	1.26	-1.22	0.05551
Ahnak2	4.6	0.10	8.24	1.35	1.70	0.02759
AI464131	2.6	0.07	2.03	0.12	-1.29	0.02221
AI480526	15.8	0.42	10.87	0.91	-1.47	0.00612
Aif1	2.2	0.35	4.00	0.22	1.89	0.01362

Ajuba	1.8	0.07	2.68	0.15	1.42	0.00769
Ak4	8.4	0.35	6.00	0.17	-1.40	0.00112
Akap12	7.0	0.33	9.22	0.32	1.33	0.00449
Akap5	0.6	0.08	0.44	0.03	-1.43	0.09967
Akip1	7.7	0.31	10.06	0.28	1.30	0.02477
Akr1b10	26.2	0.76	21.36	0.57	-1.23	0.00896
Akr1b8	2.0	0.10	3.40	0.25	1.65	0.00543
Akr1c19	0.8	0.07	1.38	0.11	1.83	0.00819
Akr7a5	25.6	1.39	19.66	0.58	-1.30	0.00714
Akt3	3.3	0.09	4.53	0.27	1.35	0.00559
Alad	22.5	0.64	17.53	0.45	-1.29	0.00257
Aldh18a1	2.0	0.16	2.83	0.18	1.41	0.01675
Aldh1a1	4.4	0.17	7.92	0.70	1.79	0.00073
Aldh1a2	1.8	0.20	3.61	0.59	1.84	0.01865
Aldh1l1	1.3	0.08	0.53	0.11	-2.60	0.00494
Aldh1l2	1.6	0.22	1.03	0.09	-1.57	0.03362
Aldh2	196.1	11.96	129.08	1.76	-1.51	0.00055
Aldh4a1	26.3	2.20	20.91	1.48	-1.26	0.08773
Aldh5a1	4.8	0.16	3.77	0.27	-1.29	0.02588
Aldh6a1	47.0	1.92	34.80	1.40	-1.35	0.00231
Aldh9a1	12.3	0.64	7.87	0.54	-1.56	0.00091
Aldob	2.3	0.32	0.40	0.05	-5.52	0.00009
Alkbh7	29.1	1.13	24.13	1.09	-1.21	0.03553
Alox12	1.6	0.13	3.26	0.32	1.95	0.00157
Alox5	5.9	0.62	3.62	0.24	-1.63	0.00755
Alox5ap	10.4	0.37	16.70	1.03	1.54	0.00540
Alpk1	0.5	0.07	0.78	0.05	1.51	0.04667
Alx4	0.2	0.08	0.56	0.06	2.60	0.01687
Amacr	7.0	0.22	5.74	0.22	-1.23	0.01822
Amd1	12.8	0.85	10.20	0.57	-1.26	0.05794
Ammecr1l	2.1	0.07	2.54	0.10	1.21	0.05039
Amot	2.9	0.36	5.44	0.30	1.94	0.00109
Amotl1	13.6	0.44	16.74	0.97	1.22	0.03609
Ampd2	6.1	0.45	7.90	0.36	1.28	0.04729
Amy1	4.1	0.25	3.02	0.20	-1.33	0.04839
Anapc13	145.2	6.77	117.18	0.82	-1.23	0.01224
Angpt1	4.2	0.23	2.46	0.29	-1.74	0.00178
Angptl1	1.1	0.07	0.46	0.11	-2.66	0.01323
Angptl4	2.1	0.61	0.53	0.05	-3.03	0.04144
Angptl7	0.7	0.08	1.15	0.10	1.58	0.02843
Ankrd1	522.4	89.55	1351.46	208.20	2.61	0.00218
Ankrd23	201.3	20.59	303.73	39.59	1.50	0.03912
Ankrd24	7.6	0.44	5.95	0.20	-1.30	0.02318
Ankrd29	2.6	0.11	3.56	0.27	1.37	0.04971
Ankrd35	0.3	0.06	0.65	0.12	1.95	0.04621
Ankrd45	1.0	0.16	2.34	0.35	2.53	0.00407

Ankrd63	0.6	0.05	0.25	0.07	-2.57	0.01358
Ankrd9	27.4	1.67	19.37	0.66	-1.41	0.00204
Ano1	3.5	0.16	4.88	0.20	1.40	0.00363
Ano10	16.2	1.24	8.89	0.47	-1.81	0.00036
Ano8	4.3	0.18	3.13	0.12	-1.37	0.00257
Antxr1	1.4	0.05	2.00	0.16	1.40	0.01732
Antxr2	27.1	0.69	21.95	1.44	-1.25	0.02828
Anxa1	14.7	1.05	23.62	1.33	1.62	0.00126
Anxa2	27.4	0.86	44.77	1.60	1.63	0.00004
Anxa3	21.6	1.73	34.55	1.80	1.61	0.00116
Anxa4	9.3	0.40	13.02	0.60	1.39	0.00249
Anxa5	92.5	6.30	124.83	5.82	1.35	0.00975
Anxa8	0.3	0.08	0.51	0.07	2.05	0.05956
Aoah	0.4	0.05	0.66	0.06	1.83	0.01725
Aox1	6.3	0.31	5.03	0.35	-1.27	0.03429
Ap4s1	26.8	0.98	22.17	1.00	-1.21	0.03602
Apaf1	1.1	0.10	1.40	0.06	1.28	0.06034
Apba1	0.8	0.03	1.20	0.05	1.46	0.00227
Apbb1	79.6	4.34	58.86	2.28	-1.35	0.00347
Apbb1ip	1.2	0.09	2.26	0.13	1.87	0.00083
Apcdd1	1.4	0.13	2.99	0.22	2.19	0.00054
Apex2	4.8	0.61	3.12	0.20	-1.51	0.03575
Aplnr	23.1	3.17	15.12	1.62	-1.54	0.03976
Aplp1	1.1	0.07	1.41	0.04	1.33	0.05926
Apobr	0.5	0.07	0.82	0.05	1.68	0.03912
Apod	7.5	0.43	19.10	2.38	2.48	0.00047
Apoe	195.7	12.55	286.04	23.96	1.45	0.00776
Apol10b	1.6	0.24	0.73	0.11	-2.04	0.02343
Aqp1	113.0	3.31	15.64	1.86	-7.47	0.00000
Aqp4	0.6	0.04	0.31	0.07	-2.10	0.03571
Ar	0.5	0.04	0.71	0.04	1.46	0.02198
Arap1	18.7	0.51	24.14	1.67	1.27	0.02225
Arf3	6.3	0.20	7.89	0.36	1.26	0.01626
Arhgap23	5.1	0.16	6.43	0.16	1.25	0.00664
Arhgap26	15.2	0.38	12.31	0.71	-1.24	0.02545
Arhgap9	1.5	0.12	2.86	0.21	1.85	0.00216
Arhgdib	15.3	0.69	24.18	0.59	1.57	0.00016
Arhgef19	21.4	0.93	16.92	0.48	-1.26	0.00948
Arhgef25	5.2	0.32	6.35	0.26	1.22	0.06213
Arhgef26	0.6	0.06	0.32	0.03	-1.88	0.01830
Arhgef28	0.8	0.04	0.93	0.03	1.21	0.09159
Arhgef40	25.6	1.41	32.22	1.27	1.25	0.02473
Arl4d	9.0	0.62	6.54	0.36	-1.35	0.02108
Arl8a	11.3	0.48	14.33	0.75	1.26	0.02184
Arpc1b	44.8	0.93	54.30	0.98	1.21	0.00752
Arrb1	7.0	0.24	9.17	0.34	1.29	0.00476

Arrb2	13.1	0.70	16.45	0.50	1.23	0.03943
Arrdc2	3.3	0.67	1.53	0.16	-2.09	0.01535
Art3	246.3	18.26	194.55	7.45	-1.26	0.03322
Art4	6.8	0.70	5.16	0.40	-1.32	0.09468
Art5	11.8	0.85	8.71	0.56	-1.35	0.02577
As3mt	18.7	0.57	13.33	0.45	-1.40	0.00062
Asah1	12.1	0.55	15.15	0.52	1.25	0.01452
Asap1	12.1	0.84	15.51	0.26	1.28	0.01796
Asap2	3.3	0.06	4.53	0.23	1.37	0.00231
Asb11	66.1	2.72	48.67	1.02	-1.36	0.00094
Asb14	58.2	2.19	44.32	2.10	-1.32	0.00479
Asb15	15.7	0.94	12.08	0.74	-1.30	0.02377
Asb4	1.0	0.06	0.68	0.08	-1.55	0.02753
Asb5	9.8	0.67	7.80	0.40	-1.27	0.05134
Asns	0.6	0.08	0.92	0.10	1.58	0.08575
Aspn	4.5	0.37	8.50	0.57	1.85	0.00055
Astn1	0.5	0.10	0.94	0.09	1.86	0.07142
Astn2	0.2	0.01	0.40	0.05	1.81	0.01224
Atg14	2.4	0.07	2.00	0.06	-1.21	0.03646
Atl3	2.0	0.09	2.53	0.15	1.24	0.05212
Atp10a	0.3	0.03	0.45	0.03	1.42	0.06866
Atp13a3	3.5	0.20	4.40	0.11	1.28	0.01058
Atp1a3	0.1	0.02	0.22	0.04	2.05	0.03703
Atp2a2	2469.3	71.94	1985.39	79.13	-1.25	0.01185
Atp2a3	2.8	0.25	4.38	0.42	1.56	0.01176
Atp5a1	1789.3	77.06	1421.32	12.13	-1.25	0.00627
Atp5b	4466.9	176.26	3447.84	28.62	-1.29	0.00233
Atp5c1	634.9	22.02	483.11	14.94	-1.32	0.00184
Atp5d	737.2	27.47	526.18	14.92	-1.40	0.00053
Atp5e	819.8	40.22	544.61	22.97	-1.50	0.00054
Atp5f1	577.5	18.82	456.98	14.85	-1.27	0.00476
Atp5g2	209.9	5.95	163.18	7.71	-1.29	0.00582
Atp5g3	1079.8	39.23	816.77	17.74	-1.32	0.00124
Atp5h	861.1	38.50	668.24	16.11	-1.29	0.00455
Atp5j	598.1	27.67	475.21	8.38	-1.26	0.00808
Atp5j2	849.6	32.02	651.61	16.88	-1.30	0.00225
Atp5k	1128.2	36.26	860.73	15.64	-1.31	0.00114
Atp5l	797.6	24.76	655.99	18.84	-1.22	0.01196
Atp5o	848.8	21.61	652.35	11.77	-1.30	0.00096
Atp6v1a	4.1	0.23	5.09	0.14	1.24	0.02656
Atp8a2	1.7	0.19	2.56	0.34	1.49	0.06661
Atp8b1	0.7	0.06	1.06	0.02	1.55	0.00238
Atp8b2	7.6	0.34	9.60	0.33	1.26	0.01365
AU020206	10.0	0.65	13.51	0.95	1.36	0.03524
AU021092	4.2	0.25	6.42	0.80	1.45	0.05366
Auh	44.1	0.88	35.29	0.74	-1.25	0.00267

B230354K17Rik	1.2	0.06	1.54	0.10	1.31	0.03133
B2m	205.9	5.41	360.34	36.81	1.72	0.00074
B430212C06Rik	0.9	0.04	0.63	0.06	-1.43	0.06266
B4galnt1	1.1	0.07	1.93	0.22	1.82	0.00632
B4galt4	4.8	0.28	6.10	0.09	1.29	0.01673
B4galt5	6.8	0.36	9.19	0.45	1.37	0.00796
Bace2	1.0	0.06	1.38	0.09	1.34	0.04201
Bag1	75.5	2.49	61.69	2.41	-1.23	0.01607
Baiap3	0.7	0.16	2.03	0.21	3.02	0.00404
Bambi	1.4	0.06	1.89	0.15	1.33	0.02376
Banf2os	1.8	0.19	0.92	0.21	-2.02	0.05732
Batf2	1.0	0.13	1.78	0.24	1.68	0.09964
BB218582	12.0	0.62	8.01	0.65	-1.48	0.01204
Bbs2	6.8	0.69	5.19	0.39	-1.35	0.07612
BC002189	9.0	0.57	6.03	0.55	-1.51	0.01740
BC022687	1.0	0.11	1.60	0.09	1.58	0.03538
Bcat2	57.5	1.67	46.67	0.93	-1.23	0.00598
Bckdha	140.5	4.64	100.17	2.93	-1.40	0.00045
Bckdhb	43.1	2.28	27.67	0.75	-1.55	0.00022
Bcl2	1.3	0.14	1.89	0.21	1.45	0.06129
Bcl2l11	3.1	0.23	2.12	0.15	-1.45	0.01312
Bcl6b	9.2	0.99	5.68	0.36	-1.58	0.01073
Bcl9	9.7	0.49	7.90	0.46	-1.24	0.04940
Bcr	1.7	0.11	2.10	0.09	1.21	0.08322
Bdh1	64.4	7.15	129.51	11.51	2.02	0.00054
Bend5	3.6	0.05	2.59	0.17	-1.38	0.01394
Bgn	79.3	2.13	195.33	16.36	2.43	0.00001
Bicc1	4.5	0.39	7.36	0.35	1.65	0.00115
Bik	4.8	0.32	3.61	0.30	-1.37	0.05518
Bin2	0.8	0.12	1.25	0.13	1.65	0.07282
Blk	1.3	0.23	2.00	0.23	1.66	0.08367
Bloc1s1	119.7	3.51	86.55	7.55	-1.38	0.01490
Bloc1s6	8.6	0.35	10.46	0.49	1.21	0.04244
Bmp2k	1.1	0.05	1.42	0.08	1.32	0.02188
Bmper	0.7	0.10	0.44	0.06	-1.67	0.03995
Bmpr2	4.4	0.41	5.30	0.24	1.23	0.09967
Bmx	0.6	0.07	0.85	0.10	1.54	0.06750
Bnc2	0.8	0.07	1.57	0.19	1.86	0.04777
Bnip3	110.8	6.21	80.74	6.75	-1.38	0.01151
Bri3bp	20.3	1.06	15.34	0.24	-1.32	0.00397
Bsg	801.8	24.82	584.68	24.85	-1.38	0.00107
Btbd11	0.6	0.13	0.31	0.02	-1.90	0.04276
Btbd19	2.0	0.12	2.81	0.28	1.37	0.09382
Btk	0.3	0.03	0.42	0.06	1.67	0.07197
Btnl9	9.2	0.32	15.09	0.43	1.64	0.00003
Bub1b	0.1	0.03	0.20	0.01	1.90	0.05886

Bzw2	55.9	1.28	46.50	1.26	-1.20	0.01184
C130023A14Rik	0.7	0.08	0.91	0.04	1.41	0.06084
C1qa	27.5	2.21	42.62	2.36	1.54	0.00269
C1qb	26.4	2.54	42.76	2.21	1.63	0.00186
C1qc	23.3	1.85	36.67	1.73	1.57	0.00146
C1qtnf4	2.7	0.30	1.12	0.07	-2.39	0.00053
C1qtnf6	3.7	0.18	6.46	0.55	1.73	0.00088
C1qtnf7	2.9	0.08	3.69	0.20	1.24	0.06331
C1ra	3.2	0.17	5.98	0.44	1.79	0.00064
C1s1	9.7	0.19	15.47	0.97	1.55	0.00082
C230066G23Rik	1.2	0.17	1.86	0.24	1.69	0.08243
C3	57.8	1.95	108.87	11.12	1.85	0.00038
C3ar1	1.4	0.09	2.27	0.14	1.63	0.00146
C4b	2.3	0.35	7.45	0.81	3.17	0.00009
C5ar1	1.4	0.11	2.19	0.19	1.46	0.03027
C87436	4.2	0.31	3.37	0.21	-1.27	0.08038
Cables1	1.2	0.09	1.92	0.08	1.56	0.00265
Cacna1s	5.7	0.47	4.13	0.48	-1.45	0.04507
Cacnb1	3.1	0.15	4.56	0.35	1.44	0.00965
Cacnb3	1.3	0.08	2.50	0.15	1.89	0.00049
Cacnb4	0.2	0.02	0.10	0.01	-1.76	0.06240
Cadm3	0.9	0.11	1.63	0.20	1.76	0.02658
Calcoco1	53.8	1.93	43.59	1.40	-1.24	0.01146
Cald1	21.2	1.11	33.26	1.36	1.57	0.00036
Calm2	45.4	1.83	54.85	2.61	1.21	0.04247
Calr	123.5	2.65	97.01	7.10	-1.29	0.02343
Calr3	9.7	0.41	6.54	0.35	-1.47	0.00184
Camk1d	0.3	0.04	0.47	0.06	1.60	0.04005
Camk2a	21.5	0.47	15.76	1.20	-1.38	0.00492
Camkk1	0.7	0.06	1.03	0.02	1.56	0.01525
Camkk2	3.6	0.12	4.79	0.24	1.33	0.00743
Cap1	14.9	0.43	18.82	0.38	1.26	0.00314
Capn5	1.1	0.04	1.47	0.06	1.31	0.03344
Car4	21.7	3.06	6.45	0.99	-3.56	0.00016
Car5b	0.4	0.03	0.64	0.04	1.49	0.06014
Car7	4.3	0.35	2.91	0.31	-1.52	0.02330
Car8	4.1	0.23	1.92	0.10	-2.10	0.00003
Carhsp1	7.7	0.17	9.85	0.53	1.27	0.01677
Carns1	7.8	0.51	6.02	0.40	-1.29	0.04239
Casp12	3.7	0.28	6.70	0.63	1.80	0.00349
Casp4	2.6	0.35	3.52	0.16	1.38	0.09094
Casq1	9.9	1.39	19.02	2.63	1.84	0.01820
Cbr2	24.9	2.74	42.32	2.32	1.69	0.00272
Cbx5	10.3	0.65	12.34	0.39	1.21	0.05732
Ccbl2	12.8	0.39	9.12	0.43	-1.41	0.00137
Ccdc141	53.2	1.71	42.44	2.71	-1.26	0.02845

Ccdc173	0.7	0.05	1.08	0.08	1.60	0.04669
Ccdc50	4.8	0.17	5.95	0.12	1.24	0.00796
Ccdc51	3.1	0.19	2.29	0.17	-1.34	0.05101
Ccdc55	6.8	0.39	8.66	0.65	1.30	0.05586
Ccdc68	1.1	0.10	1.94	0.21	1.74	0.02004
Ccdc71l	1.6	0.09	1.22	0.05	-1.30	0.03256
Ccdc80	17.2	0.52	28.90	1.63	1.67	0.00010
Ccdc84	3.0	0.21	2.05	0.19	-1.49	0.03086
Ccdc85a	3.5	0.31	2.33	0.12	-1.50	0.00458
Ccdc85b	11.8	0.51	7.42	0.81	-1.62	0.00566
Ccdc85c	9.0	0.49	6.70	0.54	-1.35	0.02428
Ccdc88a	1.7	0.18	2.06	0.14	1.30	0.09420
Ccdc88b	0.3	0.01	0.46	0.04	1.55	0.01867
Ccdc88c	2.2	0.23	1.17	0.09	-1.90	0.00131
Ccdc92	0.3	0.06	0.55	0.04	1.64	0.09415
Ccl11	1.7	0.10	0.54	0.06	-3.22	0.00019
Ccl12	5.1	1.07	9.43	1.28	1.99	0.05091
Ccl2	3.8	0.59	6.10	0.34	1.61	0.02394
Ccm2l	14.8	0.90	11.47	0.50	-1.28	0.02700
Ccnd1	13.4	1.14	24.68	1.35	1.84	0.00038
Ccnd2	17.2	1.92	26.28	3.95	1.51	0.06034
Ccni	25.3	1.01	20.59	1.21	-1.23	0.03474
Ccr1	0.3	0.03	0.58	0.09	1.66	0.09669
Ccr5	4.1	0.29	7.93	0.71	1.85	0.00310
Cct4	61.3	1.27	50.34	2.02	-1.22	0.01379
Cd163	1.5	0.17	2.46	0.15	1.58	0.00515
Cd180	0.8	0.20	2.38	0.27	2.94	0.00476
Cd248	4.1	0.23	6.72	0.78	1.61	0.00809
Cd300ld	1.1	0.09	1.77	0.15	1.64	0.00740
Cd302	3.5	0.11	4.79	0.24	1.37	0.01171
Cd34	43.2	0.86	55.12	1.23	1.27	0.00160
Cd36	596.4	42.59	461.55	17.57	-1.29	0.02065
Cd37	1.5	0.12	2.39	0.17	1.63	0.01079
Cd44	1.7	0.08	2.23	0.11	1.35	0.01708
Cd48	1.4	0.17	2.22	0.20	1.53	0.05198
Cd52	4.3	0.24	8.68	0.44	1.91	0.00087
Cd53	1.5	0.07	2.78	0.18	1.79	0.00100
Cd68	7.0	0.69	12.49	0.62	1.76	0.00114
Cd72	9.3	0.67	15.89	1.46	1.64	0.02580
Cd74	64.2	3.23	90.38	13.22	1.36	0.08657
Cd84	1.4	0.06	2.38	0.18	1.66	0.00714
Cdc25b	1.4	0.08	2.04	0.20	1.38	0.04723
Cdc42ep1	18.9	1.08	23.83	0.66	1.26	0.01505
Cdc42ep4	4.5	0.42	5.62	0.22	1.26	0.08626
Cdh23	5.0	0.36	0.95	0.15	-6.09	0.00001
Cdh4	1.0	0.11	2.45	0.29	2.48	0.00137

Cdk14	1.6	0.14	2.15	0.11	1.35	0.02586
Cdk19	2.5	0.07	3.58	0.15	1.41	0.00083
Cdk2ap2	21.5	1.50	17.27	0.45	-1.22	0.06661
Cdk6	1.0	0.02	1.22	0.06	1.21	0.03272
Cdkal1	7.6	0.35	6.22	0.24	-1.22	0.04202
Cdkn1b	8.5	0.46	7.11	0.32	-1.21	0.06429
Cdkn1c	9.7	0.64	4.17	0.20	-2.37	0.00002
Cdon	1.5	0.10	1.90	0.12	1.27	0.06713
Cdr2	2.6	0.15	3.27	0.16	1.29	0.04141
Cdr2l	1.1	0.10	1.56	0.06	1.45	0.00776
Cdv3	27.2	1.42	39.87	3.89	1.44	0.01328
Cebpb	13.0	1.29	6.11	0.81	-2.22	0.00216
Cecr2	3.8	0.24	3.05	0.10	-1.25	0.04276
Celsr2	1.2	0.12	0.90	0.09	-1.38	0.06978
Cenpa	21.4	0.88	17.76	1.09	-1.21	0.07716
Cenpf	4.2	0.40	2.54	0.37	-1.69	0.01979
Cenpv	13.9	0.65	10.46	0.21	-1.32	0.00966
Cep85l	3.1	0.31	2.17	0.15	-1.41	0.03571
Cercam	1.2	0.05	1.89	0.25	1.60	0.04411
Ces1d	64.7	6.60	28.43	1.76	-2.26	0.00018
Ces2e	0.4	0.07	1.09	0.04	2.78	0.00037
Cfap43	4.2	0.30	5.61	0.24	1.32	0.01551
Cfh	20.3	0.92	30.96	1.57	1.52	0.00060
Cflar	14.8	1.17	17.62	0.80	1.21	0.09192
Ch25h	0.5	0.20	1.53	0.19	3.96	0.01740
Chac1	1.5	0.12	0.82	0.14	-1.91	0.00692
Chadl	0.9	0.13	0.57	0.08	-1.61	0.08774
Chaf1a	0.3	0.03	0.43	0.03	1.59	0.03391
Chchd10	757.7	27.55	570.76	24.74	-1.33	0.00282
Chchd2	216.9	7.85	170.92	9.01	-1.27	0.01327
Chchd7	88.4	3.48	60.70	1.24	-1.46	0.00031
Chd6	9.7	0.65	7.53	0.59	-1.29	0.05477
Chrd	0.8	0.06	0.64	0.03	-1.32	0.06768
Chrm2	13.7	0.52	11.11	1.03	-1.25	0.08553
Chrna2	0.5	0.09	0.24	0.04	-2.01	0.04593
Chst11	0.6	0.02	0.85	0.04	1.28	0.03756
Chst12	2.3	0.24	3.48	0.18	1.50	0.01714
Chst4	0.5	0.10	0.25	0.04	-1.98	0.06980
Chst7	1.5	0.13	0.73	0.14	-2.17	0.00826
Ciita	0.3	0.02	0.59	0.11	1.63	0.06552
Cilp	2.5	0.27	14.09	1.77	5.57	0.00002
Cisd1	113.1	2.83	87.35	3.23	-1.30	0.00242
Cish	43.4	4.08	32.43	2.73	-1.34	0.06798
Cit	0.6	0.06	0.88	0.09	1.48	0.06384
Cited2	13.1	0.98	8.82	0.47	-1.49	0.00367
Ckap4	5.8	0.37	7.95	0.51	1.38	0.01614

Ckm	1230.8	36.16	1015.41	27.09	-1.21	0.01196
Ckmt2	741.8	25.98	615.09	22.64	-1.21	0.02499
Clasrp	7.1	0.41	5.45	0.23	-1.30	0.01731
Clca3a1	0.4	0.06	0.62	0.05	1.64	0.06430
Clcn1	5.5	0.89	2.79	0.28	-1.99	0.00851
Clcn5	0.6	0.01	0.85	0.04	1.32	0.05991
Cldn15	3.2	0.47	2.08	0.24	-1.58	0.08193
Cldn5	15.5	3.64	4.61	0.79	-3.12	0.01037
Clec10a	2.4	0.25	3.49	0.15	1.47	0.04268
Clec11a	0.3	0.03	0.65	0.07	1.88	0.01851
Clec12a	0.8	0.07	1.44	0.15	1.62	0.03633
Clec12b	0.2	0.03	0.34	0.03	2.21	0.02804
Clec14a	2.8	0.15	4.69	0.25	1.70	0.00035
Clec18a	0.6	0.05	0.30	0.04	-2.11	0.00765
Clec1a	2.2	0.13	3.07	0.23	1.37	0.01612
Clec1b	0.3	0.03	0.45	0.04	1.57	0.06866
Clec2d	10.4	1.11	14.72	1.02	1.44	0.01360
Clec4a1	2.1	0.11	3.41	0.46	1.63	0.02044
Clec4a3	0.9	0.10	1.35	0.10	1.56	0.05453
Clec4n	0.7	0.07	2.72	0.29	3.45	0.00039
Clec5a	0.4	0.08	0.73	0.08	1.73	0.06087
Clec7a	0.9	0.09	1.76	0.25	1.74	0.05794
Clec9a	1.0	0.04	1.41	0.08	1.42	0.01200
Clic1	19.3	0.66	25.42	1.38	1.30	0.01033
Clic5	32.6	1.66	39.63	2.65	1.21	0.09716
Clip3	6.4	0.53	8.71	0.69	1.36	0.04521
Clmn	0.3	0.02	0.11	0.01	-2.52	0.00017
Clmp	1.4	0.12	2.50	0.28	1.76	0.00546
Clpx	23.2	1.28	18.32	0.51	-1.26	0.01388
Cmah	0.5	0.02	0.75	0.05	1.34	0.03050
Cmbl	25.7	1.17	19.65	1.66	-1.34	0.02123
Cmip	4.5	0.16	5.51	0.29	1.24	0.02916
Cmss1	17.8	0.75	14.66	0.34	-1.21	0.03310
Cngb3	0.5	0.06	0.27	0.04	-1.93	0.03003
Cnksr1	6.6	0.70	12.14	1.00	1.85	0.00177
Cnksr3	5.9	0.52	4.69	0.21	-1.27	0.05875
Cnn2	19.4	0.62	24.41	0.76	1.26	0.00612
Cnn3	22.2	1.01	26.59	0.65	1.21	0.02958
Cnnm2	1.2	0.06	0.94	0.05	-1.29	0.04849
Cnp	2.6	0.19	3.43	0.24	1.30	0.06246
Cntn2	0.9	0.11	0.49	0.07	-1.69	0.06301
Cobl	5.9	0.54	7.37	0.27	1.30	0.07006
Col12a1	0.2	0.02	0.52	0.13	2.43	0.01416
Col14a1	1.6	0.09	4.23	0.55	2.48	0.00057
Col15a1	45.1	2.83	76.57	1.78	1.71	0.00009
Col16a1	3.8	0.30	7.52	0.65	1.92	0.00107

Col18a1	1.6	0.05	2.22	0.12	1.44	0.00231
Col1a1	16.2	1.40	32.79	3.88	1.99	0.00160
Col1a2	16.8	0.64	32.55	2.10	1.92	0.00004
Col3a1	43.0	3.42	105.04	10.63	2.42	0.00008
Col4a1	54.1	4.48	72.96	4.51	1.36	0.02688
Col4a2	51.4	3.54	74.10	4.73	1.44	0.00639
Col4a3	0.6	0.05	0.95	0.05	1.77	0.00216
Col4a4	1.0	0.07	1.54	0.11	1.48	0.01296
Col4a5	5.4	0.19	6.75	0.26	1.24	0.01485
Col5a1	6.7	0.39	9.45	0.47	1.41	0.00325
Col5a2	4.8	0.21	10.06	0.85	2.05	0.00010
Col6a1	17.4	0.82	25.68	1.62	1.47	0.00176
Col6a2	18.2	1.30	25.47	1.41	1.40	0.00878
Col8a1	3.1	0.11	9.23	1.24	2.89	0.00010
Colec11	9.4	0.59	7.20	0.65	-1.32	0.05538
Colec12	2.9	0.06	4.58	0.17	1.57	0.00022
Comp	0.6	0.09	1.29	0.24	2.16	0.04564
Coq2	30.6	0.69	24.16	0.64	-1.27	0.00245
Coq7	50.2	1.83	40.33	1.14	-1.24	0.01050
Coq9	316.9	8.66	253.41	4.54	-1.25	0.00276
Corin	28.4	1.61	20.35	1.26	-1.40	0.00620
Coro1a	4.5	0.43	6.37	0.36	1.38	0.02883
Coro1c	15.2	1.08	18.77	0.85	1.23	0.07043
Coro6	205.2	10.59	166.18	6.57	-1.23	0.02804
Cotl1	5.2	0.51	7.42	0.42	1.41	0.02044
Cox11	4.3	0.28	2.65	0.13	-1.60	0.00437
Cox19	38.4	1.69	46.76	2.85	1.22	0.05981
Cox4i1	2247.6	90.26	-2843.68	202856.58	-1.27	0.00432
Cox5a	714.9	48.31	354.32	21.90	-2.02	0.00008
Cox5b	2554.2	114.33	1838.45	44.02	-1.39	0.00083
Cox6a2	2979.4	75.54	2350.98	114.35	-1.27	0.00730
Cox6b1	1292.6	50.02	1058.73	15.95	-1.22	0.01065
Cox6b2	3.5	0.54	0.80	0.09	-3.94	0.00075
Cox6c	912.0	46.19	693.80	15.99	-1.31	0.00396
Cox7a1	2173.2	119.29	1492.58	53.49	-1.45	0.00096
Cox7a2	356.8	10.87	256.14	3.48	-1.39	0.00026
Cox7b	5989.4	288.01	4574.19	136.32	-1.31	0.00407
Cox7c	1059.6	38.44	763.77	26.45	-1.39	0.00086
Cox8a	204.3	4.03	161.04	6.28	-1.27	0.00413
Cox8b	2256.2	93.92	1739.74	31.24	-1.29	0.00266
Cp	30.6	0.90	41.67	2.13	1.35	0.00257
Cpeb1	7.2	0.59	9.57	0.85	1.32	0.07950
Cpm	2.0	0.15	5.74	0.36	2.80	0.00001
Cpn2	0.4	0.05	0.26	0.04	-1.72	0.06276
Cpne2	1.7	0.06	2.44	0.17	1.41	0.01637
Cpox	7.9	0.35	6.12	0.29	-1.29	0.01061

Cpt1a	8.5	0.25	6.74	0.17	-1.26	0.00385
Cpt1b	521.0	31.35	370.74	13.26	-1.38	0.00425
Cpt1c	1.0	0.05	1.38	0.05	1.39	0.01380
Cpt2	120.6	5.69	91.48	3.59	-1.32	0.00433
Cpxm1	1.4	0.20	2.32	0.32	1.65	0.05351
Cpxm2	12.8	2.08	26.97	3.43	2.20	0.00397
Crat	394.6	17.73	292.80	15.47	-1.35	0.00407
Crebzf	9.2	0.54	7.70	0.20	-1.21	0.06566
Creg1	67.2	3.20	45.58	3.33	-1.49	0.00345
Creld2	8.8	0.86	5.91	0.18	-1.46	0.01648
Crem	6.7	0.57	4.89	0.48	-1.42	0.06354
Crhr2	12.0	0.44	9.88	0.65	-1.22	0.06520
Crip1	105.1	6.24	133.60	6.40	1.27	0.02470
Crip2	914.3	36.54	704.43	29.25	-1.30	0.00520
Crispld1	0.3	0.03	0.63	0.01	2.36	0.00076
Crispld2	18.5	1.00	27.05	18.48	1.46	0.00267
Crlf1	0.1	0.02	0.75	0.13	7.77	0.00129
Crlf2	4.0	0.15	3.23	0.28	-1.25	0.09803
Cryba4	4.8	0.54	3.11	0.25	-1.52	0.05221
Cryz	2.6	0.12	2.10	0.07	-1.26	0.04440
Cs	371.3	15.53	296.09	15.40	-1.26	0.01915
Csf1	23.8	1.67	29.15	0.98	1.23	0.05456
Csf1r	9.1	1.07	14.78	0.74	1.64	0.00788
Csf2ra	4.1	0.09	5.27	0.39	1.27	0.04239
Csf2rb	0.2	0.02	0.70	0.08	2.64	0.00073
Csf3r	0.5	0.04	0.79	0.08	1.53	0.04379
Csnk1g1	2.0	0.07	2.43	0.10	1.20	0.05784
Cspg4	4.1	0.12	6.27	0.46	1.53	0.00098
Cspp1	8.0	0.65	9.64	0.34	1.24	0.08357
Ctf1	3.7	0.28	2.06	0.18	-1.82	0.00228
Ctgf	23.9	1.96	53.93	7.83	2.18	0.00107
Cth	1.3	0.15	0.85	0.07	-1.56	0.05952
Ctla2a	8.6	0.79	15.69	0.77	1.85	0.00032
Ctla4	2.6	0.27	1.71	0.19	-1.46	0.09892
Ctnnal1	5.2	0.31	3.95	0.23	-1.32	0.02498
Ctnnd1	18.8	0.81	22.63	0.48	1.22	0.03069
Ctsf	16.5	0.71	13.47	0.37	-1.23	0.01747
Ctsk	1.5	0.06	2.37	0.04	1.55	0.00206
Ctss	5.2	0.44	9.32	0.76	1.75	0.00107
Ctsz	10.6	0.29	16.33	1.22	1.51	0.00347
Cx3cl1	3.7	0.36	5.53	0.21	1.56	0.00540
Cxcl12	29.0	1.59	88.61	4.59	3.05	0.00000
Cxcl14	7.6	0.66	3.71	0.86	-2.31	0.00611
Cxcl16	5.2	0.30	7.08	0.60	1.34	0.04110
Cxcl9	3.1	0.31	0.79	0.15	-3.72	0.00025
Cxcr4	4.3	0.35	2.15	0.29	-1.93	0.00556

Cyb5r3	37.4	1.37	46.52	1.90	1.24	0.01416
Cybb	1.2	0.10	2.09	0.15	1.72	0.00184
Cyc1	489.1	14.14	397.42	8.43	-1.23	0.00538
Cygb	22.4	1.13	31.94	1.91	1.42	0.00327
Cyp26b1	1.7	0.53	0.33	0.08	-5.22	0.00652
Cyp2b10	0.7	0.11	1.62	0.36	2.32	0.06084
Cyp4v3	0.6	0.03	0.89	0.05	1.38	0.02807
Cysltr1	0.6	0.07	1.55	0.13	2.74	0.00047
Cyth1	16.2	0.32	13.59	0.68	-1.20	0.03198
Cyth3	12.2	0.29	14.68	0.61	1.20	0.02470
Cyth4	2.6	0.10	4.00	0.30	1.50	0.00473
D030056L22Rik	3.1	0.12	2.41	0.17	-1.27	0.07569
D10Jhu81e	169.5	3.45	137.07	1.16	-1.24	0.00227
D2hgdh	9.3	0.34	6.95	0.54	-1.35	0.00977
D430019H16Rik	0.0	0.00	0.09	0.02	3.32	0.00885
D430040D24Rik	1.7	0.22	0.87	0.08	-1.82	0.02927
D7Ertd443e	3.4	0.29	5.18	0.34	1.57	0.00948
D8Ertd82e	3.7	0.19	2.33	0.11	-1.56	0.00074
Dab2	18.3	1.54	29.51	0.78	1.62	0.00066
Dach1	2.3	0.19	1.74	0.06	-1.29	0.05017
Dact1	0.8	0.08	1.64	0.16	2.02	0.00242
Dagla	1.6	0.14	2.09	0.14	1.31	0.07378
Dap	17.0	0.47	24.07	1.15	1.41	0.00178
Dap3	66.9	2.40	54.02	1.25	-1.24	0.00828
Dazap1	32.8	0.71	27.37	1.03	-1.20	0.01731
Dbh	1.1	0.07	0.62	0.12	-2.01	0.01114
Dbi	141.9	11.20	106.09	3.56	-1.32	0.01646
Dbn1	4.9	0.62	9.28	0.81	1.88	0.00269
Dcaf11	86.1	3.26	71.48	2.29	-1.21	0.02424
Dcbld1	1.3	0.15	1.66	0.10	1.30	0.07743
Dclk1	0.6	0.04	1.05	0.14	1.70	0.02448
Dclre1c	0.9	0.02	1.25	0.09	1.29	0.06995
Dcn	185.0	4.31	248.84	9.79	1.34	0.00114
Dct	1.3	0.16	2.77	0.43	2.24	0.00632
Dcun1d3	2.2	0.13	2.82	0.10	1.28	0.03213
Dcxr	9.4	0.40	7.35	0.76	-1.35	0.04215
Ddah2	13.0	0.37	17.20	1.28	1.30	0.02268
Ddo	8.5	0.30	6.79	0.29	-1.24	0.01731
Ddr2	7.3	0.56	10.44	0.22	1.45	0.00273
Ddt	50.4	2.33	41.33	1.81	-1.23	0.03703
Ddx59	3.1	0.17	2.50	0.12	-1.27	0.05603
Ddx60	0.9	0.06	1.27	0.10	1.34	0.09266
Decr1	66.7	2.99	43.76	1.36	-1.52	0.00019
Dennd4a	0.6	0.03	0.86	0.06	1.33	0.03262
Dennd5b	2.3	0.18	2.65	0.03	1.20	0.08677
Dennd6b	3.1	0.10	2.58	0.09	-1.22	0.03041

Dffa	14.3	0.22	17.98	0.62	1.26	0.00506
Dffb	3.7	0.28	2.67	0.19	-1.40	0.04156
Dgat2	108.1	4.89	83.77	2.81	-1.29	0.00654
Dgka	5.2	0.19	3.99	0.15	-1.32	0.00557
Dgkb	0.1	0.01	0.17	0.02	1.65	0.06943
Dgke	2.6	0.10	2.06	0.10	-1.29	0.01151
Dhcr24	0.7	0.07	1.22	0.08	1.67	0.00638
Dhrs11	51.9	3.33	36.75	2.33	-1.41	0.00638
Dhrs13	0.9	0.09	0.63	0.06	-1.47	0.08019
Dhrs4	32.0	1.45	22.83	0.63	-1.40	0.00088
Dhrs7c	63.1	3.39	48.52	2.43	-1.30	0.01312
Diap2	2.1	0.19	2.53	0.14	1.25	0.09182
Dio2	0.2	0.01	0.49	0.07	2.58	0.00091
Dis3l	9.7	0.37	8.07	0.24	-1.21	0.02437
Dixdc1	2.0	0.17	1.18	0.10	-1.64	0.00539
Dlg2	0.2	0.02	0.43	0.02	2.19	0.00334
Dlg4	2.8	0.14	4.25	0.39	1.50	0.02208
Dll1	3.2	0.33	1.88	0.23	-1.84	0.00927
Dll4	8.5	0.50	4.09	0.16	-2.07	0.00003
Dmap1	6.5	0.23	5.23	0.21	-1.23	0.03571
Dnaaf3	3.6	0.44	2.25	0.25	-1.61	0.02397
Dnah2	0.1	0.01	0.02	0.00	-2.88	0.00216
Dnajb9	16.8	1.04	10.88	0.62	-1.55	0.00115
Dnajc10	5.8	0.10	7.48	0.42	1.27	0.01498
Dnajc15	102.1	4.90	82.26	3.28	-1.24	0.02276
Dnajc19	76.1	4.28	60.96	2.56	-1.25	0.03135
Dnajc28	15.1	0.12	12.64	0.71	-1.21	0.03520
Dnajc9	4.4	0.19	3.45	0.15	-1.29	0.02226
Dnm1	6.7	0.53	9.17	1.00	1.36	0.08336
Dnm3	2.2	0.29	3.06	0.23	1.45	0.03176
Dnm3os	0.4	0.02	0.70	0.04	1.62	0.00353
Doc2g	127.4	4.56	105.73	3.52	-1.21	0.02306
Dock1	4.5	0.19	5.74	0.25	1.26	0.01603
Dock10	0.7	0.06	1.33	0.05	1.85	0.00075
Dock11	0.8	0.06	1.07	0.05	1.27	0.09718
Dock2	1.8	0.14	3.22	0.15	1.80	0.00097
Dohh	19.9	0.23	16.26	0.28	-1.23	0.00469
Dok1	0.9	0.09	1.37	0.10	1.41	0.06649
Dpcd	6.8	0.12	5.63	0.11	-1.22	0.04897
Dpp3	4.4	0.17	5.49	0.26	1.25	0.02828
Dpp7	3.0	0.10	4.05	0.27	1.33	0.04967
Dpy19l3	2.1	0.23	2.98	0.25	1.43	0.08725
Dpyd	2.0	0.06	1.58	0.11	-1.32	0.05214
Dpysl3	10.4	0.55	16.71	0.88	1.61	0.00039
Dram1	2.4	0.10	4.51	0.26	1.82	0.00049
Drd2	0.5	0.10	0.25	0.06	-2.19	0.08667

Dse	0.9	0.05	1.68	0.12	1.80	0.00066
Dsg2	11.2	0.26	8.37	0.62	-1.36	0.00761
Dtx3l	2.3	0.13	2.96	0.14	1.30	0.02072
Dus4l	2.7	0.11	2.10	0.06	-1.28	0.03249
Dusp1	94.4	10.32	64.74	5.78	-1.45	0.03940
Dusp22	7.4	0.30	8.90	0.41	1.20	0.09108
Dusp23	7.0	0.37	5.74	0.43	-1.27	0.06604
Dusp6	5.5	0.31	7.54	0.12	1.37	0.00216
Dut	6.2	0.76	4.24	0.20	-1.38	0.07634
Dxo	10.1	0.54	7.79	0.42	-1.30	0.03821
Dync1li2	8.5	0.31	10.34	0.60	1.21	0.05791
Dzip1	0.5	0.03	0.63	0.04	1.36	0.07788
Dzip1l	0.9	0.05	1.18	0.07	1.27	0.08816
E130102H24Rik	3.7	0.35	2.50	0.15	-1.38	0.08539
E2f3	2.0	0.10	2.67	0.16	1.29	0.04172
Ears2	4.1	0.11	3.42	0.13	-1.21	0.05118
Ebf1	10.0	0.46	14.38	0.32	1.45	0.00054
Ebf2	1.4	0.07	1.70	0.07	1.26	0.04008
Ech1	1143.4	69.26	638.33	33.59	-1.79	0.00010
Echdc2	41.2	2.68	30.59	1.89	-1.37	0.01306
Echdc3	11.0	0.38	8.64	0.56	-1.29	0.01956
Echs1	249.4	9.47	195.83	2.47	-1.27	0.00295
Eci1	147.6	6.09	92.46	4.40	-1.60	0.00022
Eci2	96.3	4.35	71.06	1.70	-1.35	0.00151
Ecm1	20.4	1.67	35.14	2.02	1.71	0.00079
Ecscr	12.0	0.14	15.74	1.32	1.27	0.04904
Edf1	151.4	3.18	125.54	5.16	-1.21	0.01757
Edn1	1.2	0.10	4.03	0.51	3.39	0.00013
Edn3	1.2	0.22	3.40	0.85	2.86	0.03498
Ednrb	3.7	0.13	2.79	0.16	-1.32	0.01059
Eef1a1	460.1	20.34	564.82	19.21	1.23	0.01796
Eepd1	8.9	0.66	6.56	0.33	-1.36	0.01677
Efemp1	4.9	0.61	3.03	0.49	-1.85	0.03158
Efemp2	7.6	0.35	11.78	0.58	1.54	0.00126
Efhd2	7.8	0.56	11.32	0.88	1.43	0.01390
Efna1	14.4	1.48	10.21	0.56	-1.41	0.03429
Efnb3	17.6	0.83	9.59	1.40	-1.90	0.00149
Efr3b	2.8	0.24	3.47	0.20	1.27	0.07338
Egflam	5.6	0.24	4.24	0.20	-1.32	0.00901
Egln1	83.3	2.10	64.23	2.54	-1.30	0.00269
Egln3	17.0	0.71	7.01	1.15	-2.53	0.00039
Egr1	81.6	14.15	45.76	6.41	-1.73	0.04329
Ehd1	57.8	2.06	47.56	2.46	-1.22	0.03287
Ehd3	0.6	0.04	0.81	0.04	1.28	0.08096
Ehhadh	3.4	0.41	2.32	0.18	-1.46	0.04072
Eif1	247.4	7.08	195.98	10.13	-1.27	0.01058

Eif3f	47.7	0.87	38.42	2.34	-1.25	0.02089
Eif3m	30.4	1.28	24.79	0.72	-1.22	0.02097
Eif4ebp2	19.5	0.75	16.13	0.86	-1.22	0.04207
Elf4	1.7	0.09	2.53	0.12	1.51	0.00161
Elmo1	2.9	0.14	3.80	0.17	1.34	0.01166
Elmod3	7.9	0.33	6.31	0.40	-1.28	0.03315
Eln	11.4	0.99	21.16	3.67	1.79	0.00829
Emc9	29.5	0.81	22.42	1.09	-1.31	0.00594
Emcn	29.5	1.88	45.40	2.10	1.55	0.00090
Emilin1	9.3	0.35	14.08	0.80	1.51	0.00102
Eml4	1.2	0.07	1.49	0.06	1.26	0.05583
Emp1	12.3	0.42	25.80	1.84	2.08	0.00002
Emp3	15.3	0.84	21.91	1.10	1.43	0.00363
Enah	25.5	1.14	37.18	3.70	1.44	0.01153
Endod1	1.4	0.11	2.25	0.06	1.63	0.00107
Endog	18.1	0.82	9.51	0.89	-1.97	0.00018
Eng	66.8	1.33	124.80	8.51	1.85	0.00005
Eno3	703.2	17.98	545.08	23.35	-1.29	0.00436
Enpp1	0.9	0.03	1.24	0.08	1.32	0.03297
Enpp6	0.6	0.05	1.02	0.10	1.55	0.04795
Entpd1	6.8	0.27	12.17	0.74	1.77	0.00017
Entpd5	23.8	0.80	19.20	1.78	-1.26	0.05952
Epb4.1	33.9	1.17	28.00	1.34	-1.21	0.03196
Epb4.1l2	7.0	0.11	9.23	0.27	1.32	0.00091
Epb4.1l4b	1.0	0.08	0.57	0.09	-1.85	0.02065
Epha2	3.0	0.17	4.45	0.32	1.46	0.00689
Ephb1	0.8	0.10	0.47	0.04	-1.85	0.01061
Ephb3	3.1	0.26	4.08	0.31	1.31	0.06416
Ephx1	22.8	1.76	32.76	1.42	1.44	0.00477
Ephx2	85.1	4.80	56.12	1.42	-1.51	0.00035
Ephx3	1.1	0.04	4.15	0.39	3.92	0.00002
Epsti1	0.4	0.06	1.03	0.13	2.34	0.00646
Erdr1	44.2	3.15	32.06	4.31	-1.41	0.07393
Esm1	0.3	0.06	1.14	0.20	3.70	0.00249
Espn	1.1	0.03	0.80	0.07	-1.40	0.04590
Esyt1	6.4	0.46	8.62	0.46	1.34	0.01862
Etfa	376.2	18.42	280.22	6.44	-1.34	0.00185
Etfb	481.7	19.33	321.21	9.60	-1.50	0.00024
Etfdh	284.4	15.24	225.84	8.19	-1.26	0.01558
Etl4	22.8	1.14	18.31	1.31	-1.25	0.05784
Ets2	28.3	1.05	22.84	0.42	-1.24	0.00746
Etv6	3.6	0.19	4.62	0.07	1.30	0.00826
Evc	1.4	0.15	2.05	0.12	1.41	0.03675
Evi2a	1.8	0.16	2.91	0.26	1.61	0.02688
Exd1	0.1	0.02	0.22	0.03	1.78	0.05794
Exoc3l2	0.2	0.04	0.94	0.05	4.39	0.00008

Exosc5	7.3	0.35	5.87	0.29	-1.25	0.05744
Ext1	7.3	0.07	6.08	0.29	-1.21	0.02916
F13a1	6.7	0.42	10.17	0.15	1.53	0.00048
F2r	6.6	0.16	8.32	0.44	1.26	0.01576
F2rl1	0.3	0.03	0.43	0.01	1.64	0.03928
Fads1	4.1	0.20	5.39	0.30	1.32	0.01553
Fads2	2.4	0.18	3.65	0.24	1.43	0.03429
Fah	14.4	0.93	7.05	0.31	-2.03	0.00003
Fahd2a	22.8	0.82	18.13	0.59	-1.26	0.01403
Fam102a	4.1	0.27	2.46	0.11	-1.67	0.00066
Fam111a	1.1	0.04	1.38	0.03	1.24	0.04729
Fam114a1	5.2	0.32	7.90	0.22	1.52	0.00091
Fam120aos	1.9	0.20	1.17	0.08	-1.64	0.01596
Fam124a	5.2	0.43	8.97	0.42	1.73	0.00073
Fam129b	8.4	0.27	11.99	0.74	1.42	0.00271
Fam131a	10.5	0.25	8.01	0.29	-1.31	0.00416
Fam13c	1.5	0.05	2.17	0.11	1.44	0.00552
Fam174b	62.0	2.94	51.11	3.73	-1.22	0.08500
Fam177a	4.1	0.15	5.15	0.15	1.26	0.01200
Fam189b	2.5	0.14	3.26	0.26	1.32	0.07534
Fam195a	116.5	2.10	87.57	4.32	-1.34	0.00173
Fam198a	0.1	0.03	0.33	0.04	2.64	0.00638
Fam198b	4.6	0.26	9.07	0.75	1.95	0.00021
Fam19a5	1.4	0.09	1.09	0.06	-1.33	0.04897
Fam21	3.7	0.13	4.81	0.21	1.31	0.00554
Fam222a	0.9	0.11	0.60	0.07	-1.53	0.07787
Fam26e	0.3	0.06	0.64	0.08	2.38	0.00755
Fam43a	8.6	0.52	6.75	0.20	-1.26	0.02108
Fam57b	3.1	0.18	4.18	0.17	1.34	0.01295
Fam65c	0.2	0.01	0.48	0.09	1.89	0.03150
Fam81a	2.5	0.19	3.32	0.17	1.39	0.01596
Fam84b	1.0	0.12	0.63	0.06	-1.50	0.05740
Fancb	0.4	0.04	0.20	0.03	-1.88	0.04178
Fancl	2.9	0.11	1.98	0.07	-1.46	0.01009
Farp1	3.1	0.22	3.73	0.05	1.21	0.09975
Fasn	7.6	0.30	5.85	0.38	-1.29	0.02188
Fat1	4.1	0.17	6.86	0.34	1.68	0.00010
Fat4	1.2	0.14	1.64	0.14	1.44	0.04626
Fbln2	36.5	2.02	66.55	3.71	1.82	0.00010
Fbln5	3.6	0.27	5.63	0.25	1.56	0.00096
Fbn1	7.3	0.67	14.80	1.54	2.00	0.00074
Fbp2	13.0	1.01	6.41	0.63	-2.06	0.00038
Fbxl22	13.4	0.47	9.89	0.63	-1.36	0.00582
Fbxo17	0.6	0.04	0.37	0.06	-1.62	0.06278
Fbxo21	15.5	0.65	12.14	0.45	-1.28	0.00709
Fbxw17	4.1	0.20	5.46	0.29	1.31	0.07613

Fcer1g	12.8	0.56	19.52	0.94	1.48	0.00169
Fcgr1	1.8	0.13	3.66	0.40	1.84	0.00907
Fcgr2b	4.1	0.18	7.29	0.49	1.72	0.00107
Fcgr3	6.7	0.34	10.13	0.96	1.45	0.01171
Fcna	0.8	0.07	1.42	0.16	1.61	0.06458
Fcrls	1.5	0.11	2.88	0.19	1.86	0.00082
Fdft1	10.5	0.59	8.63	0.16	-1.22	0.03429
Fdx1	30.1	2.01	18.93	0.91	-1.58	0.00115
Fermt3	1.5	0.06	2.23	0.14	1.48	0.00729
Fetub	0.4	0.03	0.89	0.23	2.41	0.03943
Fgd3	0.4	0.03	0.56	0.02	1.33	0.07304
Fgf13	43.4	1.53	32.59	1.85	-1.34	0.00530
Fgf16	5.8	0.58	3.49	0.53	-1.65	0.03012
Fgf2	3.4	0.14	2.70	0.22	-1.26	0.06570
Fgl2	6.2	0.31	10.16	0.61	1.64	0.00051
Fh1	249.5	8.76	191.59	4.63	-1.30	0.00193
Fhl1	44.0	2.18	56.02	5.14	1.26	0.07807
Fhod3	40.7	0.82	31.34	1.35	-1.30	0.00257
Fibin	1.9	0.20	4.07	0.75	2.07	0.01671
Figf	1.1	0.06	3.81	0.17	3.55	0.00000
Fitm1	79.1	2.02	58.01	2.94	-1.37	0.00179
Fkrp	13.3	0.64	10.93	0.37	-1.21	0.03503
Flcn	18.9	1.20	15.38	0.75	-1.23	0.05080
Flna	51.5	2.15	72.62	3.49	1.41	0.00181
Flnb	5.3	0.25	7.78	0.49	1.45	0.00242
Flnc	56.1	3.74	67.23	2.89	1.20	0.09132
Flrt2	0.6	0.04	0.93	0.07	1.50	0.01153
Flt3l	10.5	1.09	16.31	0.83	1.56	0.00674
Fmo1	27.1	0.88	22.21	0.91	-1.22	0.02153
Fmo2	6.7	0.48	10.53	0.50	1.59	0.00123
Fmod	1.0	0.14	1.63	0.16	1.64	0.02929
Fn1	8.0	0.22	20.48	1.62	2.51	0.00000
Fn3k	11.2	0.29	9.05	0.31	-1.26	0.01540
Fndc1	4.7	0.33	8.38	0.59	1.75	0.00053
Fnip2	5.3	0.25	6.67	0.34	1.26	0.02771
Folr2	7.6	0.68	10.95	0.45	1.43	0.01049
Foxp1	16.0	0.81	19.62	1.29	1.23	0.07963
Frmd6	5.9	0.41	7.45	0.30	1.27	0.04209
Frs3	4.0	0.19	2.86	0.17	-1.37	0.02518
Frzb	1.2	0.04	3.89	0.55	2.92	0.00048
Fstl1	48.8	1.29	96.06	5.71	1.95	0.00002
Fstl3	1.6	0.10	2.50	0.30	1.60	0.03770
Fstl4	0.9	0.08	1.30	0.14	1.43	0.06661
Fth1	1709.7	78.77	1241.46	59.75	-1.38	0.00247
Fuom	30.6	2.05	23.30	0.80	-1.31	0.02095
Fut8	4.2	0.13	5.06	0.15	1.21	0.02706

Fxn	13.9	0.68	11.50	0.23	-1.22	0.03076
Fxyd5	28.8	1.53	50.77	3.31	1.73	0.00038
Fxyd6	9.9	0.28	13.42	0.99	1.34	0.01687
Fyb	45.3	3.48	83.79	3.42	1.79	0.00114
Fzd1	1.3	0.08	1.88	0.11	1.50	0.00554
Fzd2	1.3	0.10	1.97	0.27	1.52	0.07393
Fzd7	1.1	0.02	1.32	0.07	1.23	0.06792
Fzd8	1.9	0.10	1.27	0.10	-1.57	0.00693
G6pdx	3.2	0.17	4.00	0.20	1.22	0.09822
Gab2	4.1	0.13	5.64	0.15	1.36	0.00083
Gabra3	1.1	0.19	2.90	0.15	2.76	0.00062
Gadd45gip1	10.5	0.54	7.28	0.32	-1.43	0.00224
Gal3st3	8.8	0.66	6.80	0.50	-1.28	0.07202
Galnt16	1.2	0.05	2.09	0.11	1.70	0.00050
Galnt18	4.8	0.32	3.37	0.25	-1.46	0.01295
Galt	30.4	1.55	25.21	0.33	-1.20	0.03356
Gas1	6.4	0.33	4.11	0.44	-1.58	0.00282
Gas2l3	0.3	0.07	0.72	0.09	2.90	0.00177
Gas7	2.0	0.07	3.27	0.14	1.60	0.00021
Gata6	14.6	0.76	11.59	0.65	-1.26	0.02962
Gba2	7.9	0.29	6.42	0.32	-1.26	0.02013
Gbas	414.1	31.70	297.18	9.36	-1.38	0.00614
Gbp2	4.8	0.25	6.42	0.26	1.32	0.01167
Gbp3	3.0	0.23	4.34	0.12	1.47	0.00556
Gbp4	3.4	0.36	6.01	0.54	1.79	0.00282
Gbp5	2.3	0.21	3.04	0.23	1.35	0.08117
Gbp6	3.8	0.49	6.66	0.52	1.79	0.01002
Gbp7	1.9	0.13	2.93	0.10	1.54	0.00115
Gcat	4.8	0.42	2.98	0.15	-1.60	0.00344
Gcdh	31.1	0.97	23.65	0.55	-1.32	0.00131
Gck	10.9	1.06	17.46	2.64	1.59	0.03930
Gcnt1	0.4	0.03	0.70	0.09	1.75	0.02858
Gcnt2	1.3	0.09	2.29	0.13	1.78	0.00069
Gdf15	0.7	0.11	2.35	0.49	3.09	0.01087
Gdf6	0.2	0.02	0.55	0.08	2.97	0.00138
Get4	20.8	0.66	16.52	0.33	-1.26	0.00479
Gfra4	6.0	0.50	2.87	0.29	-2.09	0.00107
Ggnbp1	32.7	0.99	23.65	1.30	-1.40	0.00126
Ggt7	0.9	0.07	1.52	0.11	1.74	0.01118
Gja4	4.7	0.41	6.02	0.29	1.25	0.09192
Gli1	0.4	0.06	0.78	0.04	2.13	0.00286
Gli2	0.8	0.11	1.41	0.10	1.69	0.01797
Gli3	0.3	0.03	0.62	0.02	1.82	0.00147
Glis2	7.4	0.32	9.49	0.56	1.27	0.03294
Gltp	9.3	0.57	12.21	0.61	1.31	0.02562
Gltscr2	58.6	1.89	48.15	2.59	-1.22	0.03389

Gm10138	2.1	0.17	1.51	0.09	-1.37	0.03980
Gm10435	31.2	4.70	18.97	1.50	-1.60	0.02804
Gm10603	0.8	0.11	0.51	0.02	-1.48	0.06834
Gm10699	0.3	0.05	0.58	0.03	2.00	0.01709
Gm10925	32735.7	3603.25	17363.76	2242.95	-1.94	0.00649
Gm11407	473.7	90.70	234.48	28.40	-1.93	0.04907
Gm11627	1.2	0.26	2.23	0.35	1.86	0.08773
Gm11716	24.2	2.10	17.42	1.62	-1.34	0.08200
Gm11730	3.2	0.46	1.16	0.13	-2.59	0.00215
Gm12002	8.2	0.61	4.04	0.46	-2.04	0.00053
Gm12158	4.6	0.29	7.09	0.99	1.46	0.06504
Gm12473	9.1	0.67	6.87	0.45	-1.35	0.05844
Gm12519	1.9	0.32	1.02	0.11	-1.77	0.08000
Gm12655	3.3	0.31	2.31	0.22	-1.53	0.06408
Gm12840	44.4	7.57	60.28	2.97	1.47	0.04052
Gm13111	1.6	0.12	1.26	0.16	-1.35	0.09802
Gm13154	1.2	0.21	0.43	0.15	-3.84	0.05239
Gm13340	0.0	0.00	0.00	0.00	-1.81	0.00540
Gm13375	14.9	0.89	12.48	0.22	-1.21	0.06851
Gm13479	0.4	0.06	0.27	0.02	-1.58	0.04972
Gm13943	11.2	0.79	26.95	4.96	2.28	0.02875
Gm14005	4.0	0.48	6.36	0.31	1.59	0.03575
Gm14261	3.4	0.64	2.01	0.26	-1.78	0.06202
Gm14290	5.7	0.41	4.43	0.23	-1.26	0.06276
Gm14326	4.2	0.42	5.92	0.33	1.44	0.08773
Gm14453	30.1	3.55	17.81	1.63	-1.63	0.04526
Gm15545	2.0	0.31	3.16	0.32	1.57	0.09107
Gm15587	3.6	0.29	2.21	0.39	-1.82	0.01403
Gm15743	2.0	0.42	3.54	0.48	1.86	0.04904
Gm15856	18.7	1.53	11.74	1.17	-1.53	0.07361
Gm16091	0.6	0.08	1.22	0.10	1.88	0.01460
Gm16316	0.7	0.04	0.55	0.04	-1.42	0.06483
Gm16576	1.1	0.04	0.81	0.04	-1.33	0.01416
Gm16685	0.2	0.05	0.29	0.04	1.93	0.07157
Gm16897	1.9	0.36	3.07	0.18	1.66	0.09249
Gm16907	2.1	0.35	3.64	0.29	1.81	0.01026
Gm21747	13.2	0.95	9.83	1.42	-1.46	0.07846
Gm22710	533.4	65.75	242.05	32.56	-2.15	0.00766
Gm26603	0.2	0.03	0.30	0.02	1.60	0.06563
Gm26782	2.2	0.29	3.04	0.36	1.46	0.08644
Gm26862	0.6	0.08	0.34	0.03	-1.65	0.04435
Gm28437	52878.8	7393.21	31303.14	3485.45	-1.62	0.04958
Gm28661	36625.8	5893.51	18996.19	3812.64	-1.91	0.04849
Gm29216	0.0	0.00	0.00	0.00	-1.87	0.02611
Gm3235	2.3	0.18	1.55	0.09	-1.51	0.05705
Gm34302	192.9	10.11	121.25	7.62	-1.64	0.00094

Gm37018	0.3	0.03	0.42	0.04	1.63	0.04352
Gm37058	0.4	0.09	0.67	0.09	1.92	0.07176
Gm37606	0.6	0.04	0.96	0.13	1.77	0.02453
Gm37691	2.2	0.14	1.61	0.10	-1.33	0.03909
Gm37697	3.2	0.30	2.33	0.13	-1.34	0.07534
Gm37761	0.4	0.06	0.64	0.03	1.60	0.06661
Gm38102	11.9	1.08	7.97	0.83	-1.47	0.09372
Gm38312	5.3	0.20	3.58	0.22	-1.50	0.00326
Gm4076	1078.8	76.48	699.57	103.86	-1.65	0.06034
Gm4841	0.3	0.07	0.76	0.26	2.38	0.09921
Gm4951	0.5	0.07	1.04	0.05	2.19	0.00707
Gm5617	13.0	0.27	10.63	0.63	-1.23	0.06265
Gm572	1.2	0.18	0.85	0.08	-1.51	0.08248
Gm6086	0.5	0.03	0.33	0.03	-1.57	0.00674
Gm694	0.6	0.10	2.05	0.22	3.12	0.00186
Gm7694	2.9	0.09	2.29	0.14	-1.26	0.03293
Gm8995	1.1	0.09	1.33	0.03	1.26	0.05909
Gnao1	11.5	0.68	18.94	1.99	1.63	0.00316
Gnas	444.1	45.86	231.42	28.31	-1.96	0.00425
Gnb3	4.2	0.19	5.95	0.62	1.42	0.03943
Gnmt	3.1	0.36	1.47	0.13	-2.11	0.00489
Golga4	42.6	2.80	55.40	5.37	1.29	0.08853
Golim4	13.1	0.56	16.37	0.52	1.26	0.01200
Got1	423.7	21.51	350.81	14.54	-1.21	0.04161
Got2	246.6	7.33	199.90	5.70	-1.24	0.00668
Gp49a	2.6	0.14	5.05	0.19	1.95	0.00022
Gpc6	1.7	0.09	2.26	0.10	1.37	0.00691
Gpihbp1	85.7	5.16	65.20	5.33	-1.33	0.02809
Gpm6b	2.0	0.16	4.52	0.37	2.23	0.00033
Gpnmb	0.4	0.06	0.66	0.10	1.66	0.08816
Gpr153	5.2	0.33	6.77	0.31	1.29	0.02614
Gpr161	0.3	0.02	0.63	0.05	1.87	0.00107
Gpr22	7.6	0.61	5.19	0.58	-1.48	0.02708
Gpr27	3.4	0.52	2.18	0.36	-1.62	0.06379
Gpr34	0.4	0.09	0.76	0.10	2.25	0.08039
Gpr39	0.1	0.02	0.71	0.06	10.75	0.00028
Gpr65	0.4	0.02	0.67	0.04	1.52	0.02931
Gpsm3	2.0	0.09	3.59	0.32	1.79	0.00343
Gpt	10.8	0.67	7.17	0.66	-1.53	0.00661
Gpt2	7.1	0.51	5.22	0.30	-1.35	0.02136
Gpx1	192.1	10.72	259.17	22.51	1.33	0.03052
Gramd1b	19.6	0.56	15.22	0.68	-1.29	0.00637
Gramd4	11.6	0.53	8.70	0.41	-1.35	0.00478
Grap	4.0	0.18	2.81	0.15	-1.41	0.00714
Grasp	5.4	0.55	3.99	0.14	-1.30	0.06228
Grb14	67.1	1.02	51.16	1.72	-1.32	0.00112

Grcc10	178.5	4.48	120.58	4.29	-1.51	0.00049
Gria3	0.7	0.05	1.11	0.05	1.55	0.02428
Grn	41.8	2.38	54.40	3.19	1.30	0.02577
Gstk1	57.3	2.71	39.41	1.02	-1.45	0.00054
Gstm7	32.8	0.99	23.62	1.02	-1.39	0.00111
Gstp1	79.1	4.34	64.06	3.48	-1.23	0.06278
Gstp2	50.7	4.34	29.98	5.98	-1.72	0.05784
Gstz1	19.6	0.87	15.91	0.46	-1.23	0.01853
Gtf3a	5.3	0.38	3.98	0.20	-1.33	0.05302
Gtpbp4	4.9	0.27	6.04	0.30	1.25	0.04155
Gtpbp6	11.6	0.44	8.21	0.74	-1.41	0.02490
Gtpbp8	3.9	0.23	3.05	0.15	-1.30	0.03795
Gucy1a3	4.0	0.19	5.58	0.38	1.37	0.00994
Gucy1b3	4.9	0.30	6.30	0.29	1.27	0.02969
Gulp1	3.7	0.46	5.60	0.35	1.45	0.04650
Gxylt1	1.3	0.03	1.67	0.05	1.30	0.00529
Gypc	16.0	0.38	13.18	0.45	-1.22	0.01316
H2afj	6.5	0.32	3.92	0.31	-1.64	0.00247
H2-D1	43.4	1.37	67.32	4.57	1.54	0.00092
H2-K1	51.7	2.89	89.47	5.65	1.73	0.00037
H2-K2	2.4	0.12	1.49	0.14	-1.70	0.01853
H2-M3	1.4	0.15	2.06	0.18	1.49	0.05736
H2-Q10	0.6	0.08	0.87	0.04	1.62	0.05423
H2-Q4	13.9	0.63	19.88	1.06	1.44	0.00251
H2-Q5	6.4	0.60	9.53	0.59	1.48	0.03099
H2-Q6	5.6	0.78	10.58	0.91	1.93	0.00509
H2-Q7	6.9	0.86	12.51	0.83	1.82	0.00211
H2-T22	5.0	0.31	7.54	0.40	1.51	0.00364
H2-T23	10.0	0.39	12.57	0.84	1.26	0.04239
Hadh	192.6	8.99	144.18	2.78	-1.33	0.00181
Hadha	621.2	24.96	461.13	10.95	-1.35	0.00103
Hadhb	781.1	23.24	569.93	6.52	-1.37	0.00031
Haus8	14.4	1.13	21.08	1.78	1.49	0.00990
Hck	0.6	0.03	0.91	0.05	1.46	0.03429
Hcls1	4.0	0.14	5.11	0.36	1.25	0.05810
Hcn2	5.8	0.55	3.90	0.42	-1.46	0.04492
Hdac5	46.2	1.44	37.91	1.29	-1.22	0.01416
Hdac9	3.8	0.24	3.04	0.25	-1.27	0.08575
Heg1	19.4	1.52	27.84	1.63	1.44	0.00654
Heph	0.2	0.02	0.39	0.04	1.79	0.01256
Herpud1	126.4	10.18	74.70	3.75	-1.68	0.00073
Hes1	27.1	2.20	19.14	2.10	-1.37	0.06084
Hes6	7.4	0.21	6.14	0.37	-1.22	0.08852
Hey1	5.4	0.32	7.34	0.57	1.38	0.02090
Hfe2	78.8	3.65	63.03	3.20	-1.25	0.02230
Hhatl	47.3	2.47	35.27	2.04	-1.34	0.00804

Hhex	1.3	0.05	0.83	0.13	-1.51	0.07190
Hils1	2.8	0.30	1.79	0.25	-1.53	0.08161
Hint1	136.6	2.77	113.97	3.55	-1.20	0.01351
Hint2	72.4	2.93	51.27	3.16	-1.42	0.00255
Hist2h2be	2.0	0.08	1.65	0.02	-1.21	0.06910
Hk3	0.4	0.07	0.79	0.03	1.83	0.02077
Hlx	5.1	0.63	3.19	0.46	-1.54	0.07248
Hmbs	13.2	0.35	10.86	0.28	-1.21	0.01540
Hmg20b	37.3	1.56	30.69	1.02	-1.22	0.02424
Hmgcs2	4.5	1.07	1.42	0.04	-2.79	0.00469
Hmgn2	37.1	3.45	27.51	0.80	-1.35	0.02176
Hmgn3	5.7	0.44	8.18	0.52	1.45	0.04685
Hmha1	1.6	0.10	2.42	0.18	1.48	0.00574
Hmox1	3.4	0.37	6.59	0.54	1.95	0.00097
Hnmt	3.4	0.14	2.01	0.05	-1.72	0.00039
Hnrnpa0	13.6	0.36	10.03	0.70	-1.37	0.00577
Hnrnpl	81.4	4.47	65.14	2.78	-1.25	0.02348
Hopx	85.3	2.48	70.89	3.80	-1.21	0.04590
Hpgds	0.5	0.08	1.16	0.08	2.18	0.00242
Hrc	361.1	10.44	282.76	10.84	-1.28	0.00439
Hrct1	2.6	0.12	1.89	0.13	-1.46	0.01515
Hs3st1	5.4	0.35	7.15	0.27	1.32	0.02506
Hsd11b1	8.8	0.65	6.55	0.33	-1.39	0.01401
Hsd17b10	99.4	3.27	75.38	2.19	-1.32	0.00150
Hsd17b11	8.4	1.00	5.80	0.17	-1.39	0.04284
Hsdl2	66.3	3.03	51.42	3.06	-1.29	0.01540
Hsf2	3.6	0.16	2.95	0.11	-1.21	0.06430
Hsp90b1	228.1	20.73	165.44	9.36	-1.37	0.02614
Hspa1l	0.6	0.06	1.00	0.10	1.64	0.03426
Hspa2	1.8	0.11	1.34	0.11	-1.33	0.05856
Hspa5	294.7	16.30	188.81	11.40	-1.56	0.00107
Hspb6	623.4	20.77	790.90	55.59	1.26	0.03581
Hspd1	63.0	2.70	52.51	3.81	-1.21	0.09595
Htra1	35.1	2.43	25.25	1.97	-1.39	0.01621
Hyal1	4.6	0.22	5.73	0.17	1.24	0.06503
Hyou1	18.4	1.60	13.08	0.70	-1.38	0.02963
Ica1	12.6	0.66	10.44	0.60	-1.24	0.08211
Icam1	3.8	0.08	5.93	0.21	1.54	0.00019
Icosl	2.4	0.15	3.01	0.15	1.24	0.07708
Ict1	65.3	2.12	52.62	1.88	-1.24	0.01053
Id3	27.7	0.79	37.37	1.57	1.35	0.00197
Idh3a	135.9	5.76	109.55	4.06	-1.24	0.01416
Idh3b	430.7	10.22	333.59	4.99	-1.29	0.00090
Idh3g	402.7	17.80	305.47	11.97	-1.32	0.00397
Ier2	37.9	4.91	20.36	2.03	-1.82	0.00582
Ifi203	8.9	0.41	10.62	0.21	1.21	0.02607

Ifi204	2.1	0.10	3.54	0.19	1.67	0.00231
Ifi27	22.6	1.11	31.13	0.44	1.39	0.00123
Ifi27l2a	21.3	1.62	34.96	2.19	1.66	0.00153
Ifi30	4.7	0.23	6.34	0.55	1.36	0.04696
Ifi47	1.0	0.06	2.19	0.34	2.00	0.00497
Ifit1	1.4	0.07	1.98	0.10	1.48	0.00646
Ifit2	1.9	0.16	3.83	0.16	1.97	0.00018
Ifit3	2.6	0.30	4.61	0.22	1.89	0.00104
Ifit3b	1.7	0.30	3.06	0.26	1.91	0.02721
Ifitm1	7.8	0.34	11.74	0.98	1.49	0.02453
Ifitm2	70.8	2.97	94.14	3.24	1.33	0.00303
Ifitm3	108.2	2.15	148.39	1.88	1.37	0.00022
Ifngr1	18.9	0.34	24.31	0.74	1.28	0.00230
Ift122	3.8	0.38	5.39	0.56	1.39	0.06661
Ift81	14.7	0.49	10.91	0.29	-1.35	0.00109
Igf1	3.8	0.29	7.91	0.54	2.11	0.00018
Igfbp3	7.6	0.25	2.37	0.18	-3.33	0.00000
Igfbp4	45.5	1.28	67.38	2.57	1.48	0.00023
Igfbp5	14.7	0.98	22.34	0.60	1.53	0.00088
Igfbp7	90.7	4.10	149.93	10.72	1.64	0.00049
Ighm	3.8	0.43	6.24	0.52	1.65	0.00927
Igkc	1.7	0.25	3.30	0.58	1.85	0.06430
Igsf1	4.5	0.17	8.57	0.78	1.89	0.00054
Igsf10	0.4	0.04	0.93	0.10	2.03	0.00511
Igsf8	8.6	0.50	10.56	0.46	1.22	0.07613
Igtp	4.5	0.35	7.75	0.51	1.74	0.00079
Ikzf1	1.1	0.11	1.62	0.16	1.47	0.06430
Il13ra1	5.9	0.06	8.13	0.26	1.37	0.00055
Il15	7.4	0.25	4.66	0.41	-1.62	0.00127
Il17ra	0.8	0.09	1.21	0.06	1.56	0.00649
Il18bp	5.0	0.55	6.40	0.22	1.32	0.04223
Il1rl2	0.5	0.03	0.66	0.03	1.35	0.05872
Il21r	0.3	0.03	0.80	0.04	2.33	0.00102
Il27ra	0.5	0.04	0.72	0.08	1.44	0.08657
Il34	5.7	0.39	7.62	0.26	1.38	0.03012
Il3ra	2.3	0.21	3.04	0.07	1.32	0.06406
Il6st	99.0	3.99	119.90	3.69	1.21	0.02518
Il7	0.2	0.04	0.39	0.03	2.02	0.02540
Ildr2	0.1	0.02	0.15	0.01	1.89	0.02328
Ilvbl	6.8	0.40	5.28	0.31	-1.31	0.02928
Immp1l	30.0	1.32	23.49	0.52	-1.28	0.00664
Imp3	19.7	0.98	15.96	1.07	-1.23	0.06851
Impa2	7.0	0.26	4.45	0.39	-1.59	0.00144
Inafm1	4.7	0.16	3.44	0.36	-1.36	0.05760
Inca1	3.8	0.14	3.07	0.21	-1.28	0.09803
Inmt	9.8	1.43	6.33	1.64	-1.76	0.09705

Ino80c	6.1	0.30	7.46	0.23	1.22	0.03066
Inpp5f	4.0	0.11	5.21	0.14	1.29	0.01118
Iqcb1	6.5	0.34	5.20	0.28	-1.25	0.09325
Iqgap1	5.5	0.23	7.65	0.19	1.39	0.00090
Irak3	1.8	0.11	2.43	0.17	1.34	0.05583
Irak4	1.4	0.04	1.96	0.08	1.37	0.01296
Irf2bp2	10.1	0.80	7.72	0.28	-1.30	0.04192
Irf5	8.2	0.31	13.61	1.43	1.59	0.00636
Irf7	6.0	0.60	8.86	0.52	1.51	0.00746
Irf8	3.0	0.64	3.86	1.94	1.28	0.04128
Irf9	8.1	0.51	10.41	0.28	1.30	0.01450
Irgm1	5.5	0.25	7.27	0.21	1.33	0.00649
Irgm2	4.6	0.18	6.56	0.37	1.43	0.00264
Irs3	0.1	0.04	0.30	0.04	2.69	0.02190
Irx2	6.8	0.44	3.88	0.40	-1.77	0.00208
Irx3	11.0	0.72	8.43	0.57	-1.30	0.03748
Islr	12.8	1.00	21.41	2.78	1.62	0.01806
Islr2	0.2	0.01	0.45	0.12	2.22	0.03673
Ism1	0.2	0.02	0.62	0.05	3.65	0.00014
Isoc1	8.9	0.41	6.85	0.20	-1.31	0.00540
Isoc2a	33.7	1.04	26.16	0.35	-1.29	0.00193
Isyna1	10.7	0.84	6.56	0.34	-1.64	0.00112
Itga9	9.3	0.48	13.91	0.88	1.48	0.00244
Itgal	0.1	0.01	0.19	0.02	1.55	0.06057
Itgam	1.9	0.08	2.91	0.15	1.51	0.00248
Itgb1bp2	76.6	2.46	62.64	1.90	-1.22	0.01192
Itgb2	2.1	0.14	3.24	0.26	1.46	0.01200
Itgb4	1.6	0.08	2.91	0.20	1.76	0.00125
Itgb7	0.1	0.02	0.23	0.04	2.22	0.03339
Itgb8	0.2	0.01	0.36	0.03	2.11	0.00321
Itgbl1	1.2	0.07	4.31	0.44	3.62	0.00003
Itih5	0.5	0.04	1.03	0.11	1.98	0.00195
Itm2a	2.4	0.11	3.17	0.22	1.27	0.07393
Itpr3	1.7	0.15	2.18	0.02	1.30	0.02778
Itpripl2	4.0	0.21	5.47	0.28	1.38	0.00556
Ivd	154.0	7.38	120.14	6.74	-1.28	0.01540
Jak3	8.2	0.27	6.71	0.52	-1.25	0.05732
Jdp2	2.7	0.35	3.82	0.13	1.47	0.02577
Jph1	4.0	0.22	3.30	0.14	-1.21	0.06161
Jtb	20.3	0.54	15.82	0.71	-1.29	0.00754
Junb	45.9	7.22	30.33	3.20	-1.47	0.08355
Jund	16.1	0.73	12.09	1.24	-1.36	0.03067
Kank3	43.9	1.32	29.42	1.89	-1.51	0.00057
Kank4	0.3	0.05	2.22	0.17	7.50	0.00001
Kbtbd12	6.1	0.23	7.60	0.70	1.27	0.08623
Kcna1	0.3	0.06	0.17	0.03	-2.14	0.04445

Kcna5	7.1	0.57	4.59	0.58	-1.63	0.01576
Kcnab2	1.6	0.37	3.08	0.26	2.00	0.01515
Kcnc1	0.1	0.02	0.36	0.06	2.42	0.00798
Kcnd2	2.8	0.13	2.06	0.14	-1.39	0.01013
Kcne1	9.1	1.01	13.45	2.02	1.42	0.08641
Kcne4	0.6	0.04	0.99	0.08	1.48	0.03339
Kcng2	70.3	1.87	53.13	1.51	-1.33	0.00107
Kcnip2	65.9	5.83	48.63	2.68	-1.35	0.02577
Kcnj12	7.1	0.28	5.58	0.28	-1.25	0.02688
Kcnj3	11.3	0.75	6.94	1.31	-1.70	0.02397
Kcnmb1	0.3	0.02	0.53	0.04	1.62	0.01002
Kcnn2	3.7	0.30	2.85	0.16	-1.33	0.03786
Kcnn3	0.2	0.02	0.51	0.08	1.89	0.03362
Kcnv2	1.9	0.13	1.04	0.10	-1.90	0.00069
Kctd11	0.6	0.07	1.11	0.04	1.86	0.00171
Kctd12b	1.8	0.15	2.67	0.22	1.51	0.00783
Kctd15	0.8	0.02	1.21	0.05	1.54	0.00693
Kctd17	13.1	0.90	18.04	0.87	1.38	0.01076
Kdm6b	5.7	0.32	4.68	0.24	-1.22	0.06265
Kdsr	1.2	0.02	1.49	0.06	1.24	0.03694
Khdrbs3	17.4	0.65	12.59	0.91	-1.38	0.00661
Kif1a	0.2	0.03	0.57	0.12	2.32	0.01604
Kif26b	0.3	0.04	0.52	0.06	1.79	0.01448
Kif3c	0.9	0.04	1.12	0.05	1.25	0.05959
Kif5b	32.0	2.55	46.68	7.01	1.42	0.07880
Kif5c	0.4	0.05	0.97	0.13	2.47	0.00472
Kirrel	1.8	0.10	2.86	0.17	1.60	0.00084
Kit	1.2	0.16	0.60	0.06	-2.05	0.00758
Kiz	40.3	0.96	30.11	1.25	-1.35	0.00147
Klf15	16.3	1.42	12.11	0.90	-1.34	0.04455
Klf2	40.6	3.66	26.39	2.83	-1.55	0.01411
Klhdc1	16.4	0.37	12.71	0.96	-1.30	0.01845
Klhl29	0.1	0.01	0.18	0.03	2.61	0.00416
Klhl34	1.6	0.19	3.12	0.51	1.93	0.02093
Klhl38	9.3	0.57	6.84	0.73	-1.40	0.03909
Klk8	2.3	0.18	1.20	0.12	-1.89	0.00469
Kmo	0.2	0.03	0.34	0.04	1.71	0.08943
Kptn	8.6	0.49	7.00	0.31	-1.22	0.06953
Kremen1	27.5	0.76	33.66	1.85	1.22	0.03078
Krt80	0.9	0.17	2.55	0.59	3.18	0.01704
L2hgdh	6.4	0.11	5.02	0.15	-1.27	0.00407
L3hypdh	2.6	0.18	1.95	0.18	-1.42	0.05794
Lad1	2.9	0.40	1.42	0.34	-2.19	0.01450
Lair1	0.6	0.08	0.88	0.07	1.55	0.04130
Lama3	0.4	0.04	1.10	0.15	2.93	0.00097
Lama5	32.2	2.77	23.13	1.87	-1.39	0.02859

Lamb3	5.1	0.44	3.58	0.33	-1.40	0.03323
Lamc3	0.1	0.02	0.42	0.05	3.80	0.00056
Laptm5	8.3	0.54	13.47	0.84	1.60	0.00114
Lasp1	16.4	0.76	22.63	1.20	1.37	0.00363
Lbp	2.5	0.28	4.75	0.11	2.00	0.00039
Lbr	4.7	0.24	3.91	0.24	-1.24	0.06170
Lcmt2	3.6	0.25	2.75	0.09	-1.28	0.04499
Lcn2	6.7	0.97	17.10	2.24	2.70	0.00077
Lcp1	6.0	0.52	9.95	0.39	1.64	0.00099
Ldb2	4.9	0.36	6.66	0.33	1.36	0.02638
Ldhb	1001.0	27.91	813.86	13.67	-1.23	0.00481
Ldhd	17.8	0.53	13.92	0.83	-1.30	0.01133
Ldlr	2.0	0.19	3.26	0.20	1.64	0.00286
Lfng	3.6	0.35	7.42	0.62	1.99	0.00071
Lgals3	2.1	0.45	3.92	0.53	1.84	0.04052
Lgals3bp	20.3	0.92	31.33	1.32	1.54	0.00037
Lgals4	28.3	6.01	12.55	0.79	-2.10	0.00869
Lgals9	12.7	0.47	15.99	1.33	1.23	0.09144
Lgi2	0.2	0.03	0.44	0.05	2.05	0.00603
Lgr6	8.3	0.51	6.21	0.50	-1.35	0.02706
Lhfpl2	0.7	0.06	1.60	0.14	2.13	0.00076
Lilrb4	2.0	0.20	3.55	0.29	1.76	0.00596
Lima1	5.7	0.23	7.87	0.32	1.39	0.00164
Limk1	3.0	0.11	3.96	0.10	1.29	0.01086
Lin7a	0.2	0.02	0.35	0.06	1.59	0.08088
Lingo3	4.0	0.23	2.71	0.28	-1.47	0.01576
Lipa	6.4	0.36	9.56	0.35	1.50	0.00084
Lipe	15.7	1.27	10.29	0.42	-1.51	0.00194
Llgl2	5.1	0.24	4.16	0.13	-1.22	0.03673
Lman1l	0.2	0.05	0.81	0.19	3.92	0.00931
Lmna	34.0	1.13	41.34	2.87	1.21	0.07773
Lmo7	102.7	1.36	80.66	2.99	-1.28	0.00267
Lmod1	0.6	0.03	0.98	0.07	1.62	0.00437
Lox	0.9	0.11	1.64	0.12	1.97	0.00363
Loxl1	12.4	0.85	20.43	1.33	1.63	0.00101
Loxl2	2.9	0.22	4.72	0.42	1.61	0.00869
Loxl3	1.2	0.09	1.85	0.10	1.55	0.00477
Loxl4	0.2	0.01	0.51	0.06	2.02	0.00326
Lpar1	4.2	0.22	8.40	0.36	1.97	0.00005
Lpar4	0.2	0.03	0.29	0.04	1.90	0.05019
Lpar6	1.2	0.08	1.81	0.16	1.53	0.01576
Lpcat2	0.2	0.05	0.50	0.04	2.23	0.01357
Lpcat3	23.2	1.74	19.13	1.04	-1.21	0.09794
Lpp	2.8	0.25	3.60	0.13	1.32	0.02394
Lpxn	0.2	0.03	0.48	0.07	2.00	0.06573
Lrg1	33.3	1.66	27.16	1.56	-1.23	0.05198

Lrp1	37.1	1.80	56.47	2.31	1.52	0.00039
Lrp8	0.5	0.03	2.17	0.61	3.49	0.01996
Lrrc15	2.8	0.21	1.78	0.09	-1.59	0.00251
Lrrc16a	2.0	0.12	3.01	0.24	1.44	0.02937
Lrrc17	1.0	0.09	1.64	0.18	1.56	0.03602
Lrrc24	27.4	0.77	22.39	0.76	-1.23	0.01121
Lrrc25	1.7	0.07	2.46	0.25	1.38	0.07222
Lrrc32	4.3	0.49	7.49	0.63	1.77	0.00299
Lrrc39	21.6	0.74	16.82	0.47	-1.28	0.00358
Lrrc71	2.5	0.12	5.07	0.77	1.83	0.04413
Lrrc75a	1.6	0.13	2.10	0.14	1.30	0.09813
Lrrc8c	8.1	0.68	11.47	0.65	1.44	0.01090
Lrrcc1	1.5	0.19	2.96	0.46	2.03	0.00766
Lrrk1	4.0	0.32	5.07	0.25	1.26	0.06585
Lsamp	0.3	0.03	0.15	0.03	-2.33	0.01576
Lst1	3.5	0.36	5.38	0.39	1.53	0.06174
Ltbp1	14.2	0.61	11.52	0.61	-1.23	0.04130
Ltbp2	1.3	0.13	5.37	1.04	4.18	0.00140
Ltbp4	93.1	7.26	137.06	14.04	1.46	0.02282
Lum	12.3	0.65	22.48	1.19	1.82	0.00008
Luzp1	5.9	0.25	7.20	0.07	1.23	0.00997
Ly6a	83.5	7.88	113.22	6.74	1.37	0.02750
Ly6e	135.2	4.30	183.59	6.97	1.36	0.00144
Ly86	3.0	0.36	4.50	0.32	1.46	0.05732
Lyl1	2.5	0.19	3.46	0.24	1.37	0.03271
Lypd2	2.8	0.28	0.96	0.38	-4.21	0.02563
Lysmd4	4.5	0.27	3.68	0.26	-1.26	0.06978
Lyve1	4.7	0.31	6.03	0.40	1.26	0.06834
Lyz2	60.1	4.88	100.17	7.39	1.67	0.00149
Lzts1	1.5	0.08	1.14	0.06	-1.26	0.07534
Macrod1	120.0	2.71	87.83	3.88	-1.37	0.00091
Mad2l1	1.3	0.09	0.91	0.07	-1.48	0.05366
Maf	2.2	0.25	3.14	0.37	1.43	0.06694
Maf1	54.0	3.47	41.45	1.09	-1.30	0.01431
Maff	1.8	0.28	2.46	0.15	1.45	0.06931
Maged2	8.0	0.37	9.89	0.55	1.24	0.04211
Magi2	4.6	0.21	3.72	0.20	-1.26	0.04677
Magix	12.9	0.38	10.79	0.70	-1.21	0.05516
Maml2	1.8	0.04	2.52	0.11	1.38	0.00205
Mamstr	4.1	0.18	3.30	0.30	-1.26	0.08109
Man2b2	6.3	0.29	8.29	0.32	1.30	0.00764
Manf	62.5	5.36	30.96	2.00	-1.96	0.00037
Maob	17.5	0.68	11.52	0.75	-1.53	0.00097
Map1a	1.0	0.11	2.07	0.26	2.00	0.00520
Map1lc3a	431.1	17.35	301.91	9.30	-1.43	0.00054
Map1lc3b	134.7	4.55	-162.86	612.64	-1.21	0.01425

Map2	0.7	0.05	1.04	0.06	1.39	0.05952
Map3k12	5.8	0.15	7.29	0.35	1.24	0.05368
Map4	101.2	2.00	122.08	3.71	1.20	0.01114
Map4k2	48.3	2.16	39.79	2.72	-1.23	0.06268
Map4k4	49.6	1.56	64.50	2.10	1.30	0.00269
Map6	1.7	0.10	2.45	0.20	1.47	0.01655
Mapk1ip1	15.5	0.80	11.96	0.91	-1.32	0.03012
Mapkapk5	50.8	3.47	34.26	8.55	-1.68	0.08129
March1	0.2	0.01	0.39	0.05	1.80	0.01328
March9	1.3	0.07	0.94	0.06	-1.34	0.04201
Marcksl1	2.2	0.15	4.52	0.37	1.96	0.00060
Masp1	2.2	0.08	3.70	0.20	1.66	0.00052
Matn2	2.7	0.13	3.87	0.21	1.39	0.00438
Max	16.1	0.85	12.55	0.88	-1.28	0.03707
Mbd6	16.3	0.71	13.48	0.59	-1.21	0.04507
Mcam	13.6	0.50	19.92	1.04	1.45	0.00097
Mcat	9.7	0.42	7.74	0.23	-1.26	0.01327
Mcc	2.5	0.18	3.49	0.15	1.45	0.00429
Mccc1	33.0	0.93	26.73	0.93	-1.24	0.00948
Mccc2	18.4	0.51	13.94	0.70	-1.33	0.00389
Mcee	46.6	2.46	37.93	1.53	-1.23	0.03235
Mcrs1	13.8	0.77	10.99	0.57	-1.27	0.02963
Mdh1	1301.1	35.95	1076.30	19.27	-1.21	0.00837
Mdh2	1231.8	44.84	977.48	11.03	-1.26	0.00303
Me2	2.1	0.13	3.05	0.22	1.40	0.02275
Me3	47.2	1.12	38.04	1.19	-1.24	0.00562
Med26	3.1	0.18	2.14	0.16	-1.41	0.01380
Med7	6.2	0.44	4.71	0.29	-1.31	0.09595
Medag	4.3	0.43	5.81	0.31	1.38	0.02667
Megf10	0.0	0.00	0.06	0.01	2.52	0.00940
Megf9	1.7	0.06	2.06	0.07	1.23	0.02601
Meis2	19.1	0.51	15.45	0.83	-1.23	0.02958
Meox1	4.1	0.63	14.21	1.15	3.47	0.00003
Meox2	6.4	0.26	5.05	0.21	-1.27	0.01860
Mertk	1.3	0.10	1.96	0.17	1.53	0.01053
Mest	2.9	0.57	1.61	0.12	-1.81	0.03099
Met	0.2	0.02	0.27	0.03	1.67	0.03618
Metap1d	18.4	0.83	14.43	0.72	-1.29	0.01666
Mettl11b	0.4	0.04	0.67	0.11	1.79	0.07265
Mettl20	11.1	0.97	8.19	0.65	-1.33	0.06153
Mettl23	26.6	1.10	22.53	1.39	-1.20	0.07800
Mfap2	2.4	0.23	5.82	0.41	2.43	0.00031
Mfap4	7.0	0.65	22.81	2.55	3.19	0.00009
Mfap5	24.2	2.72	38.96	2.14	1.63	0.00278
Mgat4a	2.2	0.18	1.64	0.10	-1.36	0.02276
Mgat4b	30.7	0.71	24.85	1.60	-1.24	0.02899

Mgat5b	0.4	0.17	2.13	0.59	7.50	0.01540
Mgme1	4.2	0.15	3.30	0.19	-1.26	0.03421
Mgp	180.6	14.66	277.96	16.76	1.55	0.00282
Mgst3	170.1	6.89	131.37	4.38	-1.29	0.00413
Mical2	33.0	0.73	41.39	1.32	1.25	0.00481
Micu1	8.4	0.25	10.16	0.38	1.21	0.03898
Mid1	2.3	0.11	3.82	0.25	1.61	0.00175
Mill2	2.0	0.14	2.80	0.23	1.42	0.04547
Mipep	19.9	0.72	16.35	0.41	-1.22	0.01256
Mir17hg	0.3	0.03	0.16	0.02	-1.59	0.08697
Mir208b	1315.0	183.21	739.07	110.21	-1.75	0.08702
Mir8112	205.5	18.51	118.22	29.12	-2.01	0.02264
Mkl1	3.9	0.09	4.75	0.20	1.20	0.04888
Mks1	2.7	0.15	2.06	0.07	-1.31	0.06605
Mllt11	5.4	0.37	7.82	0.43	1.45	0.00671
Mllt4	38.8	2.51	28.02	1.42	-1.39	0.00612
Mllt6	28.3	1.05	23.29	1.02	-1.22	0.03193
Mlycd	33.2	2.22	22.80	0.34	-1.45	0.00101
Mmd	10.8	0.25	7.60	0.41	-1.42	0.00107
Mme	1.7	0.11	1.24	0.19	-1.48	0.06821
Mmgt2	3.0	0.21	4.10	0.18	1.36	0.05198
Mmp15	19.4	1.25	14.88	0.88	-1.30	0.03198
Mmp19	0.3	0.04	0.37	0.03	1.54	0.07168
Mmp2	15.3	1.13	22.33	1.09	1.46	0.00407
Mmp23	5.1	0.32	7.41	0.58	1.47	0.01995
Mmp28	1.3	0.11	1.85	0.15	1.37	0.04932
Mmp3	1.6	0.29	4.12	0.12	2.88	0.00016
Mmrn2	10.5	0.29	13.44	0.34	1.28	0.00299
Mn1	2.8	0.27	3.77	0.27	1.35	0.05757
Mob3b	0.1	0.00	0.16	0.02	1.65	0.04671
Mocs3	1.7	0.10	1.26	0.09	-1.43	0.02058
Mov10l1	55.9	2.03	42.83	3.27	-1.31	0.01779
Mpc2	160.5	6.09	98.57	4.57	-1.63	0.00011
Mpeg1	1.5	0.13	3.05	0.34	1.91	0.00098
Mpnd	33.9	1.16	27.90	1.13	-1.22	0.02394
Mpped2	9.4	0.41	6.78	0.50	-1.38	0.01295
Mpv17	44.9	2.24	35.80	1.87	-1.25	0.03618
Mrc1	3.8	0.33	6.43	0.28	1.67	0.00079
Mrc2	4.9	0.30	7.31	0.40	1.50	0.00184
Mrgpre	1.2	0.13	1.51	0.08	1.34	0.06604
Mrm1	5.7	0.28	4.26	0.12	-1.34	0.00546
Mrpl14	92.3	3.21	67.43	2.85	-1.38	0.00155
Mrpl34	77.3	2.62	61.95	2.88	-1.25	0.01646
Mrpl38	33.5	0.68	27.77	1.55	-1.22	0.03104
Mrpl39	21.4	0.64	17.46	0.70	-1.23	0.01406
Mrpl42	355.9	21.06	292.01	14.88	-1.22	0.06450

Mrpl48	69.8	3.42	56.47	1.66	-1.23	0.02260
Mrpl52	70.6	2.13	56.37	2.15	-1.25	0.01431
Mrps15	47.5	1.73	36.16	1.12	-1.32	0.00257
Mrps21	200.6	4.97	159.06	4.19	-1.26	0.00313
Mrps24	70.3	3.25	48.86	1.64	-1.44	0.00076
Mrps26	17.5	0.66	13.27	0.11	-1.31	0.00243
Mrps28	34.5	0.76	25.41	1.11	-1.36	0.00196
Mrps34	39.4	2.71	22.83	1.15	-1.74	0.00048
Mrps35	44.4	1.92	34.49	0.27	-1.28	0.00363
Mrvi1	3.5	0.20	4.45	0.17	1.30	0.01806
Ms4a14	0.1	0.02	0.28	0.03	1.89	0.03288
Ms4a4d	4.9	0.24	6.69	0.29	1.32	0.01200
Ms4a6b	2.4	0.27	4.24	0.33	1.68	0.01184
Ms4a6d	1.2	0.16	2.18	0.28	1.83	0.02281
Ms4a7	1.6	0.19	3.03	0.25	1.85	0.00760
Msmo1	2.8	0.17	3.67	0.27	1.29	0.08773
Msn	32.4	1.01	44.50	1.34	1.37	0.00073
Msrb2	32.4	0.90	26.99	0.80	-1.20	0.01614
Msx1	1.6	0.14	2.61	0.22	1.60	0.02133
Mt2	46.9	13.68	69.92	3.11	1.77	0.08193
mt-Co2	36414.4	4985.53	19533.60	3472.77	-1.90	0.02348
mt-Co3	0.0	0.00	0.00	0.00	-1.54	0.05646
Mtg2	13.8	0.39	11.11	0.23	-1.25	0.00620
Mthfd2	1.9	0.06	3.36	0.45	1.68	0.01638
Mtl5	1.4	0.12	0.81	0.06	-1.58	0.01296
Mtmr10	1.8	0.14	2.23	0.10	1.26	0.07484
Mtmr12	2.1	0.10	2.67	0.09	1.25	0.03710
Mtr	7.0	0.48	4.65	0.36	-1.51	0.00498
mt-Tq	175851.0	17460.54	130393.48	4838.47	-1.32	0.05217
mt-Tv	4635.6	310.26	2452.15	436.88	-2.03	0.01114
Mum1	6.5	0.25	5.52	0.28	-1.20	0.06856
Mut	16.1	0.60	12.93	0.34	-1.25	0.00749
Mxra7	7.6	0.46	13.17	1.01	1.72	0.00098
Mxra8	17.3	1.39	22.16	1.19	1.27	0.06552
Mybpc2	5.9	0.28	10.84	1.23	1.80	0.00206
Myc	1.7	0.15	2.65	0.16	1.59	0.01212
Myh10	7.1	0.42	9.22	0.45	1.30	0.02079
Myh7	484.1	81.55	1770.33	861.49	2.70	0.04333
Myh9	22.2	1.47	32.17	0.60	1.46	0.00147
Myl1	22.2	1.55	42.42	8.70	1.78	0.02875
Myl2	13731.2	541.60	11205.97	358.20	-1.23	0.01650
Myl9	28.4	1.09	35.94	2.76	1.25	0.06155
Mylip	5.0	0.25	3.52	0.26	-1.44	0.00639
Mylk	2.8	0.13	3.48	0.13	1.24	0.02394
Myo1f	1.6	0.15	2.72	0.13	1.65	0.00434
Myo1g	0.9	0.07	1.38	0.11	1.44	0.04428

Myo5a	2.1	0.06	3.56	0.23	1.64	0.00070
Myo5c	1.2	0.06	0.78	0.07	-1.54	0.01337
Myo9b	6.0	0.21	7.29	0.29	1.21	0.02807
Myof	1.0	0.05	1.91	0.17	1.81	0.00110
Myot	21.1	1.69	49.45	4.33	2.34	0.00006
N4bp2os	1.1	0.14	0.60	0.07	-1.79	0.03876
Naa38	39.7	1.26	31.05	0.83	-1.28	0.01026
Naalad2	1.6	0.08	2.11	0.09	1.33	0.01448
Nab1	5.6	0.23	6.92	0.45	1.24	0.05794
Nabp1	13.7	0.91	19.30	2.67	1.38	0.06722
Nadk2	20.2	1.22	14.44	0.39	-1.40	0.00197
Naip2	0.2	0.02	0.34	0.03	1.48	0.08162
Nalcn	0.1	0.02	0.20	0.03	1.96	0.05119
Nampt	26.6	2.00	21.21	0.66	-1.24	0.05606
Napepld	1.5	0.13	2.07	0.11	1.40	0.01864
Naprt	5.4	0.33	4.38	0.20	-1.25	0.06864
Nav1	7.7	0.24	9.62	0.41	1.24	0.01295
Nbeal2	9.8	0.46	8.03	0.21	-1.23	0.02251
Nbn	6.9	0.21	5.32	0.17	-1.30	0.00498
Ncf1	2.0	0.20	3.17	0.14	1.54	0.01360
Ncf2	1.4	0.17	1.88	0.12	1.35	0.08557
Nckap1l	1.1	0.06	1.92	0.10	1.73	0.00037
Nckap5l	3.3	0.20	4.74	0.25	1.44	0.00515
Ncoa7	0.5	0.05	0.83	0.06	1.55	0.01732
Ndfip1	46.6	3.14	35.71	1.32	-1.30	0.01428
Ndnl2	0.8	0.07	0.64	0.03	-1.28	0.07619
Ndufa1	372.0	17.90	293.89	8.20	-1.26	0.00969
Ndufa10	959.5	36.03	766.67	18.31	-1.25	0.00605
Ndufa12	557.9	22.55	439.90	8.68	-1.27	0.00470
Ndufa13	166.2	5.75	133.80	2.97	-1.24	0.00620
Ndufa2	262.1	8.46	204.59	9.46	-1.29	0.00682
Ndufa3	1559.6	78.44	1170.00	46.47	-1.33	0.00469
Ndufa4	1698.8	109.48	1308.66	19.28	-1.29	0.00992
Ndufa5	851.4	40.94	672.51	11.16	-1.26	0.00672
Ndufa6	411.3	16.76	332.37	7.04	-1.24	0.00962
Ndufa7	382.1	16.53	303.21	6.99	-1.26	0.00717
Ndufa8	250.2	10.48	198.28	8.04	-1.26	0.01162
Ndufa9	211.9	7.79	172.52	3.03	-1.23	0.00755
Ndufb10	522.0	22.00	406.13	16.75	-1.29	0.00768
Ndufb11	274.7	10.04	211.41	7.22	-1.30	0.00337
Ndufb4	121.2	5.35	97.36	4.66	-1.24	0.02291
Ndufb6	139.9	5.61	114.60	3.47	-1.22	0.01800
Ndufb7	418.5	7.90	343.11	8.49	-1.22	0.00574
Ndufb8	595.3	8.80	478.88	13.76	-1.25	0.00344
Ndufb9	792.5	21.01	653.15	19.14	-1.21	0.01021
Ndufc1	749.7	36.58	575.53	11.86	-1.30	0.00446

Ndufc2	177.9	8.18	144.97	4.35	-1.22	0.02219
Ndufs1	321.3	6.98	265.04	9.66	-1.22	0.01098
Ndufs2	716.5	25.94	567.13	17.55	-1.26	0.00556
Ndufs3	242.8	4.77	194.47	3.38	-1.25	0.00227
Ndufs5	248.2	10.46	194.42	5.46	-1.27	0.00568
Ndufs6	470.2	16.47	381.28	6.74	-1.23	0.00677
Ndufs7	528.9	20.35	419.28	11.00	-1.26	0.00539
Ndufs8	194.1	5.28	152.62	4.28	-1.27	0.00283
Ndufv1	459.2	22.73	346.59	9.02	-1.32	0.00361
Ndufv2	459.4	20.14	374.31	8.84	-1.23	0.01425
Ndufv3	634.7	18.98	511.83	11.18	-1.24	0.00506
Nedd9	5.5	0.38	7.64	0.68	1.39	0.02531
Negr1	0.2	0.03	0.45	0.06	2.04	0.01245
Nenf	58.3	2.24	48.11	2.02	-1.21	0.03065
Nepn	1.2	0.15	0.30	0.03	-3.66	0.00054
Nes	5.3	0.30	9.34	0.73	1.76	0.00056
Neurl2	15.9	0.54	12.92	0.66	-1.23	0.04128
Nfam1	0.6	0.06	0.96	0.05	1.75	0.00500
Nfatc4	1.3	0.08	1.86	0.08	1.43	0.00612
Nfil3	3.7	0.28	4.61	0.10	1.27	0.04650
Nfkbie	0.6	0.06	0.87	0.06	1.61	0.03153
Ngfr	0.2	0.03	0.36	0.08	2.19	0.05266
Nhs	0.1	0.02	0.22	0.01	1.86	0.03928
Nhsl2	0.6	0.03	0.96	0.11	1.58	0.01535
Nid1	17.7	0.66	22.04	1.13	1.24	0.02117
Nid2	3.8	0.39	5.65	0.27	1.46	0.01058
Nkx2-5	36.4	1.21	27.71	1.22	-1.32	0.00350
Nle1	8.9	0.35	7.13	0.62	-1.26	0.09967
Nlgn2	1.7	0.06	2.15	0.18	1.25	0.08390
Nlrc3	1.1	0.08	4.95	1.32	4.19	0.00430
Nlrc5	1.1	0.17	2.31	0.25	2.02	0.01107
Nlrp1b	0.1	0.01	0.25	0.03	2.00	0.01727
Nme2	299.4	11.93	219.89	10.44	-1.35	0.00529
Nnt	96.5	3.20	76.72	3.48	-1.26	0.00999
Nos1	0.1	0.01	0.20	0.04	2.64	0.02506
Nos1ap	6.5	0.47	5.15	0.31	-1.29	0.09592
Nos2	3.3	0.29	1.75	0.10	-1.89	0.00048
Nos3	9.4	0.60	6.77	0.41	-1.41	0.00612
Nostrin	2.9	0.29	3.61	0.03	1.29	0.09705
Notch3	7.2	0.35	12.42	0.81	1.73	0.00022
Nox4	1.3	0.19	3.12	0.28	2.46	0.00257
Npc1	7.8	0.37	6.11	0.40	-1.29	0.02804
Npdc1	22.3	0.59	26.79	0.99	1.20	0.03069
Nppa	161.2	64.31	623.78	201.04	3.85	0.02492
Nppb	211.4	46.83	566.16	96.01	2.77	0.00701
Npr3	1.3	0.12	1.88	0.25	1.47	0.06335

Nr0b2	9.5	0.62	7.41	0.68	-1.32	0.05603
Nr2f1	1.7	0.09	1.08	0.12	-1.61	0.01854
Nr2f6	29.1	0.97	21.04	1.23	-1.40	0.00206
Nr5a2	0.5	0.08	1.29	0.08	2.78	0.00071
Nrap	128.7	4.75	169.40	9.31	1.31	0.00664
Nrp2	7.3	0.65	5.57	0.24	-1.29	0.06250
Nrros	3.4	0.14	4.63	0.30	1.33	0.02577
Nrtn	10.5	0.70	6.10	0.58	-1.75	0.00211
Nrxn1	1.5	0.30	0.47	0.07	-2.86	0.00764
Ntf3	1.8	0.12	1.14	0.12	-1.64	0.01830
Nuak1	7.6	0.43	12.29	0.92	1.61	0.00107
Nudc	47.1	1.62	39.43	2.90	-1.20	0.09266
Nudt6	20.5	1.46	14.66	1.14	-1.45	0.03909
Nudt8	82.4	1.78	64.80	1.51	-1.27	0.00196
Numb	10.8	0.41	13.58	0.20	1.26	0.00488
Numbl	2.1	0.09	2.80	0.22	1.29	0.05118
Nup210	3.0	0.22	1.93	0.09	-1.59	0.00435
Nupr1	6.1	0.46	8.24	0.53	1.34	0.05302
Nxn	3.2	0.19	2.47	0.29	-1.36	0.04276
Nxpe3	0.1	0.01	0.18	0.01	1.66	0.03158
Oas1a	1.0	0.11	1.60	0.10	1.67	0.02566
Oasl2	6.2	0.40	7.81	0.39	1.26	0.05031
Oaz1	288.6	6.10	233.82	4.93	-1.24	0.00373
Olfml1	1.4	0.16	2.19	0.25	1.53	0.05864
Olfml2b	2.2	0.20	3.70	0.22	1.69	0.00176
Olfml3	3.0	0.13	4.72	0.24	1.54	0.00054
Olfr1396	1.2	0.10	3.59	0.39	2.78	0.00078
Olfr558	1.2	0.07	1.53	0.08	1.32	0.07028
Oma1	16.1	0.49	12.77	0.29	-1.27	0.00507
Opcml	3.5	0.28	1.78	0.09	-1.89	0.00420
Ophn1	5.4	0.27	7.21	0.45	1.28	0.04156
Oplah	21.6	1.27	16.49	0.06	-1.30	0.00513
Opn4	0.7	0.09	0.45	0.04	-1.52	0.08505
Orai2	0.5	0.06	0.86	0.07	1.82	0.00731
Osbpl5	6.7	0.49	8.81	0.40	1.31	0.02611
Osbpl6	5.3	0.40	3.70	0.48	-1.46	0.04641
Osgep	19.7	0.54	15.96	0.36	-1.24	0.00676
Oxld1	20.9	1.08	14.91	0.89	-1.40	0.00654
P2rx7	1.3	0.10	2.28	0.17	1.78	0.00239
P2ry14	3.0	0.12	1.96	0.06	-1.49	0.00251
P2ry2	10.9	0.53	13.88	0.51	1.27	0.01326
P2ry6	2.4	0.24	4.40	0.28	1.77	0.00244
P3h2	3.7	0.55	6.79	0.78	1.88	0.01385
P3h3	4.9	0.27	6.45	0.28	1.33	0.01351
P3h4	13.6	0.72	6.75	0.48	-2.07	0.00005
Pabpn1	35.8	1.55	27.74	1.41	-1.27	0.02890

Padi2	0.3	0.04	0.63	0.07	1.96	0.01394
Pak1	0.3	0.05	0.55	0.04	1.60	0.06291
Pald1	4.2	0.24	3.39	0.09	-1.24	0.03340
Palld	46.3	1.59	38.10	1.94	-1.22	0.03591
Pamr1	0.6	0.03	2.07	0.28	3.45	0.00108
Pank1	8.1	0.74	5.52	0.11	-1.44	0.00530
Panx1	1.3	0.14	2.29	0.27	1.64	0.05684
Papln	3.6	0.31	5.77	0.36	1.58	0.00397
Paqr7	5.9	0.25	8.72	0.39	1.47	0.00114
Paqr9	4.7	0.14	3.27	0.21	-1.43	0.00181
Parp9	4.5	0.31	5.73	0.22	1.28	0.02798
Pbld2	0.9	0.11	0.54	0.06	-1.55	0.05264
Pcbd2	15.4	0.52	12.54	0.67	-1.23	0.09498
Pccb	56.0	1.80	43.33	1.28	-1.29	0.00258
Pcdh1	10.7	0.56	13.28	0.57	1.23	0.03287
Pcdh12	7.0	0.81	4.08	0.35	-1.75	0.00438
Pcdh18	1.1	0.05	1.72	0.13	1.56	0.00506
Pcdh9	0.7	0.11	1.08	0.09	1.78	0.01821
Pcdhb16	0.2	0.02	0.36	0.03	1.72	0.01847
Pcdhb22	0.3	0.02	0.46	0.02	1.50	0.01252
Pcdhga2	1.8	0.08	1.50	0.09	-1.26	0.08436
Pcdhgb7	1.3	0.04	0.94	0.10	-1.40	0.04488
Pcna	11.5	0.75	9.25	0.44	-1.24	0.06273
Pcnt	7.2	0.50	5.89	0.15	-1.21	0.06234
Pcnx	5.5	0.19	6.98	0.16	1.26	0.00467
Pcolce	31.3	1.58	50.62	1.23	1.62	0.00008
Pcolce2	6.6	0.37	9.45	0.59	1.42	0.00760
Pcp4l1	110.0	6.29	78.30	5.71	-1.41	0.00876
Pcsk4	1.7	0.08	1.09	0.08	-1.51	0.02044
Pcsk5	0.4	0.02	0.62	0.03	1.45	0.01118
Pdcd5	79.7	4.46	62.70	2.42	-1.26	0.02034
Pde1a	1.9	0.08	2.69	0.07	1.44	0.00350
Pde8a	4.8	0.14	6.75	0.38	1.39	0.00337
Pdgfb	13.5	0.34	22.13	1.21	1.64	0.00016
Pdgfd	2.8	0.19	4.17	0.07	1.55	0.00070
Pdgfra	5.9	0.37	7.40	0.60	1.25	0.08259
Pdgfrb	13.6	0.66	19.41	1.03	1.42	0.00211
Pdha1	235.2	8.33	195.98	6.07	-1.20	0.02237
Pdia3	64.9	3.05	53.47	0.72	-1.21	0.02175
Pdia4	11.8	0.98	8.72	0.56	-1.32	0.03899
Pdia6	25.5	1.80	18.96	0.54	-1.33	0.01171
Pdk2	215.7	10.68	175.94	6.80	-1.22	0.03524
Pdlim2	6.4	0.50	12.09	1.47	1.81	0.01657
Pdlim3	6.3	0.45	8.60	0.33	1.34	0.01411
Pdlim5	277.5	12.40	339.55	17.87	1.22	0.04632
Pdp2	3.9	0.14	2.48	0.18	-1.56	0.00106

Pdpn	1.7	0.06	2.64	0.12	1.51	0.00257
Pdzd2	14.2	0.56	11.39	0.96	-1.26	0.05328
Pdzd3	1.0	0.10	1.82	0.26	1.79	0.03445
Pdzrn4	0.4	0.08	0.18	0.05	-2.33	0.05455
Pea15a	29.0	0.96	36.43	1.22	1.25	0.00682
Pear1	12.6	0.85	15.52	0.71	1.23	0.06944
Pecam1	40.4	1.39	52.18	2.41	1.29	0.00725
Peg10	0.3	0.04	0.57	0.02	1.86	0.01382
Pemt	2.2	0.31	3.53	0.27	1.72	0.05784
Penk	3.7	0.41	1.20	0.25	-3.58	0.00087
Pex11a	7.5	0.43	5.58	0.15	-1.32	0.01076
Pex6	12.9	0.26	10.25	0.14	-1.26	0.00227
Pex7	17.2	0.65	13.46	0.57	-1.28	0.01196
Pf4	27.2	2.48	37.00	1.05	1.33	0.03475
Pfas	3.1	0.22	2.39	0.19	-1.28	0.07677
Pfkfb1	5.4	0.71	1.66	0.25	-3.37	0.00040
Pfkfb4	1.4	0.06	2.16	0.24	1.49	0.04181
Pfkl	39.8	0.89	32.91	0.98	-1.21	0.01049
Pgap2	44.5	2.41	53.72	1.48	1.22	0.03356
Pgf	2.3	0.28	1.10	0.09	-2.13	0.00186
Pgls	38.1	0.73	27.82	0.49	-1.37	0.00032
Pgp	19.0	0.47	12.22	0.83	-1.57	0.00071
Phlda3	4.4	0.24	6.17	0.70	1.36	0.09946
Phldb2	5.8	0.38	7.31	0.43	1.25	0.06170
Phxr4	1.5	0.15	2.12	0.06	1.45	0.03745
Phyh	281.6	12.48	232.25	7.22	-1.21	0.02377
Pianp	0.7	0.12	1.35	0.23	1.85	0.06810
Picalm	20.4	0.86	25.03	0.98	1.23	0.02392
Pid1	4.3	0.41	6.69	0.46	1.53	0.00869
Piezo1	9.1	0.42	10.95	3.83	1.20	0.04907
Piezo2	0.1	0.02	0.20	0.02	1.98	0.01725
Pigw	0.8	0.05	1.03	0.07	1.34	0.07514
Pigyl	17.9	0.33	14.63	0.34	-1.23	0.01847
Pik3ap1	0.7	0.08	1.14	0.10	1.49	0.05812
Pik3c2a	0.8	0.07	1.09	0.03	1.38	0.01367
Pik3c2b	6.1	0.17	4.95	0.14	-1.23	0.00899
Pik3cb	2.4	0.06	2.02	0.10	-1.21	0.05211
Pik3cd	2.1	0.10	3.01	0.21	1.36	0.02164
Pik3cg	0.3	0.02	0.47	0.03	1.57	0.01300
Pik3r1	13.4	0.98	10.75	0.62	-1.24	0.08729
Pik3r3	1.6	0.13	2.87	0.13	1.83	0.00039
Pik3r6	5.5	0.24	3.95	0.19	-1.40	0.00707
Pilra	1.5	0.30	2.99	0.31	1.98	0.03370
Pim3	23.6	1.86	13.78	0.59	-1.70	0.00069
Pink1	195.6	8.69	142.72	2.26	-1.37	0.00103
Pip4k2a	4.4	0.16	6.72	0.30	1.49	0.00088

Pirb	1.3	0.05	1.87	0.13	1.39	0.02226
Pkn3	7.2	0.54	10.44	0.24	1.43	0.00265
Pla2g4a	2.8	0.28	4.57	0.40	1.59	0.02778
Pla2g5	13.3	1.33	7.87	0.76	-1.70	0.00783
Pla2r1	0.8	0.10	1.28	0.03	1.73	0.00755
Plbd1	18.1	0.94	11.24	0.63	-1.63	0.00050
Plcb1	2.6	0.30	4.08	0.15	1.63	0.00186
Plcb2	1.2	0.08	0.87	0.07	-1.37	0.07788
Plcd1	7.3	0.47	6.04	0.18	-1.21	0.06214
Plcg2	1.9	0.03	2.65	0.13	1.40	0.00254
Plcl1	0.4	0.03	0.51	0.02	1.45	0.01401
Pld4	3.4	0.21	5.86	0.66	1.67	0.00386
Plek	0.6	0.05	1.03	0.12	1.65	0.01920
Plekha4	2.6	0.16	4.29	0.25	1.66	0.00256
Plekhf1	9.7	0.58	7.32	0.41	-1.34	0.01668
Plekhg2	11.4	0.42	14.08	0.83	1.23	0.04525
Plekhh3	9.8	0.47	7.73	0.31	-1.27	0.01687
Plekho1	46.9	1.04	63.74	3.43	1.35	0.00361
Plekho2	4.1	0.22	5.07	0.27	1.23	0.05650
Plin4	48.3	5.17	32.35	2.84	-1.49	0.01949
Plin5	43.0	4.34	32.55	1.27	-1.30	0.05846
Plk2	9.4	0.69	5.16	0.38	-1.80	0.00076
Plk5	0.4	0.06	0.16	0.02	-2.51	0.00702
Plp2	21.7	0.85	27.49	0.72	1.26	0.00950
Pls3	9.9	0.27	14.17	0.58	1.42	0.00051
Plscr2	8.3	0.31	11.53	0.46	1.37	0.00270
Plvap	4.2	0.25	6.17	0.59	1.47	0.01416
Plxdc2	6.7	0.22	5.45	0.23	-1.23	0.02804
Plxna3	1.0	0.13	1.72	0.22	1.68	0.03520
Plxnb1	13.1	0.75	9.43	0.80	-1.41	0.01826
Plxnc1	0.2	0.02	0.41	0.04	1.76	0.00563
Pmepa1	17.5	1.01	25.68	1.74	1.46	0.00402
Pmp22	20.2	0.91	29.56	0.52	1.46	0.00032
Pnkd	56.1	1.71	46.32	2.64	-1.22	0.04201
Pnpla2	260.8	21.86	181.59	9.00	-1.42	0.00809
Podxl	30.1	1.29	39.67	1.54	1.32	0.00407
Podxl2	5.4	0.36	3.53	0.37	-1.56	0.01215
Pola2	3.1	0.11	2.54	0.25	-1.27	0.07983
Pold1	5.7	0.26	4.40	0.05	-1.30	0.00743
Pold2	22.7	1.40	18.39	1.18	-1.25	0.06276
Polk	0.7	0.05	1.08	0.02	1.47	0.00603
Polr2f	68.8	0.83	54.91	1.68	-1.25	0.00437
Polr3g	3.2	0.21	4.36	0.30	1.32	0.07191
Pop5	19.3	1.00	14.24	0.75	-1.31	0.02157
Popdc3	10.0	0.74	12.17	0.25	1.24	0.04490
Postn	8.9	0.35	33.33	4.63	3.58	0.00004

Pou2f2	0.4	0.03	0.62	0.02	1.72	0.00231
Ppap2a	31.7	1.75	40.01	1.65	1.26	0.02473
Ppip5k2	22.0	1.27	15.06	0.89	-1.46	0.00267
Ppm1d	2.4	0.10	1.76	0.07	-1.40	0.00397
Ppm1f	6.7	0.24	8.10	0.40	1.21	0.04429
Ppm1k	9.0	0.45	7.25	0.42	-1.24	0.04413
Ppp1r13l	15.4	1.08	11.32	0.64	-1.36	0.01324
Ppp1r18	6.2	0.32	7.54	0.45	1.23	0.07073
Ppp1r26	0.8	0.06	0.58	0.06	-1.42	0.04936
Ppp1r3c	30.4	1.75	43.10	2.01	1.42	0.00235
Ppp1r9a	2.3	0.12	1.94	0.08	-1.20	0.06660
Ppp2r3d	14.1	0.45	9.75	0.45	-1.46	0.00091
Pqlc3	2.3	0.12	3.03	0.16	1.34	0.02689
Pradc1	5.8	0.26	4.46	0.29	-1.29	0.05886
Prelp	15.7	0.84	26.95	0.96	1.71	0.00008
Prickle2	1.1	0.08	1.93	0.16	1.78	0.00152
Prkab1	27.7	1.70	22.24	1.26	-1.25	0.05285
Prkag3	0.9	0.06	1.32	0.13	1.55	0.03142
Prkar1b	1.3	0.06	2.29	0.20	1.78	0.00556
Prkcb	1.6	0.14	3.25	0.23	2.05	0.00233
Prkcd	6.0	0.23	7.60	0.38	1.26	0.02679
Prkcdbp	15.6	0.75	20.29	1.04	1.29	0.02108
Prkd1	1.0	0.08	0.71	0.08	-1.46	0.06750
Prlr	1.4	0.12	2.19	0.22	1.57	0.01033
Procr	0.7	0.11	1.23	0.13	1.64	0.08161
Prorsd1	7.0	0.37	8.40	0.23	1.23	0.05952
Pros1	4.0	0.22	5.41	0.29	1.34	0.01185
Prr33	12.1	0.17	9.67	0.60	-1.27	0.02212
Prr5l	0.2	0.02	0.32	0.03	1.74	0.04629
Prrg3	2.4	0.11	3.83	0.22	1.56	0.00097
Prss23	1.6	0.20	2.90	0.27	1.86	0.00649
Prune2	12.6	1.06	16.77	1.09	1.33	0.03099
Psd4	0.6	0.06	0.92	0.04	1.60	0.02842
Psmb4	104.2	2.39	84.98	2.69	-1.23	0.00760
Psmb9	7.1	0.68	9.14	0.38	1.30	0.09372
Psme1	117.7	5.76	90.37	2.44	-1.30	0.00583
Psmg1	12.7	0.65	10.61	0.38	-1.21	0.05952
Pstpip1	1.5	0.21	2.55	0.26	1.71	0.03051
Ptger1	5.6	0.12	6.89	0.54	1.21	0.09436
Ptgir	1.5	0.13	2.30	0.10	1.54	0.00420
Ptgis	5.0	0.49	7.47	0.60	1.50	0.01666
Ptgs2	0.4	0.06	0.80	0.07	2.23	0.00530
Ptk7	0.8	0.09	1.17	0.10	1.52	0.03959
Ptma	90.9	2.94	115.53	8.51	1.26	0.03928
Ptn	0.3	0.07	0.99	0.12	3.00	0.00209
Ptp4a3	88.9	2.89	60.43	3.17	-1.48	0.00059

Ptpn11	44.5	2.13	54.33	3.12	1.22	0.06278
Ptpn13	0.4	0.02	0.53	0.03	1.39	0.01922
Ptpn3	5.2	0.18	4.27	0.16	-1.21	0.02439
Ptpn6	15.3	0.69	22.48	0.89	1.43	0.00560
Ptpn9	2.8	0.17	3.43	0.11	1.21	0.07434
Ptprc	1.3	0.08	2.27	0.13	1.66	0.00129
Ptprj	1.0	0.09	1.54	0.06	1.50	0.00693
Ptprn	2.0	0.21	2.63	0.16	1.31	0.09144
Ptpro	0.3	0.03	0.55	0.05	1.60	0.02343
Pttg1	109.1	4.46	79.09	4.23	-1.39	0.00283
Pvrl2	10.3	0.89	6.93	0.54	-1.48	0.01067
Pwwp2b	2.6	0.10	3.37	0.08	1.26	0.01655
Pxmp2	89.2	2.59	66.86	2.03	-1.33	0.00136
Pycr1	0.8	0.07	1.39	0.05	1.84	0.00498
Pyurf	9.2	0.37	7.49	0.12	-1.22	0.01576
Qdpr	33.0	0.90	25.96	1.05	-1.26	0.00982
Qsox1	12.1	0.65	17.27	0.86	1.44	0.00267
Qsox2	4.5	0.14	3.21	0.20	-1.42	0.00289
Qtrtd1	3.3	0.22	2.47	0.14	-1.30	0.05970
Rab12	53.3	2.40	44.21	0.92	-1.20	0.02212
Rab27a	0.7	0.08	0.40	0.01	-1.61	0.02745
Rab27b	0.1	0.01	0.17	0.02	2.29	0.00638
Rab31	3.1	0.03	4.02	0.22	1.27	0.02214
Rab33b	4.0	0.21	5.15	0.19	1.28	0.03438
Rab37	0.5	0.08	1.03	0.11	1.93	0.02077
Rab3il1	4.1	0.37	6.33	0.19	1.50	0.00400
Rab7b	1.8	0.13	2.59	0.12	1.39	0.02133
Rab8b	1.3	0.08	1.94	0.14	1.51	0.00437
Rabac1	112.4	1.82	87.01	4.99	-1.30	0.00576
Rac2	0.9	0.07	1.79	0.20	1.74	0.01066
Rad1	3.1	0.16	2.44	0.12	-1.26	0.07197
Rad50	2.2	0.14	2.78	0.19	1.28	0.07146
Rai14	0.8	0.06	1.48	0.03	1.83	0.00017
Ramp1	3.0	0.15	1.61	0.36	-2.12	0.00808
Ramp2	39.0	1.76	31.86	1.66	-1.23	0.03319
Ramp3	2.5	0.11	3.53	0.32	1.37	0.04590
Rap1gap	15.8	0.61	11.57	0.91	-1.37	0.01731
Rap2c	3.5	0.17	4.41	0.31	1.27	0.05669
Rapsn	7.6	0.29	5.93	0.22	-1.31	0.01118
Rasa3	7.2	0.22	8.75	0.34	1.21	0.02798
Rasd2	1.5	0.15	1.14	0.09	-1.34	0.09423
Rasgrp2	38.5	1.31	31.58	1.36	-1.23	0.02065
Rasl12	3.6	0.32	4.69	0.19	1.29	0.05762
Rassf1	5.9	0.16	7.04	0.29	1.20	0.06750
Rassf2	2.5	0.09	3.54	0.39	1.36	0.04562
Rassf5	0.5	0.03	0.69	0.02	1.31	0.08524

Rbfa	18.7	0.34	15.41	0.66	-1.21	0.01949
Rbfox1	13.9	0.65	9.95	0.67	-1.41	0.00529
Rbm24	41.2	2.84	32.35	1.08	-1.27	0.02899
Rbm38	196.4	7.55	162.87	8.14	-1.21	0.03943
Rbm45	5.4	0.33	4.08	0.13	-1.31	0.02377
Rbm47	0.2	0.05	0.41	0.04	1.85	0.05424
Rbms3	1.9	0.20	2.58	0.14	1.40	0.02666
Rbp1	2.9	0.32	4.93	0.61	1.71	0.02542
Rbp7	24.5	4.41	11.48	3.19	-2.34	0.02689
Rcan1	27.9	2.74	41.25	5.53	1.46	0.05455
Rcc2	4.7	0.30	5.91	0.18	1.27	0.03475
Rcn3	10.3	0.76	13.19	0.36	1.28	0.02188
Rdh11	1.1	0.07	1.42	0.07	1.28	0.09960
Rdm1	29.0	1.02	20.63	0.75	-1.43	0.00076
Reck	1.8	0.06	2.30	0.13	1.25	0.04870
Reep1	4.0	0.14	3.30	0.23	-1.20	0.09912
Rem1	7.6	0.48	9.96	0.47	1.29	0.05029
Reps2	0.2	0.01	0.33	0.02	1.65	0.00714
Ret	0.8	0.08	0.42	0.09	-2.11	0.00761
Retnla	3.4	0.47	1.80	0.30	-1.98	0.02638
Retsat	31.9	0.91	20.94	1.33	-1.54	0.00060
Rgcc	20.1	2.15	12.59	1.02	-1.61	0.01249
Rgs10	4.3	0.32	6.63	0.91	1.50	0.06903
Rgs12	4.9	0.14	6.20	0.25	1.24	0.01329
Rgs16	0.4	0.03	1.53	0.20	3.59	0.00032
Rgs2	15.9	1.06	9.73	1.00	-1.62	0.00560
Rgs5	99.3	4.38	137.92	4.47	1.39	0.00107
Rhbdf1	15.5	0.60	18.80	0.37	1.21	0.01629
Rhbdl3	2.0	0.18	1.37	0.12	-1.43	0.03150
Rheb	24.8	0.74	20.14	0.59	-1.23	0.01006
Rhoc	44.4	2.25	53.85	2.78	1.21	0.06026
Rhog	10.8	0.39	13.14	0.96	1.21	0.07808
Rhpn2	1.4	0.09	1.88	0.13	1.34	0.03899
Rilp	24.8	0.47	18.75	0.66	-1.32	0.00152
Rin1	0.5	0.05	0.86	0.08	1.55	0.02870
Rin3	6.1	0.56	4.20	0.20	-1.43	0.01962
Ripply3	1.7	0.12	2.49	0.18	1.44	0.03524
Rit1	31.2	1.09	25.64	1.41	-1.23	0.03593
Rnaset2a	46.6	3.08	28.50	2.02	-1.59	0.01035
Rnaset2b	57.6	6.11	27.68	5.21	-2.20	0.01428
Rnd1	1.9	0.26	2.62	0.15	1.50	0.05995
Rnf122	1.8	0.08	1.29	0.06	-1.45	0.00404
Rnf128	16.8	1.02	13.29	0.69	-1.26	0.03913
Rnf152	0.3	0.02	0.67	0.05	2.16	0.00040
Rnf165	0.4	0.05	0.62	0.10	1.78	0.07016
Rnf187	56.6	0.91	46.49	1.29	-1.22	0.00652

Rnf207	48.8	5.57	35.11	2.86	-1.38	0.05952
Rnu12	546.9	109.17	310.11	31.07	-1.61	0.09616
Robo1	0.2	0.02	0.28	0.02	1.49	0.05017
Romo1	184.8	7.23	148.00	10.15	-1.26	0.03912
Ror1	1.1	0.11	1.50	0.08	1.30	0.07773
RP23-110C17.2	7.6	0.32	10.23	0.48	1.34	0.00612
RP23-111M12.2	0.6	0.04	0.39	0.05	-1.48	0.08709
RP23-218L17.2	0.8	0.16	1.41	0.24	1.85	0.04897
RP23-34P11.2	3.3	0.14	2.63	0.12	-1.25	0.04507
RP23-350E1.2	0.5	0.04	0.34	0.02	-1.47	0.06406
RP23-425N12.2	1.8	0.08	1.16	0.21	-1.57	0.07773
RP23-45G4.3	6.3	0.69	3.80	0.34	-1.74	0.02397
RP23-45P1.1	1.4	0.26	2.53	0.20	1.87	0.00976
RP23-7E4.7	5.4	0.24	6.58	0.29	1.22	0.04484
RP24-247A21.1	0.2	0.01	0.27	0.02	1.49	0.07502
RP24-487F9.4	9.1	0.38	7.52	0.32	-1.21	0.05637
Rpa2	4.8	0.18	3.77	0.19	-1.29	0.02387
Rph3al	2.2	0.10	1.62	0.16	-1.38	0.05844
Rpl13	468.6	7.49	-621.38	3510.62	-1.33	0.00837
Rpl22l1	16.0	1.61	10.85	1.10	-1.56	0.05744
Rpl29	173.1	7.71	135.14	6.66	-1.28	0.01185
Rpl3	40.2	0.77	56.62	2.95	1.40	0.00097
Rpl35	222.8	9.36	185.28	5.86	-1.20	0.03142
Rpl36	383.8	14.44	286.28	7.68	-1.34	0.00129
Rpl37	106.9	3.22	88.39	1.89	-1.21	0.01156
Rpl3l	160.0	2.66	86.81	5.94	-1.86	0.00006
Rpl9	143.1	5.31	115.28	4.56	-1.24	0.01328
Rplp2	219.6	12.53	176.42	15.63	-1.26	0.08721
Rpp21	34.0	1.10	27.40	1.78	-1.26	0.03571
Rps16	168.9	7.80	138.74	8.47	-1.22	0.06774
Rps2	415.9	10.10	329.27	13.54	-1.27	0.00507
Rps21	286.3	9.81	237.66	13.43	-1.21	0.04223
Rps28	560.7	25.56	459.96	13.89	-1.22	0.02203
Rps29	847.9	39.39	674.64	19.42	-1.25	0.01123
Rps6ka3	7.2	0.27	8.78	0.25	1.22	0.01820
Rps6kl1	1.6	0.15	0.97	0.07	-1.67	0.01026
Rrbp1	38.7	1.72	47.48	2.33	1.23	0.03538
Rrp12	4.7	0.36	7.38	0.43	1.59	0.00150
Rsph3a	17.1	0.66	12.48	0.79	-1.38	0.01430
Rsrp1	124.2	7.54	93.33	2.16	-1.32	0.00567
Rtn1	1.6	0.19	0.78	0.09	-1.92	0.01604
Rtn2	60.9	3.15	41.89	2.74	-1.46	0.00336
Rtn4	18.6	0.54	27.41	1.37	1.46	0.00053
Runx1	1.3	0.04	2.46	0.14	1.85	0.00093
Runx2	0.2	0.05	0.37	0.04	2.08	0.02890
Ryr1	0.0	0.01	0.09	0.01	2.06	0.04156

S100a11	52.4	2.24	81.31	4.22	1.55	0.00049
S100a6	55.1	2.58	84.08	7.33	1.51	0.00804
S1pr2	1.5	0.03	2.19	0.21	1.43	0.03300
Samd4	15.7	0.99	19.67	0.95	1.26	0.04105
Saraf	93.2	3.56	70.37	2.75	-1.33	0.00299
Sars2	4.9	0.28	4.05	0.08	-1.20	0.07202
Sash3	0.3	0.02	0.46	0.03	1.46	0.05729
Sbk2	4.2	1.08	2.10	0.67	-2.19	0.07132
Scai	0.8	0.08	1.01	0.04	1.32	0.07439
Scand1	27.5	2.24	8.42	1.67	-3.54	0.00019
Scara3	0.5	0.08	0.77	0.08	1.66	0.05028
Scara5	4.8	0.29	3.37	0.25	-1.46	0.00407
Scd2	2.3	0.27	3.30	0.27	1.43	0.05288
Scml4	0.4	0.04	0.85	0.08	1.88	0.00507
Scn1b	9.7	0.27	14.20	0.71	1.47	0.00092
Scn3a	0.1	0.02	0.18	0.02	1.84	0.03946
Scn4a	5.7	0.40	3.65	0.08	-1.55	0.00077
Scn7a	2.0	0.07	3.08	0.26	1.50	0.00244
Scpep1	4.0	0.29	5.32	0.16	1.30	0.02219
Scrn1	1.4	0.06	1.84	0.10	1.27	0.04890
Scube3	0.2	0.04	0.53	0.05	2.57	0.00770
Sdc1	2.6	0.21	3.57	0.16	1.35	0.04061
Sdcbp2	0.7	0.09	1.32	0.28	1.74	0.07673
Sdf2l1	6.9	0.99	2.37	0.36	-2.63	0.00349
Sdha	474.8	16.11	364.60	4.97	-1.30	0.00146
Sdhb	530.1	22.20	410.76	8.71	-1.29	0.00301
Sdhc	536.6	22.98	432.93	5.66	-1.24	0.00828
Sec16b	0.5	0.06	0.65	0.02	1.37	0.06931
Sec31b	10.0	0.82	7.50	0.41	-1.34	0.02889
Selenbp1	45.7	2.30	33.14	0.59	-1.38	0.00150
Selo	3.1	0.13	2.38	0.11	-1.33	0.01357
Selplg	2.7	0.27	4.55	0.26	1.63	0.00520
Sema3d	0.3	0.02	0.52	0.04	1.65	0.00534
Sema4a	1.4	0.18	2.08	0.21	1.48	0.05183
Sema4c	4.4	0.52	2.73	0.14	-1.55	0.01382
Sema4d	8.5	0.48	6.43	0.47	-1.33	0.02292
Sema5a	0.8	0.07	1.09	0.08	1.39	0.04156
Sema6a	5.5	0.20	3.87	0.18	-1.40	0.00206
Senp7	6.8	0.17	5.45	0.22	-1.23	0.02656
Sept11	6.3	0.23	8.89	0.39	1.42	0.00107
Sept2	20.3	0.82	25.67	1.31	1.26	0.02096
Sept5	2.0	0.17	2.82	0.16	1.35	0.05019
Sergef	2.2	0.16	3.38	0.23	1.46	0.04193
Serpina3n	6.2	0.75	18.68	1.61	3.11	0.00003
Serpinf1	22.4	1.36	41.40	3.88	1.80	0.00110
Serping1	53.7	1.82	91.22	2.56	1.70	0.00002

Serpini1	1.8	0.12	2.82	0.18	1.56	0.00428
Sertad4	1.0	0.07	1.96	0.14	1.97	0.00073
Sesn1	23.6	1.85	19.06	0.43	-1.23	0.05739
Setd7	7.0	0.47	9.00	0.33	1.30	0.01833
Sez6l2	2.2	0.15	3.62	0.28	1.64	0.00383
Sfrp1	2.0	0.07	5.55	0.34	2.70	0.00000
Sfrp2	1.9	0.25	12.02	1.82	6.16	0.00013
Sfrp5	0.4	0.08	0.13	0.05	-4.22	0.00860
Sft2d3	1.5	0.08	1.17	0.04	-1.31	0.04236
Sfxn3	3.6	0.17	4.97	0.08	1.40	0.00147
Sgca	153.1	4.97	118.60	5.33	-1.29	0.00517
Sgk1	37.4	2.43	52.94	1.98	1.42	0.00296
Sgms1	19.4	1.19	15.86	0.89	-1.22	0.08992
Sgol2a	1.4	0.10	0.97	0.06	-1.43	0.03034
Sgpl1	4.9	0.32	6.81	0.38	1.36	0.01431
Sh2b2	0.2	0.02	0.40	0.04	1.65	0.06226
Sh2d3c	11.9	0.58	8.71	0.37	-1.38	0.00310
Sh3bgrl	10.9	0.46	14.81	0.17	1.36	0.00107
Sh3bgrl3	34.9	2.82	45.02	2.49	1.28	0.06161
Sh3bp2	1.2	0.04	1.84	0.14	1.41	0.03287
Sh3bp4	4.1	0.07	5.26	0.11	1.28	0.00195
Sh3d19	6.7	0.19	8.33	0.32	1.24	0.01659
Sh3pxd2b	1.1	0.13	1.59	0.14	1.50	0.06406
Sh3rf1	1.7	0.10	2.22	0.08	1.33	0.01362
Sh3rf3	0.3	0.03	0.73	0.07	2.20	0.00228
Shank1	0.6	0.04	0.74	0.06	1.35	0.08322
Shank3	24.3	0.75	19.10	1.57	-1.29	0.02807
Shc2	0.7	0.06	1.08	0.07	1.58	0.01131
She	2.6	0.11	3.19	0.23	1.22	0.08355
Shmt1	6.6	0.69	8.71	0.44	1.36	0.04127
Siglec1	1.0	0.06	2.11	0.16	2.00	0.00032
Siglece	0.7	0.12	1.12	0.08	1.52	0.08107
Sil1	6.8	0.47	5.36	0.19	-1.26	0.04364
Sipa1l2	9.9	0.57	11.91	0.56	1.20	0.07976
Sirpa	13.4	0.79	17.21	0.51	1.28	0.01365
Six2	2.4	0.22	3.11	0.09	1.34	0.03236
Skil	2.2	0.12	3.66	0.26	1.66	0.00080
Slamf9	2.3	0.31	3.53	0.24	1.47	0.06879
Slc11a1	3.4	0.22	5.53	0.34	1.59	0.00337
Slc12a2	2.0	0.11	2.79	0.11	1.42	0.00228
Slc12a8	0.9	0.11	1.63	0.11	1.80	0.02226
Slc13a4	0.1	0.01	0.25	0.06	4.34	0.00582
Slc15a3	1.4	0.14	2.12	0.16	1.55	0.02809
Slc16a2	2.5	0.17	3.30	0.18	1.37	0.01025
Slc16a3	0.8	0.06	0.99	0.05	1.33	0.08161
Slc16a8	0.5	0.08	0.30	0.04	-1.70	0.06129

Slc17a7	4.5	0.32	3.07	0.27	-1.46	0.01409
Slc1a1	0.7	0.08	0.44	0.08	-1.69	0.05800
Slc1a3	0.8	0.09	2.64	0.21	3.33	0.00016
Slc1a4	0.4	0.03	0.74	0.06	2.03	0.00224
Slc1a7	0.5	0.05	0.30	0.03	-1.58	0.06164
Slc20a1	7.6	0.21	10.54	0.50	1.37	0.00294
Slc22a15	1.1	0.06	1.38	0.10	1.30	0.08700
Slc22a3	1.8	0.13	1.27	0.12	-1.44	0.04428
Slc24a2	0.2	0.03	0.40	0.04	2.59	0.00241
Slc25a1	4.1	0.25	5.21	0.35	1.27	0.08347
Slc25a20	68.8	3.88	49.72	1.41	-1.38	0.00179
Slc25a22	77.5	9.11	49.12	3.97	-1.55	0.01223
Slc25a24	1.0	0.08	1.55	0.08	1.50	0.01150
Slc25a3	841.0	36.53	641.43	12.94	-1.31	0.00227
Slc25a34	59.1	4.05	39.85	2.60	-1.48	0.00310
Slc25a38	9.5	0.16	7.94	0.38	-1.20	0.03985
Slc25a42	20.9	0.94	15.17	1.02	-1.38	0.00633
Slc25a45	2.4	0.08	3.05	0.10	1.26	0.04905
Slc26a10	12.0	0.91	17.61	0.70	1.47	0.00234
Slc27a1	46.6	2.17	28.95	1.81	-1.62	0.00038
Slc28a2	10.0	0.63	5.22	0.22	-1.93	0.00016
Slc29a3	1.1	0.09	1.77	0.11	1.49	0.01283
Slc2a4rg-ps	2.1	0.11	1.64	0.07	-1.29	0.04690
Slc2a8	16.0	0.82	12.62	0.72	-1.28	0.02638
Slc30a4	1.8	0.12	2.11	0.05	1.21	0.07018
Slc36a2	7.6	0.37	4.69	0.47	-1.72	0.00213
Slc38a4	2.6	0.20	3.44	0.38	1.36	0.09596
Slc39a6	2.2	0.14	2.77	0.07	1.28	0.05264
Slc41a2	0.2	0.02	0.35	0.02	1.70	0.00928
Slc43a2	5.7	0.34	8.34	0.36	1.49	0.00197
Slc4a3	113.2	3.97	80.38	3.90	-1.41	0.00101
Slc52a3	0.5	0.06	0.35	0.05	-1.58	0.08786
Slc5a6	7.4	0.53	5.57	0.37	-1.33	0.03675
Slc6a17	0.9	0.02	0.65	0.10	-1.52	0.04564
Slc6a6	16.5	0.77	32.56	2.85	1.95	0.00016
Slc7a1	6.9	0.37	5.26	0.23	-1.30	0.01049
Slc7a2	0.5	0.04	0.74	0.07	1.47	0.03954
SLC7A5	2.2	0.13	3.45	0.28	1.54	0.02562
Slc7a6	1.7	0.11	2.26	0.14	1.32	0.04219
Slc9a3r1	2.5	0.28	1.83	0.16	-1.40	0.07486
Slc9a9	0.9	0.09	1.80	0.07	1.87	0.00090
Slco2a1	0.6	0.05	1.44	0.13	2.41	0.00021
Slco2b1	6.6	0.17	8.26	0.41	1.23	0.02330
Slfn8	0.2	0.03	0.41	0.04	1.63	0.06164
Slit3	2.5	0.24	4.54	0.23	1.80	0.00069
Slitrk4	0.1	0.01	0.12	0.01	1.74	0.04994

Smad1	4.1	0.24	3.09	0.13	-1.29	0.03067
Smad6	3.5	0.24	6.45	0.68	1.80	0.00233
Smad7	3.5	0.33	5.29	0.83	1.49	0.09803
Smad9	0.3	0.02	0.54	0.05	1.62	0.01090
Smco1	7.5	0.37	5.33	0.50	-1.44	0.01257
Smim1	6.7	0.80	10.18	0.61	1.48	0.02744
Smim19	20.5	0.39	16.58	0.78	-1.24	0.01357
Smim20	34.5	1.06	28.01	1.05	-1.23	0.01328
Smim3	3.4	0.25	2.25	0.21	-1.55	0.01050
Smim4	64.6	2.55	52.21	3.88	-1.25	0.08359
Smim5	11.6	0.69	7.66	0.71	-1.49	0.01087
Smo	2.6	0.11	3.17	0.13	1.21	0.06364
Smox	13.0	0.47	10.00	0.41	-1.32	0.00569
Smpd2	15.5	0.59	12.89	0.41	-1.20	0.03538
Smtn	116.8	9.76	83.30	5.40	-1.40	0.01318
Smtnl2	6.0	0.36	4.73	0.33	-1.26	0.07107
Smurf2	5.7	0.09	7.26	0.37	1.25	0.01094
Snai1	1.6	0.12	2.81	0.26	1.81	0.00244
Snai3	2.8	0.30	-6.00	0.84	-2.15	0.00350
Snap91	2.4	0.16	3.03	0.16	1.26	0.07248
Sncg	5.3	0.44	6.79	0.24	1.30	0.05732
Sned1	3.5	0.49	2.18	0.20	-1.61	0.05381
Snhg20	8.2	0.30	6.81	0.55	-1.25	0.09996
Snn	6.3	0.20	4.71	0.34	-1.37	0.00972
Snord104	28728.7	8151.68	14673.79	3133.10	-1.97	0.06533
Snord49a	9265.6	919.83	4202.63	538.66	-2.30	0.00257
Snord49b	4437.1	463.98	1446.64	356.81	-3.52	0.00257
Snord83b	695.9	35.62	374.12	75.16	-1.98	0.05199
Snrk	56.8	3.13	44.69	1.18	-1.27	0.01200
Snrpd2	60.5	1.13	49.45	2.21	-1.22	0.01677
Snx10	2.8	0.13	3.98	0.35	1.40	0.03190
Snx8	5.0	0.37	6.31	0.39	1.25	0.09774
Soat1	1.7	0.10	2.73	0.19	1.62	0.00322
Sod1	237.7	7.44	176.50	12.08	-1.36	0.00612
Sod2	94.5	3.18	77.40	1.58	-1.22	0.00981
Sod3	8.5	0.64	11.93	0.44	1.43	0.00437
Sorbs2	165.4	7.41	215.12	15.00	1.29	0.02480
Sorcs2	2.7	0.28	1.75	0.29	-1.67	0.04077
Sorl1	1.3	0.09	2.13	0.14	1.64	0.00142
Sox13	10.1	0.47	13.26	0.58	1.31	0.00701
Sox5	1.3	0.13	2.18	0.07	1.65	0.00962
Sox9	0.6	0.07	2.02	0.46	3.18	0.00396
Spa17	4.0	0.34	2.91	0.28	-1.44	0.06070
Sparc	189.5	6.53	297.66	20.94	1.56	0.00059
Spata13	6.2	0.55	8.83	0.60	1.42	0.02111
Spata5l1	1.6	0.11	0.97	0.07	-1.72	0.00537

Spats2	1.6	0.06	2.09	0.19	1.30	0.08843
Specc1	0.7	0.05	1.05	0.01	1.61	0.00098
Spef1	3.5	0.14	4.37	0.26	1.27	0.06980
Spg7	34.8	1.96	-42.35	68.20	-1.22	0.01279
Spin1	5.3	0.25	6.55	0.49	1.24	0.07304
Spint2	9.4	0.59	5.44	0.34	-1.69	0.00074
Spire1	0.9	0.03	1.26	0.11	1.42	0.03000
Spn	0.1	0.04	0.29	0.03	3.06	0.01431
Spns2	4.5	0.21	7.99	0.49	1.77	0.00016
Spon1	3.5	0.16	5.09	0.14	1.45	0.00068
Spr	33.6	0.84	24.40	1.21	-1.38	0.00138
Spred1	2.3	0.12	2.88	0.09	1.27	0.01818
Spry4	2.2	0.16	2.85	0.09	1.34	0.01204
Spryd7	11.0	0.37	9.19	0.22	-1.20	0.02377
Spsb4	3.4	0.31	5.38	0.43	1.63	0.00830
Sptan1	72.6	2.53	94.91	2.52	1.31	0.00209
Sqle	0.6	0.03	0.96	0.13	1.49	0.05794
Sqrdl	23.1	1.17	15.31	0.71	-1.53	0.00054
Srebf1	29.2	1.82	20.76	1.32	-1.40	0.00754
Srebf2	5.2	0.26	6.54	0.11	1.27	0.00994
Srgap3	0.1	0.01	0.31	0.02	2.71	0.00023
Srpx	1.8	0.07	3.85	0.29	2.11	0.00030
Srpx2	2.4	0.08	4.74	0.20	2.00	0.00003
Srrm4	6.1	0.60	8.86	0.53	1.48	0.01200
Ssc5d	1.2	0.08	2.15	0.17	1.74	0.00138
Ssfa2	5.6	0.29	7.08	0.18	1.28	0.01249
Sstr4	0.6	0.05	0.98	0.14	1.57	0.09933
Ssu2	3.1	0.13	4.92	0.45	1.51	0.01115
St3gal1	5.7	0.26	7.06	0.15	1.25	0.01238
St8sia6	0.9	0.07	1.34	0.11	1.54	0.01869
Stab1	39.7	3.00	54.41	2.64	1.38	0.01256
Stap2	8.9	0.30	7.08	0.37	-1.29	0.01430
Star	0.7	0.07	0.91	0.05	1.34	0.09659
Stard4	0.9	0.09	1.18	0.03	1.35	0.03442
Stard7	50.7	1.85	41.56	1.93	-1.22	0.02480
Stard9	3.8	0.28	5.05	0.31	1.33	0.03136
Stat1	10.6	0.50	12.91	0.44	1.24	0.03638
Stat2	3.5	0.18	4.32	0.20	1.25	0.02748
Steap4	9.3	0.57	16.36	1.03	1.75	0.00039
Stk32c	0.5	0.08	0.88	0.11	1.83	0.04652
Stmn2	4.1	0.21	6.02	0.50	1.40	0.02718
Stub1	47.2	3.10	37.08	1.28	-1.27	0.02706
Stxbp6	2.0	0.13	1.19	0.10	-1.67	0.00469
Sucla2	154.5	6.23	124.88	5.60	-1.24	0.01793
Suclg1	169.9	6.01	132.81	2.21	-1.28	0.00233
Sulf1	4.0	0.09	5.71	0.52	1.38	0.00933

Sult1a1	23.8	3.02	14.19	0.55	-1.61	0.00611
Susd1	0.4	0.07	0.64	0.05	1.67	0.06826
Susd2	0.9	0.10	1.98	0.15	2.13	0.00132
Svep1	1.2	0.09	6.50	0.71	5.54	0.00000
Swap70	3.8	0.13	4.80	0.11	1.27	0.00574
Swsap1	2.8	0.15	2.24	0.05	-1.24	0.06864
Syde2	3.8	0.32	2.53	0.18	-1.48	0.01029
Syngap1	2.9	0.13	3.57	0.21	1.23	0.08513
Syt7	5.8	0.47	4.21	0.46	-1.39	0.04694
Sytl2	0.2	0.04	0.35	0.05	2.10	0.06750
Syvn1	11.7	0.52	9.46	0.42	-1.23	0.02963
Tab1	6.2	0.24	5.02	0.18	-1.24	0.01822
Tada2a	5.2	0.05	4.17	0.15	-1.24	0.03319
Tagln	10.9	0.41	13.34	0.83	1.23	0.05183
Tagln2	30.1	1.20	40.88	1.54	1.36	0.00173
Tango2	103.5	5.25	80.83	1.89	-1.28	0.00702
Taok3	3.4	0.13	4.74	0.21	1.39	0.00397
Tap1	5.1	0.25	7.64	0.54	1.46	0.00425
Tatdn1	2.2	0.11	2.82	0.09	1.25	0.07534
Tbc1d1	3.1	0.31	4.97	0.50	1.61	0.00804
Tbc1d10c	2.0	0.15	0.97	0.10	-2.19	0.00062
Tbc1d16	14.1	0.63	10.51	0.54	-1.34	0.00556
Tbc1d2b	4.8	0.24	6.00	0.18	1.26	0.01972
Tbc1d30	0.2	0.02	0.33	0.03	1.45	0.07434
Tbc1d4	18.0	0.56	13.49	0.50	-1.33	0.00193
Tbcel	2.1	0.08	3.01	0.28	1.40	0.02343
Tbx15	0.1	0.01	0.27	0.08	5.49	0.01724
Tbx18	0.4	0.03	0.55	0.03	1.45	0.02908
Tbx20	9.2	0.43	11.40	0.30	1.24	0.01455
Tbx3	3.5	0.30	5.44	0.54	1.57	0.01327
Tbxas1	1.0	0.11	1.54	0.16	1.46	0.04838
Tcea3	51.3	4.21	38.98	1.01	-1.30	0.02771
Tcf15	11.5	0.78	6.93	0.84	-1.75	0.00348
Tcf21	4.8	0.36	6.60	0.61	1.36	0.07163
Tcf23	0.3	0.06	0.16	0.01	-1.80	0.04411
Tcirg1	7.9	0.44	9.95	0.68	1.24	0.06170
Tcp11l2	22.5	1.90	17.83	0.56	-1.25	0.05285
Tdrp	1.7	0.12	2.51	0.13	1.43	0.02268
Tesc	58.2	2.55	36.44	0.89	-1.59	0.00009
Tfdp2	15.1	1.18	12.48	0.45	-1.20	0.09498
Tfrc	3.5	0.90	5.99	1.11	1.83	0.08976
Tg	0.9	0.05	0.66	0.08	-1.48	0.04984
Tgfb2	2.7	0.10	5.58	0.88	2.05	0.00299
Tgfb3	3.0	0.23	4.52	0.37	1.50	0.00954
Tgfbi	6.4	0.57	9.97	0.64	1.53	0.00664
Tgif1	2.1	0.26	3.05	0.12	1.47	0.02761

Tgtp1	3.6	0.38	6.23	0.64	1.66	0.06358
Tgtp2	4.5	0.51	7.74	0.88	1.63	0.05927
Thap4	27.4	1.17	22.08	0.58	-1.24	0.01830
Thbd	13.3	0.36	19.61	0.57	1.47	0.00014
Thbs3	2.7	0.13	4.34	0.13	1.63	0.00049
Thbs4	0.9	0.05	2.71	0.59	2.64	0.00804
Them6	4.1	0.39	3.14	0.22	-1.33	0.07920
Thnsl2	2.9	0.14	2.22	0.19	-1.40	0.04255
Thsd7a	1.9	0.18	3.49	0.21	1.87	0.00067
Tiam1	1.7	0.17	2.77	0.21	1.55	0.01731
Tiam2	0.6	0.07	0.90	0.07	1.41	0.05646
Ticam2	0.6	0.04	0.93	0.07	1.67	0.02686
Timp1	2.4	0.21	9.06	1.02	3.94	0.00011
Timp2	22.2	1.16	30.03	1.48	1.35	0.00654
Timp4	27.1	4.17	1.50	0.29	-20.66	0.00001
Tinagl1	23.0	1.30	35.28	2.07	1.53	0.00111
Tinf2	3.9	0.12	2.94	0.18	-1.33	0.01684
Tkt	11.2	0.24	13.92	0.28	1.24	0.00582
Tlcd1	3.3	0.23	2.67	0.14	-1.28	0.07769
Tle3	6.4	0.57	8.92	0.58	1.40	0.02394
Tlr1	1.8	0.25	3.21	0.36	1.74	0.05812
Tlr13	0.2	0.03	0.46	0.02	1.88	0.00635
Tlr2	0.8	0.06	1.32	0.11	1.71	0.00804
Tlr4	1.4	0.14	2.12	0.15	1.53	0.00783
Tlr7	1.4	0.17	2.62	0.22	1.96	0.00592
Tlx1	0.1	0.04	1.56	0.26	20.39	0.00032
Tm4sf1	40.4	2.46	69.45	3.93	1.73	0.00023
Tm6sf1	4.6	0.30	6.91	0.52	1.51	0.00783
Tmc8	2.6	0.16	1.98	0.24	-1.37	0.07594
Tmem106a	3.8	0.17	5.84	0.42	1.51	0.00719
Tmem108	1.1	0.05	1.58	0.18	1.41	0.05838
Tmem119	1.9	0.28	3.03	0.30	1.63	0.04492
Tmem132a	3.3	0.25	4.24	0.15	1.27	0.06563
Tmem134	101.1	2.96	83.92	2.02	-1.21	0.01278
Tmem140	15.9	1.50	11.50	0.70	-1.37	0.04982
Tmem143	67.4	1.77	54.58	1.29	-1.24	0.00500
Tmem144	8.5	0.70	10.94	0.91	1.28	0.08782
Tmem150c	2.6	0.25	1.51	0.18	-1.83	0.00645
Tmem160	23.8	0.79	18.34	1.11	-1.30	0.01327
Tmem164	3.9	0.24	2.86	0.30	-1.37	0.03338
Tmem176a	8.2	0.18	14.07	0.45	1.70	0.00006
Tmem176b	21.2	0.87	31.15	1.39	1.46	0.00083
Tmem2	1.2	0.11	1.55	0.05	1.28	0.06174
Tmem200b	1.7	0.11	2.71	0.32	1.59	0.01621
Tmem229b	1.0	0.04	1.23	0.01	1.24	0.05211
Tmem237	0.5	0.07	0.76	0.07	1.53	0.09508

Tmem238	1.7	0.13	0.87	0.06	-2.00	0.00186
Tmem246	30.6	0.82	23.95	0.99	-1.28	0.00569
Tmem25	3.9	0.19	3.25	0.18	-1.23	0.07329
Tmem256	99.9	3.45	82.45	3.32	-1.20	0.03571
Tmem44	4.2	0.15	5.67	0.28	1.32	0.00768
Tmem45a	0.5	0.04	1.26	0.13	2.50	0.00067
Tmem52	1.2	0.04	0.76	0.07	-1.60	0.03618
Tmem62	1.3	0.06	1.85	0.14	1.44	0.01869
Tmem70	47.4	0.91	38.72	0.55	-1.22	0.00396
Tmem80	11.5	0.42	8.83	0.25	-1.32	0.00727
Tmem82	5.5	0.67	3.15	0.19	-1.68	0.00675
Tmod1	142.4	6.91	115.42	3.93	-1.23	0.02226
Tmod2	0.5	0.02	0.80	0.06	1.76	0.00112
Tmx1	16.9	0.30	12.61	0.50	-1.33	0.00172
Tnc	0.1	0.01	0.95	0.10	8.27	0.00001
Tnfaip3	0.7	0.05	0.94	0.04	1.36	0.04293
Tnfaip8l2	1.2	0.21	2.04	0.14	1.65	0.05187
Tnfrsf11a	0.7	0.06	0.97	0.07	1.38	0.09802
Tnfrsf14	2.2	0.16	3.15	0.15	1.46	0.03699
Tnfrsf1a	31.9	1.24	38.77	0.47	1.22	0.01079
Tnfrsf1b	1.8	0.14	2.90	0.17	1.63	0.00172
Tnfrsf21	3.4	0.09	1.84	0.11	-1.88	0.00008
Tnfrsf23	0.6	0.05	1.09	0.05	1.74	0.00592
Tnfsf10	2.1	0.17	3.54	0.37	1.70	0.00809
Tnni3	11522.0	360.82	8254.28	228.25	-1.40	0.00048
Tns3	5.4	0.20	7.26	0.20	1.35	0.00114
Tnxb	11.2	0.73	15.07	0.83	1.35	0.01309
Top1mt	2.2	0.12	1.74	0.11	-1.30	0.06998
Tox2	1.0	0.10	0.73	0.03	-1.40	0.09155
Tox3	0.9	0.06	1.28	0.05	1.48	0.01663
Tpm2	28.5	3.66	66.87	13.58	2.22	0.00649
Tpm3	23.1	1.05	27.92	0.69	1.21	0.02492
Tpm4	23.3	0.39	34.37	0.54	1.47	0.00004
Tppp	4.1	0.29	2.86	0.15	-1.44	0.00712
Tpt1	630.6	30.20	436.71	25.71	-1.45	0.00190
Tpx2	0.4	0.08	0.98	0.16	2.15	0.03609
Traf1	1.8	0.19	3.15	0.42	1.63	0.07708
Traf3	2.9	0.08	3.88	0.20	1.34	0.00437
Tram2	1.9	0.08	3.11	0.16	1.64	0.00039
Trem2	1.2	0.12	3.24	0.27	2.60	0.00064
Trerf1	0.9	0.04	1.25	0.14	1.37	0.05952
Trf	25.8	1.66	43.06	4.59	1.57	0.03796
Trib1	3.3	0.40	4.28	0.16	1.31	0.07531
Tril	1.3	0.08	2.16	0.19	1.69	0.00429
Trim16	1.8	0.22	2.63	0.07	1.53	0.00846
Trim47	9.7	0.44	13.30	1.00	1.35	0.01646

Trim5	0.8	0.11	1.24	0.09	1.50	0.07634
Trim6	0.6	0.06	1.27	0.09	2.02	0.01327
Trim62	0.3	0.04	0.49	0.05	1.49	0.07553
Trnau1ap	14.0	0.47	11.75	0.57	-1.20	0.06896
Trp53i11	17.8	2.54	6.50	0.92	-2.82	0.00062
Trpc1	2.4	0.18	1.89	0.05	-1.29	0.04967
Trpc3	3.5	0.19	4.78	0.38	1.31	0.04840
Trpm4	4.8	0.18	3.77	0.08	-1.27	0.00601
Trpt1	8.4	0.41	6.59	0.50	-1.24	0.09316
Trpv4	1.8	0.23	2.99	0.28	1.59	0.01618
Tsen15	9.3	0.38	7.72	0.28	-1.23	0.05118
Tsnax	16.1	0.27	12.69	0.48	-1.28	0.00345
Tspan12	18.9	0.84	25.07	1.35	1.33	0.00773
Tspan13	20.7	0.89	16.03	0.45	-1.29	0.00645
Tspan15	2.1	0.09	1.46	0.15	-1.48	0.02549
Tspan17	8.5	0.18	12.82	1.26	1.49	0.02144
Tspan4	19.5	2.83	26.82	0.95	1.41	0.05603
Tspan5	1.3	0.13	1.91	0.18	1.52	0.04632
Tspan7	48.7	2.11	29.23	0.91	-1.67	0.00005
Tspan9	28.1	1.31	39.24	2.48	1.39	0.00556
Ttbk2	1.1	0.07	1.28	0.04	1.22	0.08428
Ttc32	6.9	0.58	5.48	0.33	-1.28	0.09192
Ttc39a	1.0	0.12	1.60	0.15	1.57	0.03196
Ttll1	28.8	1.73	21.50	1.14	-1.34	0.01056
Ttll7	0.9	0.07	1.30	0.14	1.39	0.06421
Tuba1a	50.6	2.79	65.32	3.01	1.28	0.02108
Tubg2	1.9	0.20	2.78	0.17	1.48	0.06792
Tulp3	3.1	0.10	4.05	0.19	1.30	0.01249
Tusc1	1.2	0.12	0.64	0.09	-1.82	0.02133
Tusc3	6.3	0.11	5.34	0.30	-1.21	0.07339
Tvp23a	0.5	0.10	1.55	0.22	2.86	0.00543
Tyro3	0.8	0.04	1.45	0.18	1.79	0.02079
Tyrobp	13.6	0.76	23.97	1.17	1.70	0.00048
Tysnd1	6.7	0.22	5.49	0.11	-1.23	0.01353
Uba52	795.5	89.31	294.39	21.32	-2.54	0.00057
Ubald2	32.8	2.27	25.90	1.15	-1.27	0.03404
Ubash3b	0.8	0.09	1.14	0.03	1.52	0.02656
Ube2b	209.4	9.94	170.88	3.76	-1.22	0.01612
Ubl5	190.6	4.30	150.24	5.91	-1.27	0.00500
Ubtd2	1.3	0.13	2.17	0.27	1.63	0.06577
Uchl1	5.2	0.25	8.08	0.76	1.57	0.01150
Uck2	19.1	0.93	27.86	2.71	1.44	0.01085
Ucp3	10.1	2.16	3.30	0.30	-2.74	0.00207
Ufc1	26.2	0.63	21.52	0.94	-1.22	0.02541
Ugp2	90.4	3.72	125.03	11.97	1.36	0.02876
Uhrf1bp1	1.8	0.14	2.25	0.11	1.25	0.07604

Unc45b	66.2	3.23	50.74	2.03	-1.30	0.00770
Unc93b1	8.3	0.50	12.32	0.64	1.47	0.00193
Unk	6.8	0.36	5.66	0.12	-1.21	0.04293
Upp1	4.1	0.50	6.76	0.53	1.69	0.01535
Uqcc2	166.7	9.51	135.11	2.51	-1.23	0.02248
Uqcr10	889.6	19.36	725.14	10.37	-1.23	0.00349
Uqcr11	1003.8	37.66	766.76	9.19	-1.31	0.00142
Uqcrc1	604.1	22.69	463.70	7.14	-1.30	0.00172
Uqcrfs1	297.3	10.91	225.16	3.65	-1.32	0.00107
Uqcrh	766.1	29.77	593.90	11.70	-1.29	0.00265
Uqcrq	1996.1	72.39	1619.82	31.35	-1.23	0.00761
Urod	81.8	3.05	65.44	1.32	-1.25	0.00588
Usf2	65.4	2.02	53.76	1.26	-1.22	0.00986
Ush1c	1.8	0.12	1.43	0.09	-1.26	0.09192
Ushbp1	15.1	0.37	12.11	0.43	-1.25	0.00674
Usp17la	0.9	0.05	1.25	0.05	1.39	0.02931
Usp43	1.0	0.07	1.54	0.14	1.55	0.01327
Vasp	18.0	0.88	22.41	1.93	1.23	0.09194
Vat1	8.7	0.40	11.64	0.68	1.32	0.01328
Vav1	0.6	0.04	1.11	0.10	1.65	0.01208
Vcam1	4.1	0.40	6.39	0.29	1.58	0.00396
Vcan	3.5	0.37	6.84	1.10	1.95	0.00337
Vegfb	190.7	4.45	146.04	4.67	-1.31	0.00150
Vgll3	0.6	0.11	1.89	0.23	3.15	0.00065
Vim	125.8	8.11	201.40	10.15	1.61	0.00076
Vti1b	46.6	1.54	37.14	1.22	-1.26	0.00693
Vwa1	3.0	0.06	5.12	0.31	1.69	0.00018
Vwa3a	5.2	0.78	3.07	0.45	-1.72	0.04176
Vwa8	23.0	0.99	17.06	0.64	-1.35	0.00224
Vwf	17.1	0.65	22.23	2.20	1.28	0.08039
Wbp1	44.2	1.68	36.35	0.84	-1.22	0.01445
Wbp5	5.6	0.22	7.54	0.44	1.36	0.01572
Wbscr27	0.7	0.08	1.26	0.21	1.80	0.06629
Wdfy4	1.0	0.13	1.53	0.17	1.49	0.06639
Wdr45	26.8	1.12	20.25	0.75	-1.33	0.00350
Wdr66	0.4	0.02	0.58	0.02	1.34	0.06866
Wdr91	3.4	0.16	4.31	0.20	1.24	0.05351
Wdsub1	10.9	0.40	8.96	0.22	-1.23	0.02706
Wfdc17	4.9	0.49	11.11	0.39	2.22	0.00027
Whrn	11.4	0.80	8.20	0.53	-1.38	0.01375
Wif1	0.5	0.04	0.39	0.04	-1.43	0.09241
Wipf1	4.6	0.29	5.56	0.24	1.21	0.08691
Wisp2	4.2	0.46	27.70	2.75	6.67	0.00000
Wnk1	201.1	16.58	250.82	17.02	1.25	0.08640
Wnk2	21.2	1.06	16.31	1.54	-1.32	0.05047
Wnt11	3.2	0.13	1.95	0.26	-1.75	0.00396

Wnt9b	0.1	0.01	0.17	0.02	1.65	0.08591
Wscd1	2.3	0.16	1.55	0.09	-1.54	0.00510
Xbp1	24.3	0.79	18.18	0.48	-1.33	0.00138
Xdh	15.5	1.17	10.91	0.43	-1.41	0.00742
Xirp2	43.7	4.88	72.49	8.73	1.65	0.01295
Xpnpep2	0.6	0.07	0.31	0.02	-1.71	0.05785
Xpr1	4.7	0.20	5.95	0.14	1.28	0.00582
Xylt2	4.2	0.18	5.30	0.35	1.25	0.04590
Yars2	11.8	0.79	9.59	0.51	-1.23	0.07967
Ybx1	274.5	11.86	190.88	7.23	-1.44	0.00069
Ybx2	33.5	3.01	21.27	1.05	-1.57	0.00271
Ybx3	69.0	2.54	56.35	1.95	-1.23	0.01604
Yes1	3.8	0.26	3.00	0.16	-1.23	0.09901
Yipf4	7.0	0.42	5.71	0.14	-1.22	0.04048
Yjefn3	12.2	1.16	8.04	0.63	-1.58	0.01416
Ypel3	75.8	4.82	59.44	4.62	-1.28	0.05019
Ywhaq	13.6	0.49	17.08	0.35	1.26	0.00574
Zbp1	0.5	0.07	1.13	0.11	2.27	0.00440
Zbtb42	5.9	0.31	4.87	0.15	-1.21	0.08968
Zbtb7c	0.5	0.01	0.62	0.04	1.36	0.04761
Zdhhc1	8.6	0.49	7.16	0.28	-1.21	0.06278
Zdhhc20	1.8	0.06	2.16	0.13	1.21	0.09372
Zeb1	12.2	0.48	15.44	0.45	1.26	0.00783
Zeb2	12.2	0.77	16.16	0.50	1.33	0.00710
Zfand4	1.2	0.06	0.75	0.15	-1.77	0.02958
Zfhx3	1.2	0.10	1.52	0.06	1.27	0.05833
Zfp157	1.4	0.06	1.11	0.04	-1.25	0.05770
Zfp189	1.1	0.05	0.88	0.04	-1.29	0.06851
Zfp213	0.9	0.08	0.62	0.04	-1.44	0.04838
Zfp3	2.4	0.09	1.78	0.08	-1.33	0.02539
Zfp36	50.2	7.08	28.43	2.68	-1.73	0.00841
Zfp365	0.1	0.02	0.42	0.09	3.09	0.00601
Zfp423	1.0	0.08	1.62	0.10	1.70	0.00397
Zfp442	0.6	0.06	0.95	0.08	1.68	0.01675
Zfp462	0.9	0.06	1.61	0.10	1.79	0.00044
Zfp568	2.2	0.06	2.97	0.15	1.33	0.00493
Zfp651	20.1	0.73	25.16	2.00	1.24	0.05393
Zfp668	2.0	0.07	1.63	0.10	-1.24	0.07368
Zfp704	2.5	0.15	3.13	0.15	1.26	0.02898
Zfp771	10.8	0.57	7.34	0.66	-1.49	0.00697
Zfpm2	3.8	0.20	2.96	0.17	-1.28	0.02692
Zfr2	1.5	0.07	1.15	0.09	-1.30	0.09035
Zfyve21	18.8	0.72	13.04	0.72	-1.47	0.00121
Zhx1	4.7	0.16	5.89	0.14	1.24	0.01042
Zscan18	0.3	0.02	0.38	0.04	1.52	0.09888
Zyx	22.9	0.77	29.84	1.99	1.29	0.02108

List 4: All genes which exhibit fold change >1.2 or <-1.2 (and FDR < 0.1) in SUR2$^{wt/AV}$ hearts, from RNASeq analysis.

Gene	WT Expression (FPKM)		wt/AV Expression (FPKM)		Fold Change	Adj. P Val (FDR)
	Mean	SEM	Mean	SEM		
1110008F13Rik	133.78	9.47	82.33	8.47	-1.60	0.08601
2610507B11Rik	3223.77	201.81	4175.17	177.68	1.31	0.08312
A530016L24Rik	620.77	48.31	381.05	35.28	-1.63	0.08015
Abhd17a	283.93	14.03	185.11	13.01	-1.52	0.02338
Acbd6	330.87	15.83	224.17	13.44	-1.46	0.01763
Acot1	64.16	4.22	37.35	4.84	-1.75	0.04976
Acot2	542.99	25.89	382.28	8.52	-1.41	0.01745
Acta1	1415.97	199.76	7824.03	2322.23	4.57	0.04651
Adra1b	434.45	53.06	262.50	22.12	-1.61	0.07253
Adssl1	1389.41	53.82	1107.52	49.90	-1.25	0.08700
Akt1s1	551.42	22.77	431.87	15.91	-1.27	0.06994
Aldh2	2570.39	68.61	2100.63	102.16	-1.22	0.09807
Aqp1	3174.83	237.89	1995.11	250.16	-1.61	0.06705
Atp2c1	328.12	17.17	438.12	9.95	1.34	0.04318
Bag6	2436.38	77.74	1902.60	91.24	-1.28	0.08634
Bcam	2709.82	92.05	2184.96	62.22	-1.23	0.08206
Bdh1	1135.94	144.67	1741.57	104.67	1.59	0.08869
Cald1	619.11	7.35	759.60	22.93	1.23	0.07991
Camk2a	711.83	16.92	572.27	15.64	-1.24	0.05918
Ccdc85b	72.79	6.45	37.26	5.36	-1.93	0.09750
Cdc37l1	495.72	23.51	649.30	30.63	1.31	0.06564
Cebpb	282.92	31.56	127.78	13.86	-2.17	0.03464
Cfh	729.97	46.97	1010.49	51.59	1.39	0.06705
Clec2d	165.37	11.81	256.60	14.39	1.56	0.04651
Clip1	2723.52	135.20	3443.63	62.52	1.27	0.06431
Cnot3	325.54	22.68	221.95	16.62	-1.45	0.08519
Cox5a	4502.15	436.62	2383.65	183.70	-1.86	0.03452
Crat	6932.84	214.98	5720.04	155.56	-1.21	0.08519
Cxcl12	2304.86	94.77	3343.94	171.87	1.45	0.04976
Dmd	1127.63	20.25	1472.70	91.26	1.30	0.05383
Egln3	1061.51	81.62	548.47	36.79	-1.92	0.03784
Eif3f	1373.18	23.43	1014.11	28.43	-1.35	0.04894
Emcn	362.58	15.01	481.37	11.12	1.34	0.04976
Endog	213.41	13.94	122.03	14.20	-1.73	0.04894
Fscn1	447.68	18.67	300.49	21.10	-1.50	0.06994

Fth1	16311.28	840.46	12519.80	632.48	-1.30	0.06824
Gata6	672.19	21.93	492.15	8.26	-1.36	0.01615
Gm12940	19.77	2.94	32.50	1.66	1.73	0.08601
Gm17131	111.80	5.76	161.06	11.40	1.45	0.08354
Gm26917	17360.45	2525.31	9784.25	940.41	-1.73	0.08206
Gm5532	440.85	30.94	616.71	47.63	1.41	0.07835
Gnas	685.20	83.72	254.70	31.26	-2.65	0.01745
Heg1	1334.37	80.30	1858.30	179.33	1.38	0.09978
Hmg20b	472.35	25.33	355.95	21.03	-1.32	0.07882
Hnrnpa0	573.92	20.95	373.01	26.55	-1.54	0.01763
Htra1	881.33	14.61	718.12	30.94	-1.23	0.09540
Htra3	1172.87	44.98	925.51	26.38	-1.26	0.09978
Ints1	840.34	57.38	618.72	25.56	-1.34	0.09750
Irf2bpl	662.62	42.20	462.15	27.11	-1.42	0.08298
Isyna1	308.01	44.35	184.88	12.40	-1.57	0.08482
Jund	796.32	84.47	538.47	42.00	-1.45	0.08601
Kcng2	1284.65	40.79	975.75	32.71	-1.31	0.03784
Kcnh2	1024.85	52.95	777.56	37.17	-1.31	0.08412
Kif5b	1292.90	73.55	1740.98	129.00	1.34	0.09540
Klf2	1342.45	132.59	699.84	38.54	-1.88	0.00491
Klhl24	1353.84	75.41	1804.53	59.46	1.34	0.03452
Lipe	493.40	28.89	364.30	19.06	-1.35	0.08880
Maf1	685.67	12.01	550.42	27.54	-1.25	0.09978
Map1lc3a	5119.62	338.60	3821.87	163.81	-1.33	0.07980
Map2k2	921.17	55.30	678.87	34.07	-1.35	0.08015
Mgat4b	809.20	19.70	565.61	25.50	-1.43	0.03452
Mrps34	191.83	24.70	92.47	14.42	-2.04	0.05720
Myot	379.20	30.13	633.42	28.46	1.68	0.02464
Nppb	659.11	135.84	1770.65	223.38	2.85	0.03452
Orc4	154.41	7.79	217.49	16.07	1.39	0.09515
P3h4	278.94	13.66	180.42	7.91	-1.53	0.04976
Pam	4382.25	151.83	5611.23	372.77	1.28	0.08482
Pdlim5	4247.89	89.42	5459.15	322.59	1.28	0.06994
Pex6	475.48	17.58	358.43	12.22	-1.32	0.04318
Pfkl	1223.90	46.46	980.58	29.41	-1.24	0.08206
Pfn1	1716.84	67.05	1364.51	44.91	-1.25	0.08015
Pgp	209.16	4.84	139.88	10.20	-1.49	0.03757
Phospho1	44.99	5.17	21.57	1.58	-1.95	0.05216
Picalm	1137.00	58.15	1634.56	125.19	1.43	0.06446
Ppp1r3c	1078.33	86.35	1486.93	40.82	1.40	0.04894
Ppp2r3d	219.10	16.60	136.08	10.55	-1.59	0.03452
Ppp2r5d	516.40	19.67	408.92	10.55	-1.25	0.08634
Ptp4a3	3667.92	76.18	2760.35	62.21	-1.32	0.03452
Rgma	617.76	19.13	382.56	30.40	-1.62	0.04894
Rnf187	1475.76	56.17	1212.54	25.24	-1.21	0.09978

RP24-390N7.1	115.72	9.47	177.96	12.91	1.52	0.07835
Rpl27a	1052.09	47.49	808.03	58.22	-1.30	0.08601
Rps2	2758.96	132.42	2151.86	70.62	-1.27	0.05620
Rragd	926.37	23.41	1133.84	32.84	1.23	0.06994
Scand1	152.49	21.18	57.64	10.64	-2.57	0.08015
Sh2d3c	413.13	19.51	303.56	22.07	-1.37	0.08519
Skiv2l	346.89	5.98	275.49	9.02	-1.25	0.08601
Slc25a42	1091.43	64.54	785.05	53.69	-1.39	0.06994
Slc27a1	1758.05	73.16	1189.71	60.37	-1.47	0.01286
Slc4a3	3500.08	196.77	2729.60	138.30	-1.28	0.08519
Smpd2	268.59	6.81	213.78	4.72	-1.25	0.06994
Stk11	1063.20	35.45	876.21	14.84	-1.21	0.09978
Stub1	333.31	13.73	250.70	15.20	-1.32	0.06446
Svil	2848.68	114.75	3619.35	218.57	1.27	0.09360
Tead1	2101.73	70.07	2645.95	149.45	1.26	0.09851
Tesc	767.40	63.94	498.13	21.17	-1.51	0.03452
Tnfrsf12a	191.56	18.33	284.56	17.41	1.51	0.08519
Tob2	1251.58	19.75	834.72	43.89	-1.50	0.01745
Trp53i11	505.16	53.07	275.50	23.01	-1.81	0.06994
Ugp2	1920.33	103.60	2708.87	190.99	1.41	0.04922
Usf2	965.80	32.95	766.99	24.07	-1.25	0.05531
Xirp2	5952.35	241.71	8950.16	624.26	1.50	0.01745
Ybx1	4816.43	103.44	3773.68	65.02	-1.27	0.02633
Ybx2	351.17	50.47	203.89	28.65	-1.69	0.08601
Zbtb17	271.31	11.26	214.86	8.53	-1.25	0.09978
Zfp771	130.83	6.96	73.72	6.84	-1.75	0.06431

List 5: All genes which exhibit fold change >1.2 or <-1.2 (and FDR < 0.1) in SUR2$^{AV/AV}$ hearts, from RNASeq analysis.

Gene	WT Expression (FPKM)		wt/AV Expression (FPKM)		Fold Change	Adj. P Val (FDR)
	Mean	SEM	Mean	SEM		
2310003H01Rik	233.76	17.97	180.19	10.70	-1.29	0.09494
2310040G07Rik	48.26	2.67	29.36	1.48	-1.63	0.02892
2310067B10Rik	2050.59	173.00	1497.44	69.09	-1.35	0.03084
4931406P16Rik	260.05	38.44	395.08	17.99	1.59	0.08111
8430408G22Rik	779.02	61.53	1853.82	195.08	2.37	0.00755
9030617O03Rik	629.07	18.78	481.24	24.04	-1.30	0.02021
9330158H04Rik	135.76	6.80	97.65	11.75	-1.38	0.07441
A530016L24Rik	620.77	48.31	391.93	42.10	-1.62	0.03084
Abhd17c	286.52	6.19	227.68	4.49	-1.26	0.02732
Ablim2	321.67	33.03	214.60	7.29	-1.46	0.02883
Ablim3	826.70	27.14	596.10	34.44	-1.39	0.01124
Abra	409.53	38.20	638.86	26.70	1.59	0.01652

Acaa2	6815.92	144.18	5392.24	501.29	-1.28	0.06777
Acbd6	330.87	15.83	264.51	6.39	-1.23	0.08170
Ace	728.49	62.38	1065.87	105.89	1.47	0.07947
Acot1	64.16	4.22	25.16	1.47	-2.59	0.00032
Acot2	542.99	25.89	347.18	10.63	-1.56	0.00022
Acp6	292.01	7.49	243.09	12.83	-1.22	0.08527
Acsf2	572.33	49.19	401.22	34.99	-1.43	0.04425
Acta1	1415.97	199.76	8391.24	2351.83	5.23	0.00523
Actc1	109622.68	6830.93	88519.81	2409.00	-1.23	0.07223
Actn4	1319.00	49.25	1823.44	107.01	1.38	0.00727
Acvrl1	412.63	12.19	526.66	41.18	1.27	0.09845
Adprhl1	3461.73	186.92	4443.49	301.02	1.28	0.05167
Adra1b	434.45	53.06	228.56	11.10	-1.84	0.00514
Agpat3	2425.01	107.66	1802.87	55.86	-1.34	0.00245
AI480526	139.71	14.51	87.89	12.49	-1.63	0.09779
Alad	375.61	16.49	296.70	3.83	-1.25	0.02247
Ankrd1	5620.79	890.92	12511.67	1293.15	2.32	0.00657
Ankrd23	133.48	8.14	240.11	35.19	1.73	0.01695
Anxa1	131.57	12.56	208.77	15.66	1.60	0.01506
Anxa2	509.67	33.07	802.28	51.18	1.58	0.00145
Anxa3	150.22	19.03	290.89	11.50	1.97	0.00091
Apbb2	570.72	30.11	447.30	8.59	-1.27	0.02257
Aplnr	494.95	30.48	200.35	24.29	-2.59	0.00086
Apoe	2001.42	66.83	2490.55	119.10	1.24	0.03154
Aqp1	3174.83	237.89	609.22	63.72	-5.33	0.00000
Arhgdib	202.55	4.90	282.78	22.24	1.38	0.01773
Asah1	237.05	9.04	308.28	19.54	1.28	0.08877
Atp6v1e1	711.96	14.65	876.96	39.04	1.23	0.04337
B2m	2639.36	156.62	3699.94	345.23	1.39	0.03613
B4galt3	141.31	8.77	101.23	6.39	-1.37	0.03544
BC029214	128.44	5.39	164.77	9.27	1.28	0.09776
Bcl2l1	507.49	40.43	781.31	92.49	1.53	0.05212
Bdh1	1135.94	144.67	2211.71	215.44	1.99	0.00243
Bst2	117.27	12.40	178.35	12.07	1.53	0.01894
C130080G10Rik	428.53	28.00	330.72	18.05	-1.30	0.07563
C1qa	346.52	19.72	440.86	17.94	1.29	0.04909
C1qb	385.06	21.22	506.35	25.90	1.32	0.02454
C1qc	345.27	7.48	424.64	18.96	1.24	0.05118
C1qtnf9	365.14	23.89	469.74	22.97	1.30	0.05895
C1s1	138.72	7.10	43.73	21.22	-6.81	0.00902
Cacfd1	378.27	13.47	297.80	17.29	-1.28	0.03452
Cald1	619.11	7.35	841.94	30.91	1.36	0.00166
Camk2a	711.83	16.92	512.79	14.23	-1.38	0.00086
Cand2	1639.11	66.74	1239.83	96.75	-1.33	0.04259
Car4	196.25	9.61	114.09	11.32	-1.80	0.00054

Cbr2	185.28	9.03	251.00	11.41	1.36	0.01455
Ccl6	142.00	6.86	223.41	25.04	1.54	0.03106
Cd59a	866.75	40.89	546.99	12.43	-1.57	0.00230
Cdc37l1	495.72	23.51	629.62	18.68	1.27	0.04039
Cdk18	352.09	17.76	243.05	11.92	-1.44	0.00786
Cdv3	924.69	30.44	1244.71	39.24	1.35	0.01319
Ces1d	1134.28	58.43	932.33	40.90	-1.21	0.09721
Cfh	729.97	46.97	1152.50	70.02	1.58	0.00210
Cic	1161.48	61.67	907.09	72.60	-1.29	0.09735
Cited2	493.36	17.49	353.44	38.11	-1.40	0.06028
Cldn5	195.82	37.72	69.45	6.73	-2.59	0.01481
Clec2d	165.37	11.81	226.20	21.44	1.36	0.07500
Clic5	2034.59	134.26	2746.28	114.29	1.36	0.03195
Clip1	2723.52	135.20	3477.73	119.96	1.28	0.01526
Cluh	4526.75	168.35	3702.44	201.46	-1.22	0.09704
Cnot3	325.54	22.68	237.60	18.60	-1.36	0.07717
Col3a1	1978.77	18.60	2658.79	79.63	1.34	0.00523
Coro2b	254.99	13.40	194.39	15.81	-1.32	0.06151
Cox19	290.15	9.83	373.36	19.55	1.29	0.04225
Cpt2	2453.76	73.69	1936.90	25.84	-1.26	0.00800
Crat	6932.84	214.98	5509.05	135.81	-1.25	0.01057
Cript	193.60	5.67	258.38	16.38	1.31	0.04880
Cs	15294.08	408.43	12751.70	540.16	-1.20	0.07644
Csf1	789.80	37.70	632.37	27.01	-1.25	0.05056
Ctgf	442.75	54.60	947.30	39.12	2.20	0.00065
Cxcl12	2304.86	94.77	5499.46	491.46	2.36	0.00001
Dcn	4756.00	193.69	5830.77	378.56	1.22	0.08744
Dennd1a	424.62	30.54	325.10	14.27	-1.29	0.06393
Dennd4b	964.37	42.20	756.96	52.19	-1.28	0.06151
Dhrs11	792.80	26.76	612.78	22.13	-1.28	0.02257
Dnajc30	235.23	10.43	188.07	6.49	-1.23	0.09905
Dpt	517.95	11.67	627.63	19.18	1.21	0.05825
Dpysl3	343.58	11.83	416.61	13.44	1.22	0.06379
Dsg2	851.94	87.75	610.45	55.60	-1.39	0.08578
Ech1	11422.05	220.77	8524.14	702.62	-1.35	0.01535
Eci1	1928.79	69.45	1476.96	38.07	-1.30	0.01056
Efnb3	1135.67	171.71	703.93	59.72	-1.55	0.04772
Egln1	4357.14	84.65	3541.56	156.93	-1.23	0.04424
Egln3	1061.51	81.62	392.22	73.89	-2.83	0.00021
Eif3f	1373.18	23.43	1119.19	82.71	-1.23	0.07763
Eif4ebp2	541.25	33.39	414.43	35.59	-1.31	0.08151
Elovl1	191.28	3.61	242.50	4.42	1.27	0.02257
Emcn	362.58	15.01	569.03	32.18	1.56	0.00048
Emp1	474.74	27.11	732.86	38.91	1.55	0.02446
Emp2	601.60	14.76	484.07	17.84	-1.25	0.02133

Enah	1105.80	24.48	1500.27	67.92	1.36	0.02257
Eng	2846.40	102.97	4258.53	390.89	1.48	0.00899
Entpd5	1730.49	23.44	1396.69	72.49	-1.24	0.04483
Ephx1	377.70	43.98	539.59	37.15	1.45	0.05889
Ephx2	2182.43	89.25	1619.08	71.40	-1.35	0.01237
Erp29	260.17	6.32	312.54	10.51	1.20	0.08295
Ets2	1123.39	51.35	836.24	64.68	-1.36	0.03636
Fah	272.08	30.14	177.08	21.41	-1.55	0.08295
Fam174b	2324.80	69.37	1862.78	56.81	-1.25	0.01575
Fam213b	396.15	37.77	565.71	37.13	1.44	0.05261
Fbln2	1180.49	34.82	1661.42	161.91	1.39	0.01886
Fbp2	230.31	21.57	120.37	3.45	-1.90	0.00061
Fbxo21	469.69	16.83	354.21	16.98	-1.33	0.00682
Fgf1	2202.94	98.58	1783.46	48.14	-1.23	0.05301
Fgf13	654.98	12.55	514.17	28.44	-1.28	0.03800
Fhod3	3116.58	201.33	2432.51	109.86	-1.27	0.04880
Flna	1795.91	126.32	2438.45	218.87	1.36	0.08179
Fmnl1	314.59	9.84	235.16	18.96	-1.33	0.06248
Fn1	725.63	67.29	1262.01	102.93	1.75	0.00499
Fscn1	447.68	18.67	269.92	29.35	-1.72	0.00320
Fstl1	802.99	41.23	1181.24	31.07	1.48	0.00099
Fth1	16311.28	840.46	13236.58	387.03	-1.23	0.07500
Fxyd5	183.13	11.28	262.31	20.74	1.44	0.01577
Gaa	1905.56	107.58	1477.91	59.63	-1.28	0.04259
Gas5	129.89	16.84	186.71	4.62	1.45	0.05212
Gata6	672.19	21.93	533.62	14.96	-1.25	0.01385
Gm10222	45.07	4.96	25.53	3.95	-1.79	0.09498
Gm10925	144.48	12.30	85.72	11.98	-1.78	0.03152
Gm12840	98.44	12.75	267.80	32.17	2.74	0.00143
Gm15440	226.89	3.96	166.71	10.83	-1.36	0.01455
Gm24265	24.83	4.25	11.18	1.47	-2.09	0.03012
Gm26809	2032.29	215.52	4609.29	641.55	2.23	0.00243
Gm26917	17360.45	2525.31	11049.29	1171.53	-1.54	0.08927
Gm29408	8.13	1.94	41.80	11.32	5.62	0.03389
Gm3839	18.72	2.86	6.30	1.02	-2.69	0.02003
Gm5532	440.85	30.94	621.99	31.86	1.43	0.02050
Gnas	685.20	83.72	376.75	60.85	-1.79	0.05167
Gngt2	68.69	2.67	122.10	11.35	1.69	0.00575
Gpt	294.02	16.62	205.37	7.42	-1.40	0.01131
Gramd1b	450.23	19.01	310.07	26.09	-1.48	0.01190
Grb14	1646.83	49.79	1308.43	47.30	-1.26	0.01455
H2-D1	1069.16	65.29	1417.08	73.91	1.33	0.04305
H2-K1	926.91	30.77	1525.43	102.82	1.64	0.00068
H2-Q4	156.59	4.85	229.60	25.53	1.44	0.05416
H2-T23	178.13	10.66	236.58	10.16	1.34	0.09776

Hacd1	267.64	12.46	356.33	21.12	1.34	0.04403
Hadh	5321.59	171.06	4417.63	101.08	-1.20	0.04425
Heg1	1334.37	80.30	1923.44	115.05	1.44	0.02052
Hfe2	2188.13	21.29	1692.79	65.54	-1.29	0.00619
Hhatl	1083.84	46.49	877.40	45.05	-1.23	0.07563
Hnrnpa0	573.92	20.95	401.96	22.49	-1.42	0.01238
Hnrnpl	1755.86	85.83	1401.73	19.44	-1.24	0.05415
Hrc	12762.05	268.56	10458.02	288.04	-1.22	0.02403
Htra1	881.33	14.61	709.16	24.92	-1.24	0.02884
Ifi27	158.34	9.99	213.13	9.82	1.34	0.08824
Ifitm2	441.74	23.66	527.98	12.23	1.20	0.07764
Ifngr1	433.66	15.66	610.00	20.93	1.41	0.00201
Igfbp7	1485.29	36.16	1997.82	101.50	1.34	0.00621
Ints1	840.34	57.38	652.33	42.31	-1.28	0.08595
Iqsec1	1825.64	107.48	1325.81	106.78	-1.39	0.03452
Irf2bpl	662.62	42.20	458.57	38.92	-1.44	0.02429
Irx4	453.46	33.47	343.72	13.85	-1.30	0.05245
Itga7	2046.52	120.96	1628.64	19.37	-1.25	0.03368
Ivns1abp	7747.58	686.11	10704.36	179.12	1.40	0.01575
Jund	796.32	84.47	574.15	33.93	-1.35	0.08981
Kcnh2	1024.85	52.95	771.66	40.08	-1.32	0.02676
Kcnj3	499.82	29.25	342.36	30.97	-1.48	0.04006
Kif5b	1292.90	73.55	1656.61	72.95	1.29	0.08138
Klf2	1342.45	132.59	889.25	46.93	-1.48	0.01506
Lama5	3603.51	468.64	2351.81	170.64	-1.50	0.05415
Lap3	354.91	28.33	467.21	21.37	1.34	0.05908
Lgals4	622.10	62.79	271.47	10.67	-2.26	0.00682
Lipe	493.40	28.89	343.92	21.90	-1.43	0.01319
Lmod2	5874.16	425.79	7353.83	238.07	1.27	0.07752
Lpcat3	369.80	18.74	290.07	21.44	-1.30	0.05380
Lrrc24	674.87	36.11	538.92	16.27	-1.24	0.05610
Lrrc3b	290.22	15.62	201.41	12.66	-1.44	0.03363
Lum	402.34	39.50	624.13	42.56	1.56	0.01353
Ly6a	772.55	23.13	1149.90	49.54	1.49	0.00085
Ly6c1	897.98	32.87	1483.79	56.10	1.66	0.00021
Lyz2	745.03	37.65	1005.09	129.29	1.33	0.09623
Maob	505.71	12.32	401.76	37.66	-1.27	0.08484
Map1lc3a	5119.62	338.60	3932.57	185.88	-1.29	0.04483
Map2k2	921.17	55.30	727.19	42.88	-1.26	0.09387
Map7d1	3895.20	149.41	3113.70	208.02	-1.26	0.08711
Mbd6	571.59	37.95	392.49	41.11	-1.48	0.05167
Mcf2l	748.76	48.09	1179.67	58.88	1.58	0.00131
Mcfd2	402.25	27.35	504.15	22.12	1.26	0.09721
Med25	898.66	58.91	699.10	44.92	-1.28	0.08484
Mef2d	2661.76	168.34	2061.37	153.26	-1.29	0.07980

Meis2	306.15	12.11	226.65	4.62	-1.34	0.00450
Mfap5	169.44	8.20	246.04	9.37	1.44	0.02003
Mgat4b	809.20	19.70	586.90	40.88	-1.38	0.01190
Mgp	1257.71	60.34	1550.45	35.82	1.24	0.07952
Mid1ip1	936.26	59.45	720.05	39.64	-1.29	0.07972
Mllt4	777.02	17.33	611.36	28.90	-1.28	0.01779
Mllt6	1677.91	109.36	1273.45	106.28	-1.33	0.09930
Mlycd	898.49	30.10	719.18	40.59	-1.25	0.05986
Mmd	433.87	16.37	330.15	20.21	-1.33	0.02127
Mrpl3	765.11	31.02	950.97	47.07	1.24	0.06151
Mt2	115.13	20.92	242.05	49.61	2.07	0.06859
mt-Nd6	111678.51	4601.64	84987.67	4908.21	-1.32	0.08796
Myadm	2848.99	53.34	2263.30	168.18	-1.27	0.06235
Mybpc3	32049.67	1940.28	39612.64	1392.66	1.24	0.07576
Myl12a	5550.02	380.12	6782.29	251.54	1.23	0.09721
Myl12b	436.16	9.38	533.60	12.23	1.22	0.01931
Myl9	388.21	44.33	621.69	23.27	1.65	0.00737
Myot	379.20	30.13	804.60	79.22	2.09	0.00021
Nav2	1518.18	108.40	1125.42	83.41	-1.35	0.04305
Ncapd2	279.33	12.71	213.66	5.93	-1.30	0.03084
Neurl2	168.67	8.41	129.61	6.32	-1.26	0.07764
Nfix	3123.52	116.19	2333.82	247.34	-1.37	0.07597
Nosip	244.38	13.30	305.25	13.85	1.24	0.07548
Nppa	442.86	60.95	1350.71	319.50	2.81	0.03748
Nppb	659.11	135.84	2264.56	451.88	3.42	0.00185
Oplah	627.68	15.08	514.21	16.68	-1.21	0.03957
P3h4	278.94	13.66	157.97	18.62	-1.81	0.00111
Palm	461.87	16.80	358.00	26.54	-1.31	0.05291
Pam	4382.25	151.83	5491.13	136.53	1.26	0.04483
Pde4a	1191.47	66.04	822.00	32.69	-1.44	0.00184
Pdgfa	190.14	6.96	150.46	6.68	-1.25	0.05959
Pdgfb	518.36	34.88	745.59	46.60	1.45	0.01543
Pdlim5	4247.89	89.42	5443.01	172.65	1.28	0.02153
Pecam1	1725.11	45.49	2290.25	61.33	1.33	0.00212
Pfdn1	372.71	11.59	468.65	19.17	1.25	0.07764
Pgp	209.16	4.84	148.83	11.10	-1.37	0.02711
Picalm	1137.00	58.15	1757.57	118.56	1.54	0.00512
Pitpnc1	1028.03	55.80	805.83	37.05	-1.27	0.04159
Plbd1	436.46	13.92	255.02	9.90	-1.73	0.00021
Plxnb1	1022.37	80.23	726.53	37.85	-1.39	0.01313
Plxnd1	2377.95	136.79	1851.68	90.46	-1.28	0.07652
Pnrc1	972.03	77.76	739.92	49.32	-1.30	0.06472
Postn	300.00	30.59	507.81	21.75	1.70	0.00284
Ppap2a	390.68	20.63	594.81	14.82	1.54	0.00048
Ppp1r13l	560.27	41.34	366.64	33.99	-1.53	0.02181

Ppp1r3c	1078.33	86.35	1579.61	77.61	1.48	0.00374
Prkab1	465.97	39.05	330.13	21.57	-1.40	0.05289
Prkaca	2943.04	92.98	2340.11	135.92	-1.26	0.07194
Prkcdbp	194.16	10.36	254.56	15.83	1.31	0.07563
Psmb10	317.56	13.53	384.30	17.30	1.23	0.06385
Ptp4a3	3667.92	76.18	2862.68	155.45	-1.28	0.01446
Pxmp4	370.43	16.95	301.76	8.35	-1.21	0.05978
Rbfox1	828.94	55.93	593.70	16.39	-1.38	0.04169
Rcn3	212.77	5.37	257.95	7.41	1.21	0.09481
Rftn1	435.61	15.57	315.10	11.74	-1.38	0.00404
Rfxap	110.16	8.36	79.72	5.45	-1.32	0.08145
Rgma	617.76	19.13	353.39	38.60	-1.78	0.00339
Rgs5	5658.72	369.06	8388.59	360.73	1.49	0.00216
Rnase4	319.28	26.93	435.34	39.48	1.37	0.07306
Rnf123	796.14	9.39	641.79	45.44	-1.25	0.07441
Robo4	587.96	26.40	802.17	39.07	1.36	0.00866
RP23-148L8.3	77.13	9.58	115.85	8.61	1.54	0.05322
Rpl3	341.84	15.91	544.65	28.58	1.59	0.00064
Rpl3l	2610.52	109.40	1832.15	116.42	-1.43	0.00356
Rps2	2758.96	132.42	2276.13	45.64	-1.20	0.06258
Rragd	926.37	23.41	1112.13	27.33	1.20	0.04016
Sar1a	965.70	53.63	1160.96	21.36	1.21	0.08572
Scgb1c1	113.93	8.17	166.90	12.69	1.50	0.04179
Sept9	579.46	16.10	450.26	26.81	-1.29	0.03998
Serpinf1	269.97	17.50	331.33	11.04	1.24	0.08138
Serping1	1250.85	28.54	1685.30	73.56	1.35	0.00221
Sgk1	829.87	31.77	1241.36	81.84	1.49	0.00781
Sh2d3c	413.13	19.51	316.41	18.91	-1.30	0.07576
Slain2	1113.95	98.43	1495.51	72.06	1.36	0.05390
Slc25a20	1606.40	8.34	1223.01	72.58	-1.32	0.00746
Slc25a22	636.89	68.87	383.11	42.01	-1.65	0.01677
Slc25a23	712.34	25.79	538.49	30.24	-1.33	0.01600
Slc25a34	2369.36	19.43	1951.64	92.70	-1.22	0.07140
Slc25a39	1354.88	79.42	1115.69	40.03	-1.21	0.09095
Slc25a42	1091.43	64.54	650.10	41.08	-1.69	0.00087
Slc27a1	1758.05	73.16	1080.96	37.36	-1.62	0.00021
Slc28a2	153.05	15.53	102.09	4.39	-1.53	0.01534
Slc35f5	665.36	49.79	865.46	39.07	1.31	0.06151
Slc3a2	387.45	14.12	488.02	32.71	1.26	0.07953
Slc41a1	1014.16	21.40	801.44	10.04	-1.26	0.01324
Slc43a3	879.42	73.29	1221.23	68.57	1.40	0.03621
Slc4a3	3500.08	196.77	2696.02	56.08	-1.29	0.02590
Slc6a6	1532.31	109.27	2268.21	218.66	1.47	0.03169
Slmap	1430.29	106.08	1916.95	58.23	1.35	0.05515
Smarcd1	474.50	35.32	346.47	20.83	-1.36	0.04826

Smox	176.66	12.06	125.54	9.86	-1.40	0.05895
Smpd2	268.59	6.81	222.28	6.21	-1.20	0.05616
Sorbs2	3687.15	135.74	4570.05	179.96	1.24	0.06094
Sparc	4495.53	86.97	5509.13	142.79	1.23	0.02257
Spint2	196.34	10.63	143.44	10.16	-1.39	0.04074
Sptan1	2259.86	102.76	3190.31	191.57	1.41	0.01652
Srebf1	434.42	35.66	290.34	20.23	-1.47	0.02127
Srgn	251.58	9.09	369.83	23.66	1.46	0.00618
Stard10	186.33	11.94	126.27	12.87	-1.48	0.08788
Steap3	533.44	3.93	423.58	17.47	-1.26	0.07764
Svil	2848.68	114.75	3490.73	82.78	1.23	0.07441
Tagln2	607.50	12.79	771.38	55.71	1.26	0.08288
Tbc1d16	1277.24	29.81	931.48	33.49	-1.37	0.00112
Tead3	269.18	19.71	202.25	9.97	-1.32	0.09159
Tesc	767.40	63.94	465.53	24.70	-1.63	0.00185
Thbd	708.49	53.64	1021.41	40.32	1.45	0.00727
Thoc7	126.46	6.94	160.57	6.18	1.27	0.09623
Timp4	267.65	45.62	49.28	6.60	-5.43	0.00021
Tinagl1	543.62	22.36	1030.47	137.04	1.85	0.00512
Tm4sf1	365.54	24.65	671.78	57.57	1.84	0.00223
Tmem201	1488.19	70.47	1154.00	89.27	-1.29	0.06462
Tnfrsf12a	191.56	18.33	397.39	33.80	2.08	0.00044
Tpm2	224.54	18.21	382.26	48.99	1.64	0.03957
Trp53i11	505.16	53.07	168.41	30.90	-3.21	0.00021
Tsc22d4	2073.90	79.84	1648.39	94.60	-1.26	0.04519
Tspan13	459.92	18.21	347.24	15.41	-1.32	0.01644
Tspan7	988.70	23.54	659.93	9.40	-1.49	0.00033
Tspan9	734.90	17.59	898.13	62.50	1.21	0.09984
Ttc7	294.51	22.03	222.17	12.50	-1.31	0.05416
Tubb2a	160.48	8.95	213.61	6.62	1.35	0.09124
Ucp3	404.96	32.61	216.37	41.53	-2.10	0.01145
Ugp2	1920.33	103.60	2658.44	92.44	1.39	0.01481
Vegfb	3313.04	183.06	2607.90	155.06	-1.27	0.06049
Vtn	329.19	12.23	252.88	11.48	-1.32	0.03591
Whrn	522.52	51.79	379.86	14.52	-1.34	0.07534
Wnk2	1591.71	105.05	1126.12	112.32	-1.43	0.06248
Xirp2	5952.35	241.71	7931.81	287.78	1.33	0.02296
Ybx1	4816.43	103.44	3712.59	90.74	-1.29	0.00236
Ybx2	351.17	50.47	207.20	12.25	-1.64	0.04225
Ybx3	1746.85	74.85	1446.59	57.98	-1.20	0.06994
Ywhaq	380.36	20.45	478.38	21.35	1.25	0.06009
Zadh2	821.47	22.11	666.18	14.86	-1.23	0.01765
Zdhhc9	544.19	18.29	445.46	21.68	-1.22	0.08170
Zfp106	4920.12	281.10	6367.64	351.45	1.30	0.09735
Zfp46	535.84	28.55	424.67	6.18	-1.25	0.03063

Zmynd8	796.94	42.77	642.02	28.89	-1.23	0.09779
Znrf1	660.85	36.53	511.29	20.48	-1.29	0.04066
